# Spirooxindole 5‐(Trifluoromethyl)benzothiazole Hybrids: Synthesis, Crystal Structure, and Antioxidant Activity

**DOI:** 10.1002/ardp.70343

**Published:** 2026-09-28

**Authors:** Nida Karabag, Asu Busra Temizer‐Keser, Murat Pekmez, Elif Mertoğlu‐Kamalı, Filiz Betül Kaynak, Nurten Özsoy, Nazlı Arda, Nilgün Karalı

**Affiliations:** ^1^ Department of Pharmaceutical Chemistry, Faculty of Pharmacy Istanbul University Istanbul Turkey; ^2^ Department of Pharmaceutical Chemistry Institute of Health Sciences, Istanbul University Istanbul Turkey; ^3^ Department of Pharmaceutical Chemistry, Faculty of Pharmacy Istanbul University‐Cerrahpaşa Istanbul Turkey; ^4^ Department of Molecular Biology and Genetics, Faculty of Science Istanbul University Istanbul Turkey; ^5^ Department of Physical Engineering, Faculty of Engineering Hacettepe University Ankara Turkey; ^6^ Department of Biochemistry, Faculty of Pharmacy Istanbul University Istanbul Turkey

**Keywords:** antioxidant effect, benzothiazoline, cytotoxicity, spirooxindole, X‐ray analysis

## Abstract

Antioxidants neutralize free radicals to reduce oxidative stress and cellular damage, preventing various chronic diseases. In this study, the antioxidant activities of novel 5‐(trifluoromethyl)‐3*H*‐spiro[1,3‐benzothiazole‐2,3′‐indol]‐2′(1′*H*)‐ones (**4a–h**), along with the spirocyclic 2‐indolinones (**4i–m** and **5a–n**) previously synthesized by our group, were investigated for the first time, to find a multi‐modal antioxidant and to understand the impact of the trifluoromethyl group located at the 5‐position of the benzothiazole on antioxidant activity. For this purpose, the 2,2‐diphenyl‐1‐picrylhydrazyl (DPPH•) free radical scavenging, superoxide anion scavenging, ABTS^•+^ radical cation scavenging, trolox equivalent antioxidant capacity, ferric ion reduction antioxidant potential (FRAP), and lipid peroxidation (TBARs) assays were carried out. The cytotoxic effects of compounds (**4g**, **4h**, **4i**, **4j**, **4l**, **5a**, **5b**, and **5h)**, which showed promising in vitro antioxidant activity in at least two in vitro tests, were investigated in healthy human fibroblast (HFF‐1) cells. The effects of non‐toxic doses of these compounds on intracellular reactive oxygen species were also evaluated. As a result, it was determined that compound **4l** (R_1_ = 5‐F and R_2_ = H) exhibited greater radical scavenging activity than the other compounds tested. The IC_50_ of compound **4l** was 74.13 µM while compound **4g** (R_1_ = 5‐Cl‐7‐CH_3_ and R_2_ = H) was non‐toxic (IC_50_ > 200 μM). Compounds **4e** (R_1_ = 5‐Cl and R_2_ = H) and **4f** (R_1_ = 7‐Br and R_2_ = H), exhibited higher lipid peroxidation inhibitory activity than α‐tocopherol in vitro; however, the difference was not statistically significant.

## Graphical abstract

The antioxidant activities of novel 5‐(trifluoromethyl)‐3*H*‐spiro[1,3‐benzothiazole‐2,3′‐indol]‐2′(1′*H*)‐ones (**4a–h**), along with the previously synthesized spirocyclic 2‐indolinones (**4i–m** and **5a–n**), were investigated to find a multi‐modal antioxidant and to understand the impact of the trifluoromethyl group located at the 5‐position of the benzothiazole on antioxidant activity. Compound **4l** (R_1_ = 5‐F and R_2_ = H) was identified as the most potent antioxidant. Compound **4g** (R_1_ = 5‐Cl‐7‐CH_3_ and R_2_ = H) significantly reduced intracellular ROS in HFF‐1 at acceptable cytotoxicity.

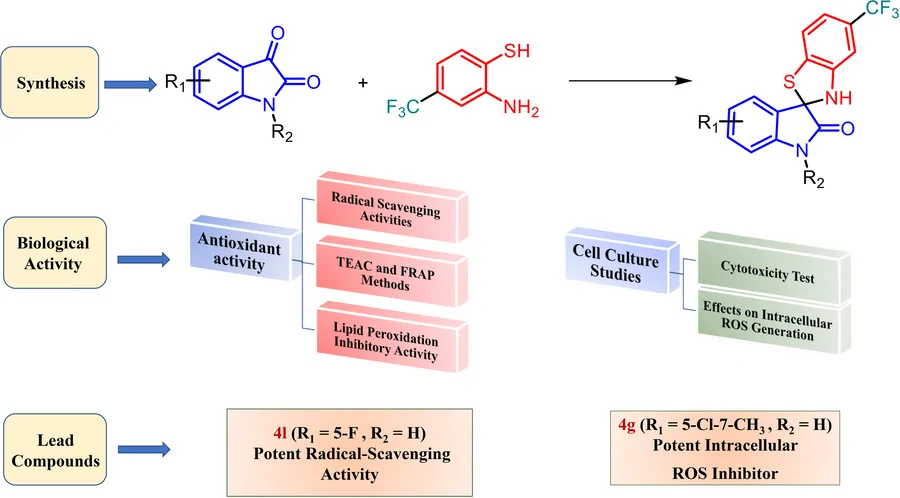

## 1 Introduction

Oxidation is a fundamental biochemical process for the continuation of life. Oxidation must occur to the extent necessary under physiological conditions. This is because oxidation triggers the formation of free radicals in the body. While free radicals have positive effects in small amounts, excessive concentrations can damage cells. When free radicals accumulate uncontrollably in the body, oxidative stress occurs [1, 2, 3, 4]. This stress can cause mutations that may result in tumors by damaging biomolecules such as lipids, proteins, enzymes, and DNA in cells and tissues. As a result, they contribute to the development of a range of disorders, including cancer, inflammatory diseases, diabetes, cardiovascular diseases, autoimmune diseases, neurodegenerative diseases, and aging [5].

Antioxidants neutralize free radicals by providing them with electrons. A balance between free radicals and antioxidants is necessary for optimal physiological function. Living organisms have a defense system that helps maintain oxidative stress in balance [3, 6]. In humans, indole‐3‐propionic acid (**I**) produced by intestinal bacteria and certain metabolites of tryptophan (e.g., serotonin (**II**), melatonin (**III**)) have been found to have free radical scavenging properties and are important indole derivatives with high antioxidant activity at low doses [7] (Figure 1). Most plants have a high natural antioxidant content, and their antioxidant effects come from enzymes, amino acids, vitamins, carotenoids, terpenoids, flavonoids, and so forth, in their structure [8]. In plants, brassinin (**IV**), spirobrassinin (**V**), and 1‐methoxyspirobrassinol (**VI**), which are indole derivatives of phytoalexins in the *Cruciferous* family, play an important role in defense against oxidative stress and pathogens, and have been proven to exhibit in vivo cytotoxic effects in solid tumors and leukemia cells (Figure 1). Gelsemin (**VII**), the most well‐known spirooxindole natural product belonging to the *Gelsemium* genus, has shown powerful antioxidant effects [9] (Figure 1).

**Figure 1:**
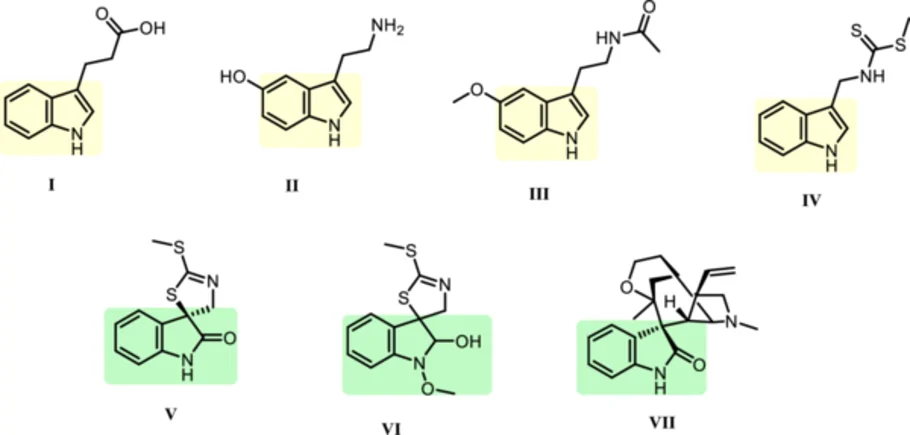
Chemical structures of antioxidant‐active indole derivatives.

Both natural and synthetic compounds bearing a spirooxindole skeleton have attracted increasing interest as free radical scavengers and antioxidants. Several studies in the literature have attributed the antioxidant activity to the presence of NH groups [10, 11, 12]. Kaur et al. synthesized a series of spiro[oxindole‐isoxazolidine] (**VIII**) derivatives and evaluated their antioxidant activities using the DPPH radical scavenging assay. All compounds exhibited stronger antioxidant effects than the reference standard BHT (IC_50_ = 5.37 μg/mL). The ethyl‐substituted derivative showed the highest potency, with an IC_50_ value of 0.44 μg/mL. Structure–activity relationship (SAR) analyses indicated that electron‐donating substituents enhanced antioxidant activity. Furthermore, the presence of an enolic OH group arising from the keto–enol tautomerism between the NH and CO groups at the 1‐position of the oxindole ring was identified as a key structural feature contributing to the observed activity [13]. Shrestha et al. synthesized a series of spirooxindole derivatives (**IX**) and evaluated their antioxidant potential using the ferric ion reduction antioxidant potential (FRAP) assay. SAR analysis revealed that derivatives with an unsubstituted indole ring or those bearing 5‐OCH_3_ or 7‐Cl substituents exhibited higher activity. Compounds containing electron‐donating or electron‐withdrawing groups at the *ortho* or *para* positions relative to the spirooxindolic NH were also found to possess greater potency. Moreover, the presence of an acetyl group on the spirooxindole nitrogen contributed positively to the antioxidant activity [14]. Nishtala et al. synthesized a series of spirooxindole–carbamate derivatives (**X**) and assessed their antioxidant properties using the DPPH assay. The compounds featured hydrogen, alkyl, or benzyl substituents at the 1‐position of the oxindole core. Among these, benzyl‐substituted analogs exhibited the highest radical‐scavenging activity, with potencies comparable to the reference antioxidant α‐tocopherol [15]. Pourshab et al. diastereoselectively synthesized a series of spirocyclopropane oxindole derivatives (**XI**) and evaluated their antioxidant potential using the DPPH radical scavenging assay. The results indicated that compounds bearing an NH group at the 1‐position of the indole ring exhibited higher antioxidant activity compared to those containing a triazole substituent. The most active derivatives were further examined for their in vitro cytotoxicity against HeLa cancer cell lines using the MTT assay. Among these, the compound bearing a chloro‐substituted phenyl ring showed the strongest cytotoxicity, with an IC_50_ of 4.50 ± 0.3 μg/mL [16]. In a study conducted in 2014, spiro[oxindole‐thiazolidine] derivatives carrying a fluorine group at the 5‐position of the 2‐indolinone ring were synthesized, and their antioxidant potentials were tested using the 2,2‐diphenyl‐1‐picrylhydrazyl (DPPH) radical scavenging assay. All compounds exhibited excellent antioxidant activity, and SAR studies revealed that the presence of the CF_3_ group and the spirooxindole ring, incorporated with thiazolopyrimidinethione (XII) or thiazolodiazaphosphorine (**XIII**), enhanced the activity [17]. Free radical scavenging potentials of spirooxindole‐dihydropyrimidinones were evaluated using DPPH and H_2_O_2_ tests. Compound (**XIV**), chlorine groups bearing at the positions 5‐ and 7‐ of the spirooxindole ring, was identified as the most potent compound compared to the reference ascorbic acid [18]. Figure 2 illustrates the chemical structures of the spirooxindole derivatives reported in these studies.

**Figure 2:**
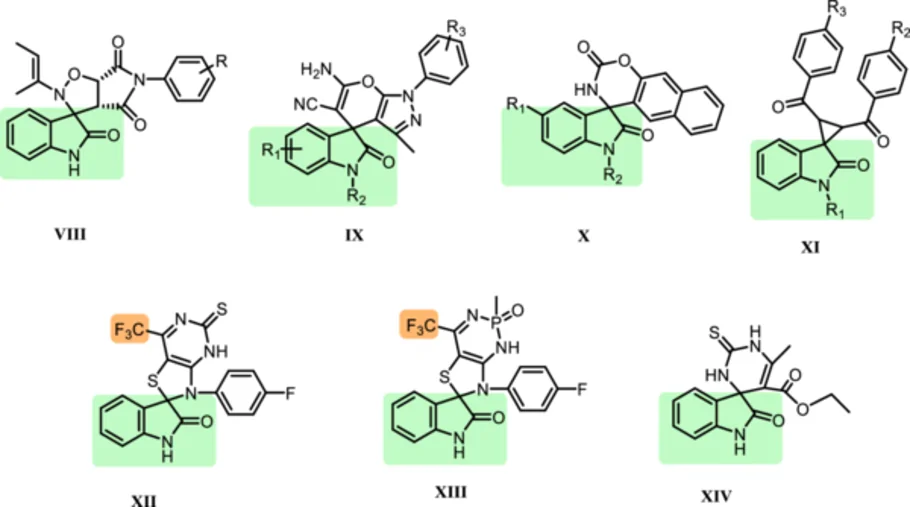
Chemical structures of spirooxindole derivatives.

A large number of 2‐arylbenzothiazole derivatives have been designed and synthesized due to their significant anticancer and antioxidant effects [19, 20, 21]. It is recorded that benzothiazinon and indolobenzothiazine derivatives are also obtained along with spirobenzothiazoline derivatives synthesized from the reaction of 1*H*‐indole‐2,3‐diones with 2‐aminobenzothiol. However, in the studies conducted by our group, the structures of the only product, spiro‐2‐indolinones, obtained by the reaction of 1*H*‐indole‐2,3‐diones and 2‐aminobenzothiol in ethanol were confirmed by spectral and X‐ray single crystal analyses [22, 23, 24, 25]. Its antioxidant effects were found to be superior to α‐tocopherol, and methyl and trifluoromethoxy substitutions at the 5‐position of the 2‐indolinone ring increased cytotoxicity [22]. In the study in which spiroindolinones bearing the 5‐chlorobenzothiazoline moiety were synthesized, it was noted that the fluorine‐substituted compound at the 5‐position of the 2‐indolinone ring was more effective as an antioxidant than α‐tocopherol [26].

The trifluoromethyl group is commonly used as a bioisostere in the design of new drug candidates, replacing functional groups such as methyl, chlorine, and nitro. Due to their similar dimensions, this group exhibits better compatibility with target proteins, possessing high electronegativity, strong lipophilicity, relatively low steric effects, and high permeability [27]. Furthermore, the trifluoromethyl group, which also enhances metabolic stability, modulates the binding affinity to target proteins via hydrogen bonds, thus contributing to improved pharmacological profiles. Consequently, it represents a valuable structural strategy in modern drug design [28].

In this study, the antioxidant capacities of newly synthesized spiroindolinone‐benzothiazole hybrids (**4a–h**) and similar spirocyclic 2‐indolinone compounds (**4i–m** and **5a–n**) previously synthesized by our group [25] were examined in order to compare their multifaceted effects and to investigate the contribution of the trifluoromethyl group located at the 5‐position of the benzothiazole ring to the antioxidant effect. The in vitro radical‐scavenging abilities, trolox‐equivalent antioxidant capacities, ferric ion‐reducing power, lipid peroxidation‐inhibitory activity, cytotoxicity toward healthy HFF‐1 cells, and effects on cellular reactive oxygen species (ROS) formation of these compounds were investigated. In addition, compliance with Lipinski's 5 rules (RO5) and ADME criteria of the compounds was evaluated in silico using the Qikprop module. The spirocyclic structure of compound **5h** was elucidated by spectral and single‐crystal X‐ray diffraction analyses.

## 2 Results and Discussion

### 2.1 Chemistry

In this study, 5‐(trifluoromethyl)‐3*H*‐spiro[1,3‐benzothiazole‐2,3′‐indol]‐2′(1′*H*)‐ones (**4a–h**) were newly synthesized by reaction of the 1*H*‐indole‐2,3‐diones (**1a–h**) with 2‐amino‐4‐(trifluoromethyl)benzenethiol (**3**) in ethanol [22]. The structures of the new compounds **4a–h** were confirmed by analytical and spectral (IR, ^1^H NMR, ^13^C NMR‐APT, HMBC‐2D, and LC‐MS) data. The general structures of compounds **4a–h** and other spirooxindole‐benzothiazoles (**4i–m** and **5a–n**) previously synthesized by our group are shown in Scheme 1 [25].

**Scheme 1:**
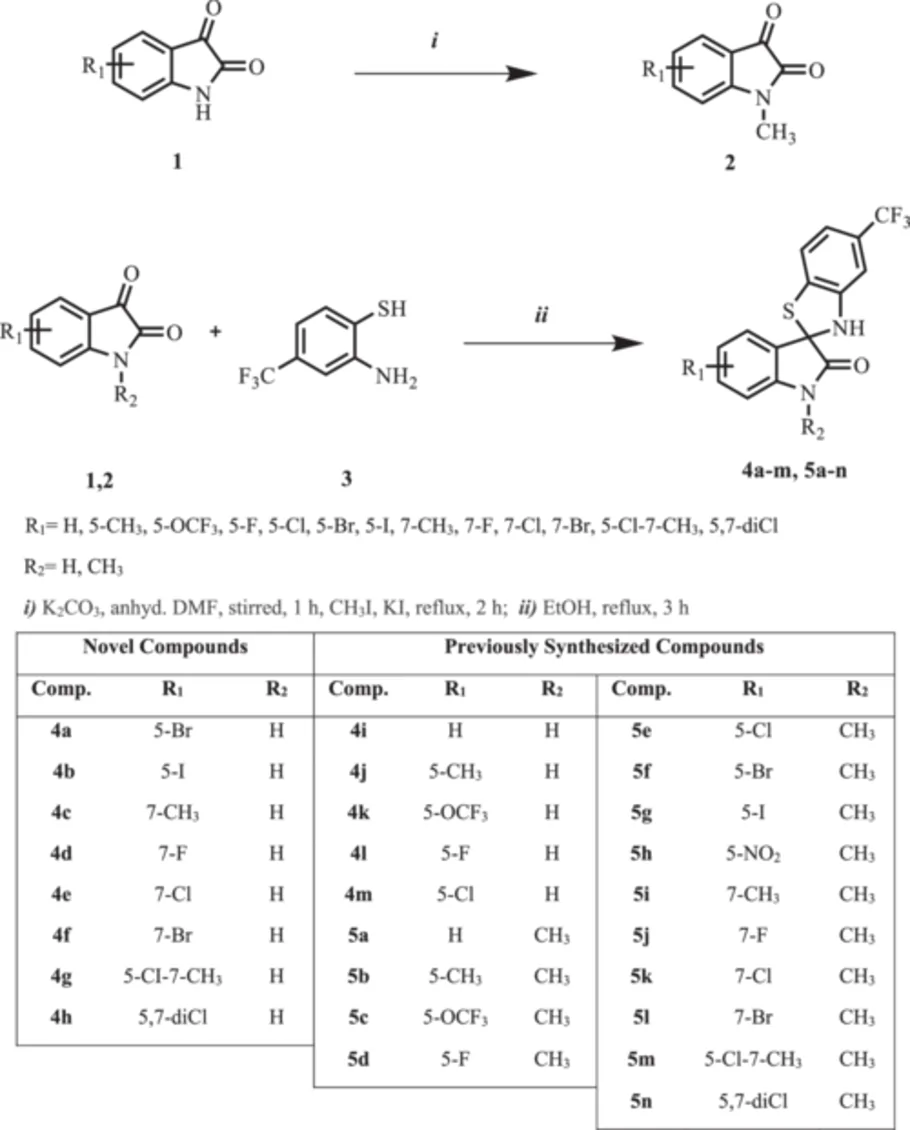
Synthesis of 5‐(trifluoromethyl)‐3*H*‐spiro[1,3‐benzothiazole‐2,3′‐indol]‐2′(1′*H*)‐ones (**4a–m** and **5a–n**).

In the IR spectra of compounds **4a–h**, indole and benzothiazole N─H stretching bands were observed in the 3369–3167 cm^−1^ region [29, 30, 31]. The fact that the C═O stretching band characterizing the ketone group of 1*H*‐indole‐2,3‐dione was not observed. The lactam group C═O stretching band, proving the accuracy of the spiroindolinone structure signaled in the region 1730–1701 cm^−1^ [32]. In the ^1^H NMR spectra of compounds **4a–h**, the NH proton signals of the benzothiazoline and the indole rings were observed at *δ* 7.60–7.77 and 10.48–11.11 ppm regions, respectively [22].

In the ^13^C NMR‐APT spectra of compound **4a–h** and the HMBC‐2D spectra of compound **4f**, the ketone C═O (C_3_) carbon signals (*δ* 181.5–184.5 ppm) of 1*H*‐indole‐2,3‐dione were not detected. Observation of lactam C═O (C_2_) carbon signals at *δ* 175.16–176.48 and spiro C resonances at *δ* 74.49–76.02 ppm were confirmed the spiroindolinone structures of the compounds [19, 22]. In the HMBC‐2D spectra of compound **4f**, the indole NH proton (*δ* 10.80 ppm on the ^1^H axis) interacted with indole C_7a_ (*δ* 141.19 ppm), C_3a_ (*δ* 131.71 ppm), and spiro (*δ* 76.02 ppm) carbons on the ^13^C axis. These findings further confirmed the spiroindolinone structure bearing benzothiazoline residues.

LC‐MS analyses were performed by positive (electrospray ionization [ESI]+) or negative (ESI−) ionization techniques. In the spectra of compounds **4a–g** taken in the positive mode, [M + H]^+^ peaks were detected as the main peak (except for compound **4g**). In the mass spectra of bromine‐substituted compounds **4a** and **4f** taken in positive mode, [M + H]^+^ and [M + H]^+^ + 2 peaks with a relative abundance ratio of approximately 1:1 arising from the 79 and 81 isotopes of the bromine atom were observed as the main peaks. In the mass spectrum of the chlorine‐substituted compound **4e** taken in the positive mode, the presence of [M + H]^+^ + 2 peak in a relative abundance ratio of about 3:1 originating from the [M + H]^+^ and chlorine isotopes 35 and 37 confirms the molecular weight and is observed as the main peak. In the negative mode mass spectrum of 5,7‐dichloro‐substituted compound **4h**, the [M‐H]^−^, [M‐H]^−^ + 2, and [M‐H]^−^ + 4 peaks in the isotopic ratio 9:6:1 confirm the molecular weight. The mass spectrum of the 5‐iodine substituted compound **4b**, taken in positive mode, showed [M + H]^+^ signals and [M + Na]^+^ and [M + 2Li]^+^ peaks of varying relative abundance, confirming its molecular weight and observed as the main peak.

### 2.2 X‐Ray Single‐Crystal Diffraction Analysis

Among the four derivatives that successfully yielded single crystals (**5h**, **5l**, **5m**, and **5n**) within this framework, compounds **5l**, **5m**, and **5n** had already been crystallographically characterized in our previous publications [24, 25]. Although all four compounds formed suitable crystals, compound **5h** was specifically chosen for structure elucidation in the present study because it demonstrated the highest antioxidant activity among them.

The compound **5h** crystallizes in the monoclinic space group *P*2_1_/*c* with *Z* = 4. Details of the data collection conditions and refinement parameters are summarized in Table 1. A Mercury drawing with atom numbering is shown in Figure 3 and selected bond lengths and angles are presented in Table 2 [33].

**Table 1:** Crystallographic data and structure refinement details for compound **5h**.

Crystal data	
Chemical formula	C_16_H_10_F_3_N_3_O_3_S
Formula weight	381.33
Crystal system, Space group	Monoclinic, P2_1_/c
Temperature (K)	293(2)
*a*, *b*, *c* (Å)	12.6667(6), 13.6311(8), 9.5110(4)
*β* (°)	92.369(4)
*V* (Å^3^)	1640.78(14)
*Z*	4
Radiation type	Mo Kα
µ (mm^−1^)	0.252
Crystal size (mm)	0.790 × 0.650 × 0.550
Data collection	
Diffractometer	STOE *IPDS* 2
T_min_, T_max_	0.826, 0.874
No. of measured, independent, and observed [I > 2σ(I)] reflections	12221, 3447, 2624
Rint	0.0510
Refinement	
R[F^2^ > 2σ(F^2^)], wR(F^2^), GOF	0.0370, 1.011, 1.025
No. of reflections	3447
No. of parameters	268
H‐atom treatment	H atoms treated by a mixture of independent and constrained refinement
Δρ_max_, Δρ_min_ (e Å^−3^)	0.224, −0.213

**Figure 3:**
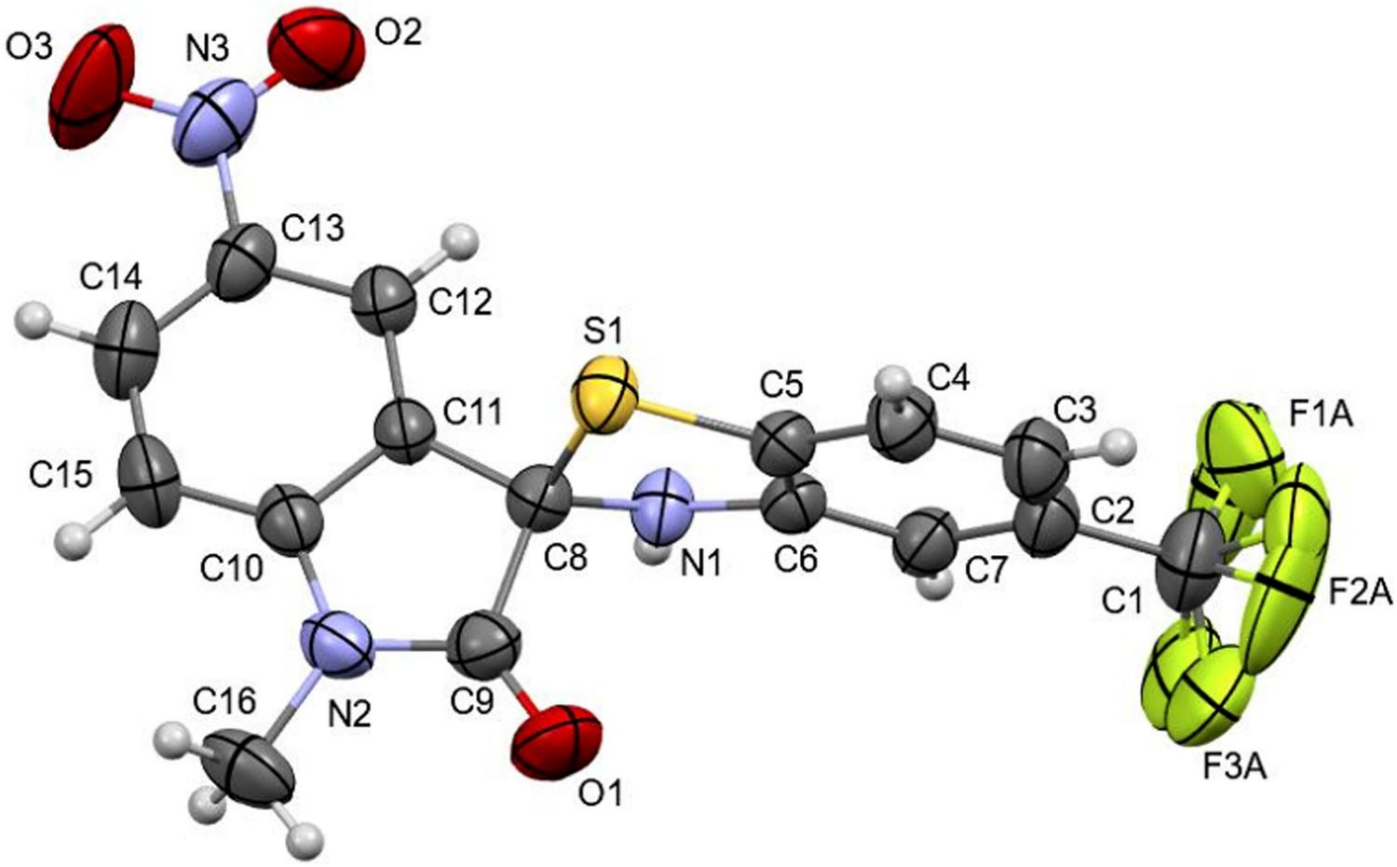
Mercury drawing of compound **5h** showing the atom‐numbering scheme. Thermal ellipsoids are drawn at the 50% probability level for non‐H atoms.

**Table 2:** Selected bond lengths (Å) and angles (°) for compound **5h**.

S1–C8	1.855(2)	C5–S1–C8	90.58(7)
S1–C5	1.761(2)	N1–C8–S1	104.07(10)
C5–C6	1.397(2)	C6–N1–C8	113.49(13)
N1–C8	1.447(2)	O1–C9–C8	126.42(16)
N1–C6	1.399(2)	O1–C9–N2	126.09(17)
C8–C9	1.553(2)	N2–C9–C8	107.49(13)
C8–C11	1.506(2)	C12–C13–C14	122.83(17)
C11–C10	1.400(2)	C13–N3–O2	118.88(15)
N2–C10	1.396(2)	C13–N3–O3	118.3(2)
N2–C9	1.370(2)	C9–N2–C16	123.5(2)
N2–C16	1.459(2)	C9–N2–C10	111.1(1)
O1–C9	1.210(2)		
F1a–C1	1.313(6)		
F2a–C1	1.305(6)		
F3a–C1	1.333(6)		
N1–H1	0.81(2)		

The hydrogen atom attached to the N1 was located in a difference Fourier map, and its coordinates and atomic displacement parameters were refined isotropically (N1 − H1 = 0.81(2) Å). The CF_3_ group is disordered in the structure, crystallizing in the monoclinic *P*2_1_/*c* space group. The structure solution revealed disorder in the CF_3_ group atoms, with occupancy factors of 0.61(2) for component A and 0.39(2) for component B. All remaining hydrogen atoms were placed geometrically, with C─H distances ranging from 0.93 to 0.97 Å, and with Uiso(H) values set to 1.5 Ueq(C) for methyl hydrogen atoms and 1.2 Ueq(C) for other hydrogen atoms. The crystal structure of compound **5h** is stabilized by intra‐ and inter‐molecular hydrogen bonds (Table 3). The packing and the hydrogen bonding interactions in the structure are shown in Figures 4 and 5.

**Table 3:** Hydrogen‐bond geometry (Å, °) for compound **5h**.

*D*—H···*A*	*D*—H	H···*A*	*D*···*A*	*D*—H···*A*
C7—H7···O2^i^	0.93	2.56	3.2896	136
C12—H12···O1^ii^	0.93	2.46	3.3883	174
C15—H15···F2a^iii^	0.93	2.35	3.1594	145
C16—H16B···O1	0.96	2.53	2.8994	103
Symmetry code: (i) 1 − *x*, −*y*, 1 − *z*; (ii) 1 − *x*, −1/2 + *y*, 1/2 − *z*; (iii) 1 + *x*, −1/2 − *y*, 1/2 + *z*				

**Figure 4:**
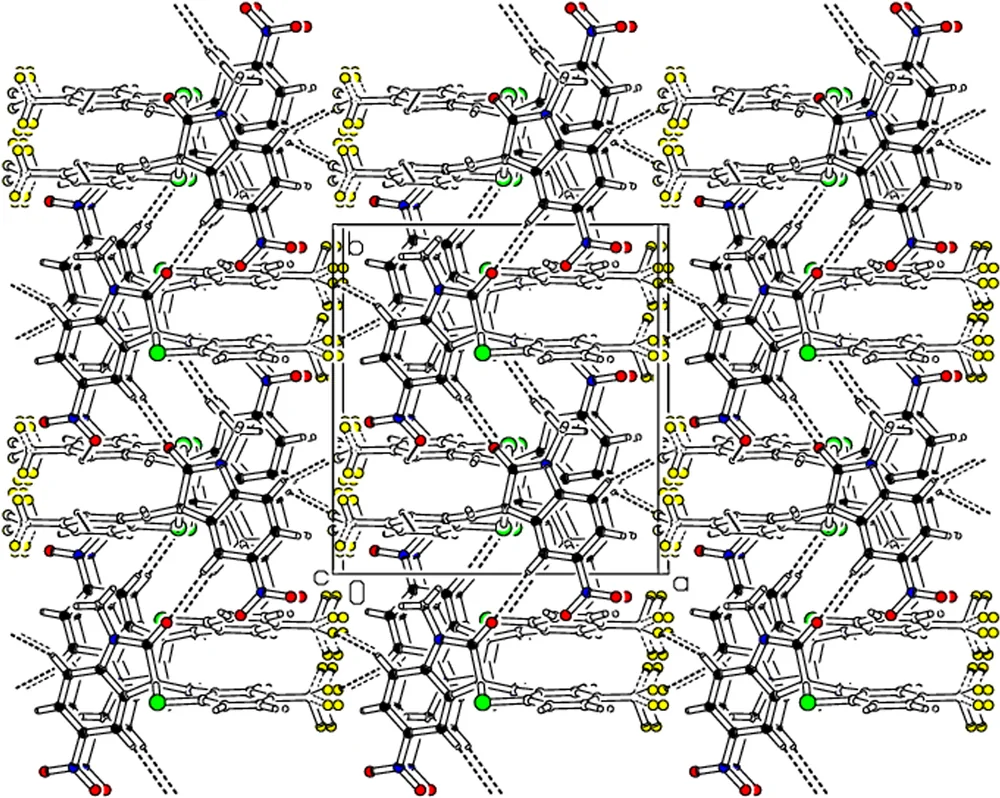
A view along the *c*‐axis showing the crystal packing and hydrogen‐bonding interactions in compound **5h**. Hydrogen bonds are shown as black‐and‐white dashed lines.

**Figure 5:**
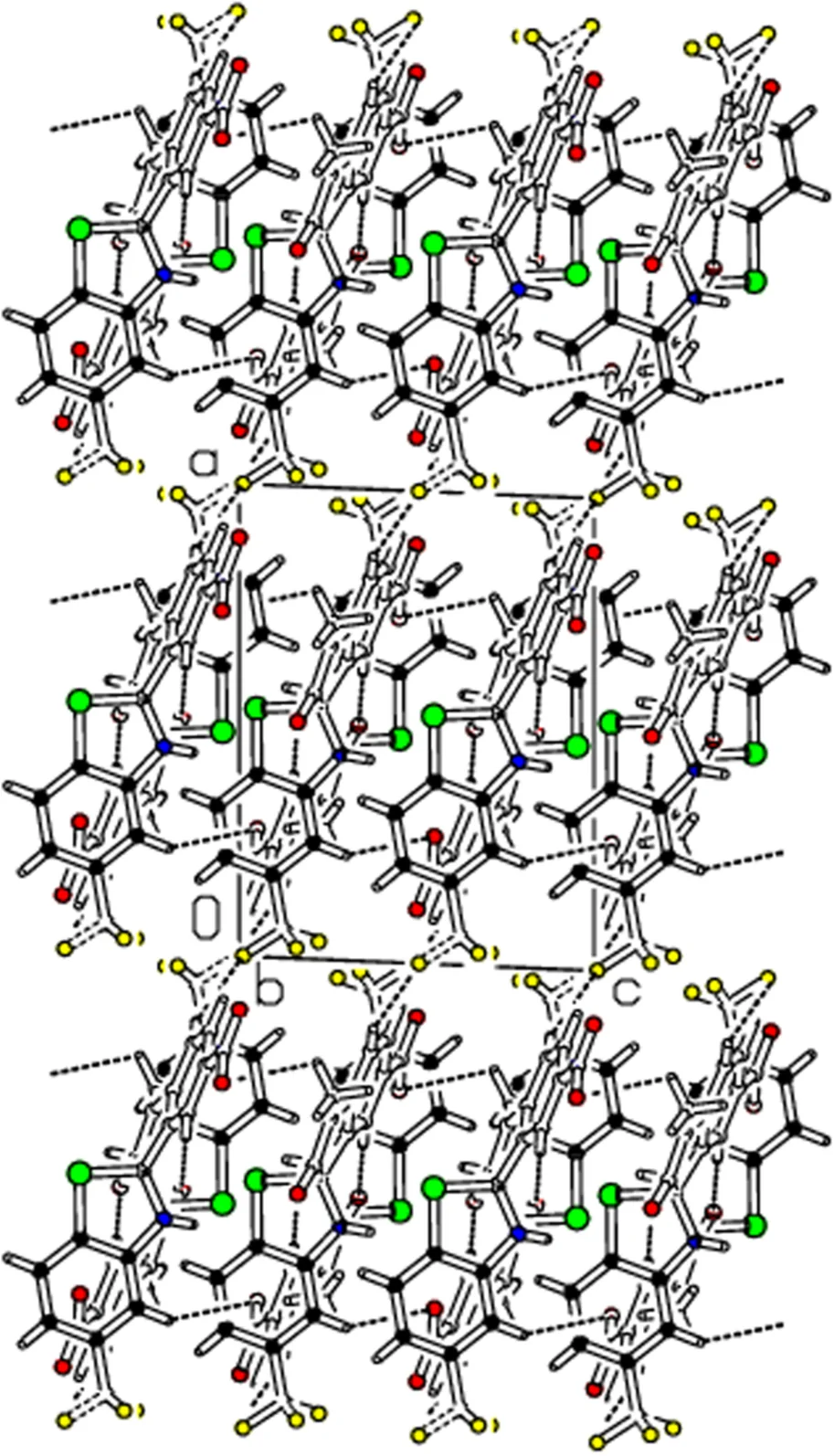
A view along the *b*‐axis showing the crystal packing and hydrogen‐bonding interactions in compound **5h**. Hydrogen bonds are shown as black‐and‐white dashed lines.

The asymmetric unit contains one molecule of 5′‐nitro‐1′‐methyl‐5‐(trifluoromethyl)‐3*H*‐spiro[1,3‐benzothiazole‐2,3′‐indol]‐2′‐on. The molecule adopts a spiro form, and it is not planar, as documented in previous studies [22, 23, 24, 25]. The indole and benzothiazole moieties are approximately perpendicular, with a dihedral angle of 90.00(3)° between them. Based on the puckering parameter values [34], the benzothiazole ring at C8 is twisted around the C8─S1 bond. All bond lengths and angles are within normal ranges and consistent with those reported in previous studies [22, 23]. The torsion angle N1─C8─C9─N2 is −135.63(13)°, indicating an anticlinal (‐ac) conformation, whereas the C11─C8─N1─C6 torsion angle is 150.20(14)°, indicating an antiperiplanar (ap) conformation. These findings are consistent with the geometry of related structures [22, 24].

The molecular structure of **5h** reveals that the trifluoromethyl group is disordered over two positions, indicating rotational flexibility commonly observed in CF_3_‐containing aromatic systems. The C─F bond lengths for the major disordered component [1.305(6)–1.333(6) Å] are within the expected range for related CF_3_‐substituted spiro systems. Furthermore, the bond angles around the spiro center [102.9(9)–108.9(7)°] suggest a slightly distorted yet essentially preserved tetrahedral geometry. Although minor deviations from the ideal tetrahedral value are observed, no significant conformational distortion is evident when compared with structurally related trifluoromethyl‐substituted spirooxindole‐benzothiazoline derivatives reported previously [24].

To validate the geometric reliability of the in silico studies, a structural superposition between the experimental X‐ray crystal structure and the predicted lowest‐energy conformation of the title compound was performed using Schrödinger Maestro. The structural alignment demonstrated a high geometric correlation with an RMSD value of 1.5670 Å, as shown in Supporting Information: S1.

### 2.3 Antioxidant Activity

In this study, the antioxidant activities of the compounds **4a–m** and **5a–n** were evaluated using in vitro methods targeting different mechanisms. The free radical scavenging capacities of the compounds were determined using the DPPH^•^, superoxide anion, and ABTS^•+^ radicals. The Trolox equivalent antioxidant capacity (TEAC) expressed as Trolox equivalents quantifies the electron‐donating capacity of the compounds compared with that of Trolox. The FRAP assay measured the compounds' electron‐donating capacity for metal ion reduction. The anti‐lipid peroxidation assay evaluated the compounds' ability to prevent oxidative lipid degradation. BHT and α‐tocopherol were used as standards, and Table 4 presents the antioxidant activities of the studied spirooxindole compounds, expressed as EC_50_, TEAC, FRAP values, and lipid peroxidation inhibitory activities.

**Table 4:** Antioxidant activities of compound **4a–m** and **5a–n** as EC_50_, TEAC, FRAP, and TBARs values.

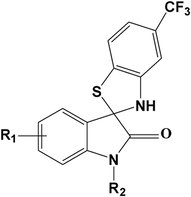								
Comp.	R_1_	R_2_	EC_50_ (mM)*			TEAC (mM Troloks)**	FRAP (mM Fe^2+^)***	% Inhibition
			DPPH	ABTS	Superoxide			TBARS (Lipid peroxidation)****
**4a**	5‐Br	H	2.07 ± 0.06^a^, ^b^	2.04 ± 0.07^a^, ^b^	1.91 ± 0.02^a^, ^b^	**1.73** ± **0.02** a, b	**3.06** ± **0.06** a, b	37.13 ± 11.85
**4b**	5‐I	H	1.98 ± 0.04^a^, ^b^	1.93 ± 0.25^a^, ^b^	1.78 ± 0.03^a^, ^b^	**1.80** ± **0.02** a, b	**3.08** ± **0.07** a, b	37.88 ± 5.04
**4c**	7‐CH_3_	H	2.29 ± 0.04^a^, ^b^	2.37 ± 0.11^a^, ^b^	2.17 ± 0.02^a^, ^b^	**1.68** ± **0.03** a, b	**2.49** ± **0.06** a, b	36.59 ± 7.85
**4d**	7‐F	H	2.38 ± 0.01^a^, ^b^	2.34 ± 0.09^a^, ^b^	2.17 ± 0.03^a^, ^b^	**1.62** ± **0.01** a, b	**2.40** ± **0.09** a, b	35.07 ± 2.15
**4e**	7‐Cl	H	2.28 ± 0.02^a^, ^b^	2.23 ± 0.19^a^, ^b^	2.18 ± 0.07^a^, ^b^	**1.63** ± **0.01** a, b	**2.53** ± **0.09** a, b	**53.09** ± **2.97**
**4f**	7‐Br	H	2.26 ± 0.09^a^, ^b^	2.35 ± 0.07^a^, ^b^	2.21 ± 0.01^a^, ^b^	**1.67** ± **0.02** a, b	**2.52** ± **0.04** a, b	**51.49** ± **8.28**
**4g**	5‐Cl‐7‐CH_3_	H	**1.16** ± **0.06** b	**1.04** ± **0.10** a, b	**1.09** ± **0.02** a, b	**1.90** ± **0.04** a, b	**3.03** ± **0.02** a, b	**45.81** ± **4.33**
**4h**	5,7‐diCl	H	**1.14** ± **0.02** b	**1.01** ± **0.08** a, b	**1.15** ± **0.03** b	**1.94** ± **0.01** a, b	**3.02** ± **0.09** a, b	**48.21** ± **10.67**
**4i**	H	H	**1.01** ± **0.08** b	**0.97** ± **0.03** a, b	1.19 ± 0.07^b^	1.23 ± 0.03^b^	**1.80** ± **0.09** a, b	**40.59** ± **12.68**
**4j**	5‐CH_3_	H	**1.05** ± **0.05** b	**1.17** ± **0.01** b	1.35 ± 0.08^a^, ^b^	1.13 ± 0.04^a^, ^b^	**1.76** ± **0.01** a, b	38.83 ± 12.14
**4k**	5‐OCF_3_	H	**1.21** ± **0.10** b	**1.15** ± **0.03** b	1.33 ± 0.11^a^, ^b^	1.26 ± 0.01^b^	**1.24** ± **0.06** a, b	39.36 ± 11.92
**4l**	5‐F	H	**0.43** ± **0.05** a, b	**0.73** ± **0.04** a, b	**0.89** ± **0.07** a	**1.70** ± **0.02** a, b	**3.00** ± **0.09** a, b	**44.16** ± **3.69**
**4m**	5‐Cl	H	1.58 ± 0.06^a^, ^b^	2.13 ± 0.20^a^, ^b^	1.76 ± 0.10^a^, ^b^	0.85 ± 0.09^a^, ^b^	**1.16** ± **0.06** a, b	34.41 ± 7.38
**5a**	H	CH_3_	**0.92** ± **0.08** a, b	**1.10** ± **0.11** a, b	**1.06** ± **0.16** a, b	**1.33** ± **0.06** a, b	**1.32** ± **0.13** a, b	29.03 ± 6.49
**5b**	5‐CH_3_	CH_3_	**1.15** ± **0.11** b	**1.22** ± **0.22** b	1.40 ± 0.08^a^, ^b^	1.27 ± 0.02^b^	0.91 ± 0.05^b^	24.50 ± 5.78
**5c**	5‐OCF_3_	CH_3_	1.59 ± 0.03^a^, ^b^	1.71 ± 0.06^a^, ^b^	1.79 ± 0.06^a^, ^b^	1.17 ± 0.02^b^	0.44 ± 0.02^a^, ^b^	19.79 ± 6.38
**5d**	5‐F	CH_3_	1.68 ± 0.09^a^, ^b^	1.61 ± 0.13^a^, ^b^	1.53 ± 0.05^a^, ^b^	1.04 ± 0.10^a^, ^b^	0.87 ± 0.22^b^	25.36 ± 3.92
**5e**	5‐Cl	CH_3_	1.30 ± 0.10^a^, ^b^	1.37 ± 0.18^b^	1.37 ± 0.07^a^, ^b^	1.08 ± 0.08^a^, ^b^	0.68 ± 0.04^a^, ^b^	24.63 ± 9.51
**5f**	5‐Br	CH_3_	1.34 ± 0.10^a^, ^b^	1.30 ± 0.16^b^	1.38 ± 0.05^a^, ^b^	1.06 ± 0.09^a^, ^b^	0.54 ± 0.04^a^, ^b^	20.77 ± 8.43
**5g**	5‐I	CH_3_	**1.23** ± **0.11** b	1.49 ± 0.07^a^, ^b^	1.42 ± 0.09^a^, ^b^	1.06 ± 0.07^a^, ^b^	0.46 ± 0.01^a^, ^b^	19.97 ± 9.46
**5h**	5‐NO_2_	CH_3_	**1.15** ± **0.06** b	1.60 ± 0.11^a^, ^b^	1.22 ± 0.05^b^	1.23 ± 0.03^b^	0.55 ± 0.01^a^, ^b^	18.64 ± 3.34
**5i**	7‐CH_3_	CH_3_	1.69 ± 0.04^a^, ^b^	1.88 ± 0.08^a^, ^b^	1.74 ± 0.05^a^, ^b^	1.10 ± 0.03^a^, ^b^	0.64 ± 0.02^a^, ^b^	16.06 ± 5.49
**5j**	7‐F	CH_3_	1.66 ± 0.13^a^, ^b^	1.88 ± 0.17^a^, ^b^	1.76 ± 0.08^a^, ^b^	1.10 ± 0.06^a^, ^b^	0.68 ± 0.01^a^, ^b^	22.81 ± 8.66
**5k**	7‐Cl	CH_3_	1.57 ± 0.03^a^, ^b^	1.83 ± 0.05^a^, ^b^	1.67 ± 0.06^a^, ^b^	1.09 ± 0.05^a^, ^b^	0.48 ± 0.04^a^, ^b^	24.68 ± 7.82
**5l**	7‐Br	CH_3_	1.56 ± 0.08^a^, ^b^	1.83 ± 0.02^a^, ^b^	1.79 ± 0.04^a^, ^b^	1.09 ± 0.01^a^, ^b^	0.42 ± 0.01^a^, ^b^	22.50 ± 1.28
**5m**	5‐Cl‐7‐CH_3_	CH_3_	1.69 ± 0.03^a^, ^b^	1.85 ± 0.05^a^, ^b^	1.72 ± 0.06^a^, ^b^	1.09 ± 0.03^a^, ^b^	0.37 ± 0.01^a^, ^b^	21.67 ± 7.75
**5n**	5,7‐diCl	CH_3_	1.58 ± 0.04^a^, ^b^	1.87 ± 0.16^a^, ^b^	1.72 ± 0.05^a^, ^b^	1.09 ± 0.06^a^, ^b^	0.30 ± 0.02^a^, ^b^	18.90 ± 4.72
**BHT**			1.20 ± 0.02^b^	1.28 ± 0.03^b^	1.18 ± 0.03^b^	1.30 ± 0.02^b^	0.98 ± 0.01^b^	−
**α‐Tocopherol**			0.17 ± 0.01	0.61 ± 0.01	0.9 ± 0.04	2.02 ± 0.01	3.75 ± 0.05	46.93 ± 3.43

*Note:* Data were presented as mean ± standard deviation. A significance level of *p* < 0.05 was considered. The TEAC and FRAP values of the compounds, tocopherol, and BHT were determined at a concentration of 2.5 mM.

^a^ Values significantly different from the BHT value.

^b^ Values significantly different from the α‐tocopherol value.

* EC_50_ values were expressed as the antioxidant amount required to scavenge 50% of DPPH, superoxide anion, and ABTS radical cation.

** TEAC values were expressed as mM Trolox equivalents.

*** FRAP values were expressed as mM Fe (II) ion equivalents.

**** TBARs, as adducts of MDA with 2‐thiobarbituric acid, represented the % inhibition of lipid peroxidation.

#### 2.3.1 Radical Scavenging Capacity

The compounds showed similar efficacy in scavenging DPPH•, ABTS^•+^ and superoxide anion radicals, with EC_50_ values of 0.43–2.38, 0.73–2.35 and 0.89–2.21 mM, respectively. The tested several compounds demonstrated comparable or higher radical scavenging capacities compared to BHT, with EC_50_ values of 1.20, 1.28, and 1.18 mM, respectively (*p* ≤ 0.05). However, the radical scavenging capacities of the compounds were lower than that of tocopherol, with EC_50_ values of 0.17, 0.61, and 0.49 mM, respectively (*p* < 0.05). It has been determined that the most effective DPPH^•^, ABTS^•+^, and superoxide anion radical scavenger compound is **4l** (R_1_ = 5‐F and R_2_ = H), with EC_50_ values of 0.43, 0.73, and 0.89 mM, respectively. It was observed that the active oxidant activity is significantly decreased by replacing the fluorine group with halogens such as chlorine, bromine, or iodine, by replacing at the 7‐position of the indole ring, or by methyl substitution at the 1‐position of the indole ring.

When the data in Table 4 was examined, it was seen that compounds **4g**, **4h**, **4i**, **4j**, **4k**, **5a, 5b**, **5g**, and **5h** had similar effects as BHT. Test results showed that the radical scavenging capacity increased significantly with hydrogen, methyl, trifluoromethoxy, and nitro substitutions, especially fluorine substitution, at the 5‐position of the indole ring, as well as disubstituted compounds at the positions 5‐ and 7‐ of the indole ring. However, it was found that the antioxidant potency was significantly decreased by methyl or halogen substitutions at the 7‐position of the indole ring. While methyl substitution at the 1‐position of the indole ring significantly decreased the antioxidant activity of the 5‐fluorine and 5‐trifluoromethyl substituted compounds, and 5,7‐disubstituted compounds, it did not significantly change the activities of the 5‐unsubstituted and 5‐methyl substituted compounds. This substitution caused a significant increase in the antioxidant activities of all other 5‐substituted and 7‐substituted compounds.

#### 2.3.2 Antioxidant Capacity via TEAC and FRAP Methods

The TEAC values of the tested compounds ranged from 0.85 to 1.94 mM, whereas the FRAP values were distributed over a broader range of 0.30–3.08 mM. Although none of the synthesized derivatives surpassed the maximum antioxidant capacity of α‐tocopherol (TEAC = 2.021 mM, FRAP = 3.75 mM), a considerable number of compounds belonging to series **4** (R_2_ = H) exhibited significantly higher or comparable activities relative to the synthetic reference antioxidant BHT (TEAC = 1.30 mM; FRAP = 0.98 mM).

In the TEAC assay, the most potent compounds were identified as compounds **4h** (R_1_ = 5,7‐diCl and R_2_ = H) and **4g** (R_1_ = 5‐Cl,7‐CH_3_ and R_2_ = H), with values of 1.94 and 1.90 mM, respectively. In addition, compounds **4a** (R_1_ = 5‐Br and R_2_ = H) and **4b** (R_1_ = 5‐I and R_2_ = H) also showed strong radical scavenging capacities with values of 1.73 and 1.80 mM, respectively. These findings indicate that halogen substitution at the 5‐position of the indole ring, particularly with heavier halogens such as bromine and iodine, enhanced radical scavenging activity. Furthermore, simultaneous substitution at the 5‐ and 7‐positions of the indole ring provides an additional favorable contribution to antioxidant capacity. In contrast, compounds of series **5** generally exhibited reduced TEAC activities, with values mainly clustered between 1.04 and 1.33 mM. Among this series, only compound **5a** (R_1_ = H and R_2_ = CH_3_) with values of 1.33 mM retained activity comparable to BHT.

The FRAP assay results further supported the superiority of series **4** (R_2_ = H) compounds. Most derivatives from this series displayed ferric reducing capacities higher than BHT and approaching that of α‐tocopherol. The most active compounds in this assay were compounds **4a** (R_1_ = 5‐Br and R_2_ = H), **4b** (R_1_ = 5‐I and R_2_ = H), **4g** (R_1_ = 5‐Cl,7‐CH_3_ and R_2_ = H), and **4l** (R_1_ = 5‐F and R_2_ = H) with FRAP values of 3.06, 3.08, 3.03, and 3.00 mM, respectively. These results suggest that halogen substituents strongly contribute to electron‐transfer ability and ferric ion reducing potential. On the other hand, compounds belonging to series **5** (R_2_ = CH_3_) showed significantly diminished FRAP activities. Although the unsubstituted compound **5a** (R_1_ = H and R_2_ = CH_3_) exhibited moderate activity with a FRAP value of 1.32 mM, substitution of the indole ring led to a marked decrease in reducing capacity. This effect was particularly evident in compounds **5m** (R_1_ = 5‐Cl,7‐CH_3_ and R_2_ = CH_3_) and **5n** (R_1_ = 5,7‐diCl and R_2_ = CH_3_), which displayed reduced FRAP values of 0.37 and 0.30 mM, respectively.

TEAC and FRAP results suggest that an unsubstituted indole N─H (series **4**) is crucial for antioxidant activity due to its hydrogen‐donating ability, whereas *N‐*methylation (series **5**) reduces activity by eliminating this key feature and introducing steric hindrance. It is also suggested that 5,7‐disubstitution, as well as bromine or iodine substituents, may enhance activity by providing a more favorable steric and electronic environment. The antioxidant capacities of the compounds, as measured by TEAC and FRAP assays, are presented in Figure 6.

**Figure 6:**
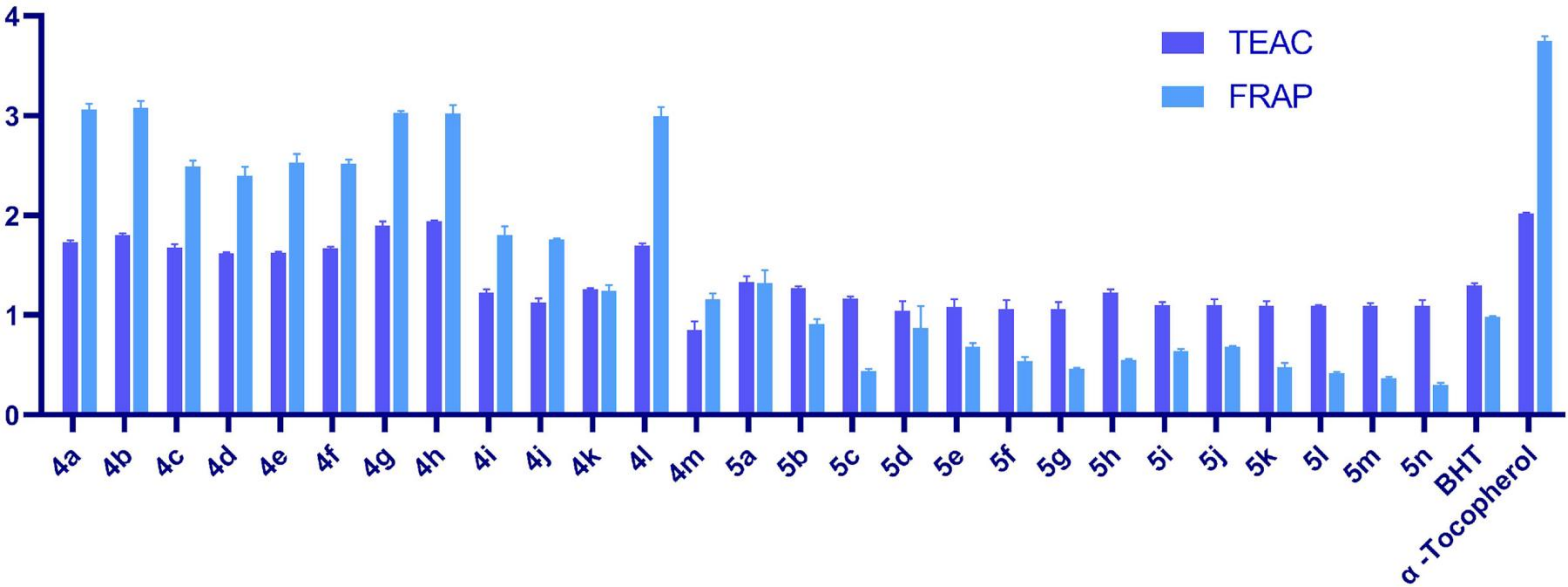
Antioxidant capacities of compounds **4a–m** and **5a–n** evaluated using TEAC and FRAP assays.

#### 2.3.3 Lipid Peroxidation Inhibitory Activity

Lipid peroxidation inhibitory (anti‐lipid peroxidation) activities of compounds **4a–m** and **5a–n**, as well as α‐tocopherol, at their 5 mM concentration, are presented in Table 4. Multiple comparisons using Dunnett's post hoc test showed that compounds **4e** (53.09%), **4f** (51.49%), and **4h** (48.21%) exhibited higher activity than α‐tocopherol (46.93%), although this difference was not statistically significant (Figure 7). Similarly, compounds **4g** (45.81%), **4i** (40.59%), and **4l** (44.16%) were classified as active and exhibited comparable profiles to the reference, while **4a–4d**, **4j**, **4k**, and **4m** demonstrated moderate activity with EC_50_ values ranging from 39.36% to 34.41%. In contrast, the **5**‐series compounds, except **5a**, exhibited significantly lower activity than α‐tocopherol (*p* < 0.01 or *p* < 0.05; see Figure 7). These results demonstrate that the compounds unsubstituted at the 1‐position of the indole ring (**4a–m**) exhibit a higher lipid peroxidation inhibitory capacity compared to the derivatives carrying a methyl group at the same position (**5a–n**). 7‐Chlorine, 7‐bromine, and 5,7‐dichlorine substituted compounds showed higher inhibitory effects than α‐tocopherol. The inhibitory effects were observed slight decrease with hydrogen, 5‐fluorine, and 5‐chloro‐7‐methyl substitutions. The findings indicate that compounds **4e**, **4f**, **4g**, **4h**, **4i**, and **4l**, which inhibit lipid peroxidation by 40%≤, stand out as the most promising antioxidant candidates. However, compounds **4e** and **4f** did not exhibit significant antioxidant activity in other in vitro tests, as did those with intermediate activity (**4a–d**), and thus were excluded from biological tests.

**Figure 7:**
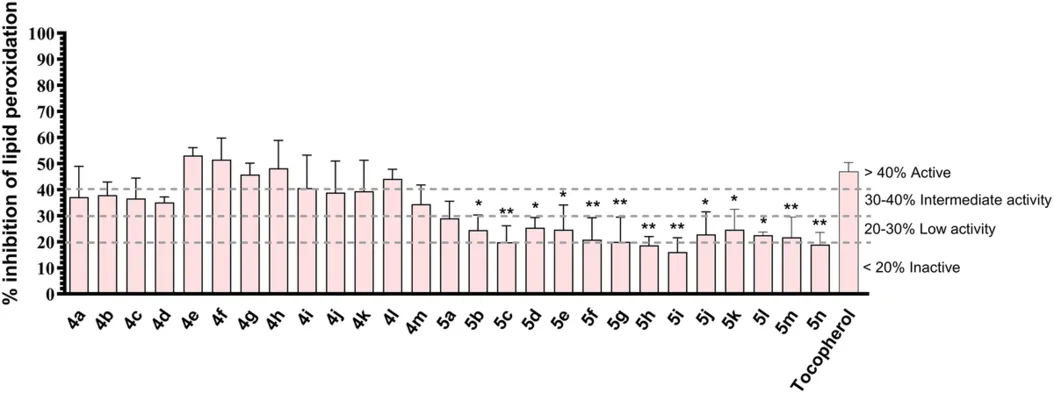
Comparison of the anti‐lipid peroxidation activity of compounds **4a–m** and **5a–n**. Samples demonstrating more than 40% inhibition were classified as active, while those showing 30%–40% inhibition were regarded as having intermediate activity. Samples exhibiting 20%–30% inhibition were considered to have low activity, and anything below 20% inhibition was labeled as inactive [35]. The test concentration for all samples and the reference compound, α‐tocopherol, was 5 mM. Data are expressed as mean ± SD in Table 4. Statistical significance was determined by one‐way ANOVA followed by Dunnett's multiple comparison test using α‐tocopherol as the reference antioxidant. *p < 0.05 and **p < 0.01 versus α‐tocopherol.

It was evident that all the compounds showed antioxidant activity and might be able to afford protection against oxidative damage by reducing free iron (III) ions. The investigation of the antioxidant action of compounds in vitro showed that all the compounds showed radical scavenging activities, and the antioxidant activities of these compounds might be attributed to their hydrogen or electron‐transfer abilities. These compounds also possess the antioxidative potential for chain‐breaking inhibition of lipid peroxidation. It was concluded that compound **4l** (R_1_ = 5‐F, R_2_ = H) is the most effective hydrogen and electron donor and can be considered as the best antioxidant amongst the compounds selected for the study.

### 2.4 Cell Culture Studies

#### 2.4.1 Cytotoxicity Test

Cytotoxicities of selected compounds **4g**, **4h**, **4i**, **4j**, **4l**, **5a**, **5b**, and **5h**, according to their relatively high in vitro antioxidant activities in two or more tests, were assessed on healthy human fibroblast HFF‐1 cells. Cell viability plots are presented in Figure 8, and the IC_50_ values are provided in Table 5. The effects of the compounds on cell viability were investigated by MTT assay. Since total mitochondrial activity correlates with the number of viable cells in most cell populations, this assay is widely used to measure the in vitro cytotoxic effects of pure substances or extracts on cell lines. Compounds **4g**, **5a**, **5b**, and **5h** showed no cytotoxicity in HFF‐1 cells, with IC_50_ values exceeding 200 μM. Compounds **4h**, **4i**, **4j**, and **4l** displayed slightly increased cytotoxicity, with IC_50_ values of 92.26, 141.26, 70.79, and 74.13 μM, respectively. The compounds can be ordered by cytotoxicity from highest to lowest as **4j** > **4l** > **4h** > **4i** > **4g**, **5a**, **5b**, **5h**. It appears that the safest compounds among the tested samples are the last four. These data showed that cytotoxic effects were significantly decreased by 1‐methyl substitution.

**Figure 8:**
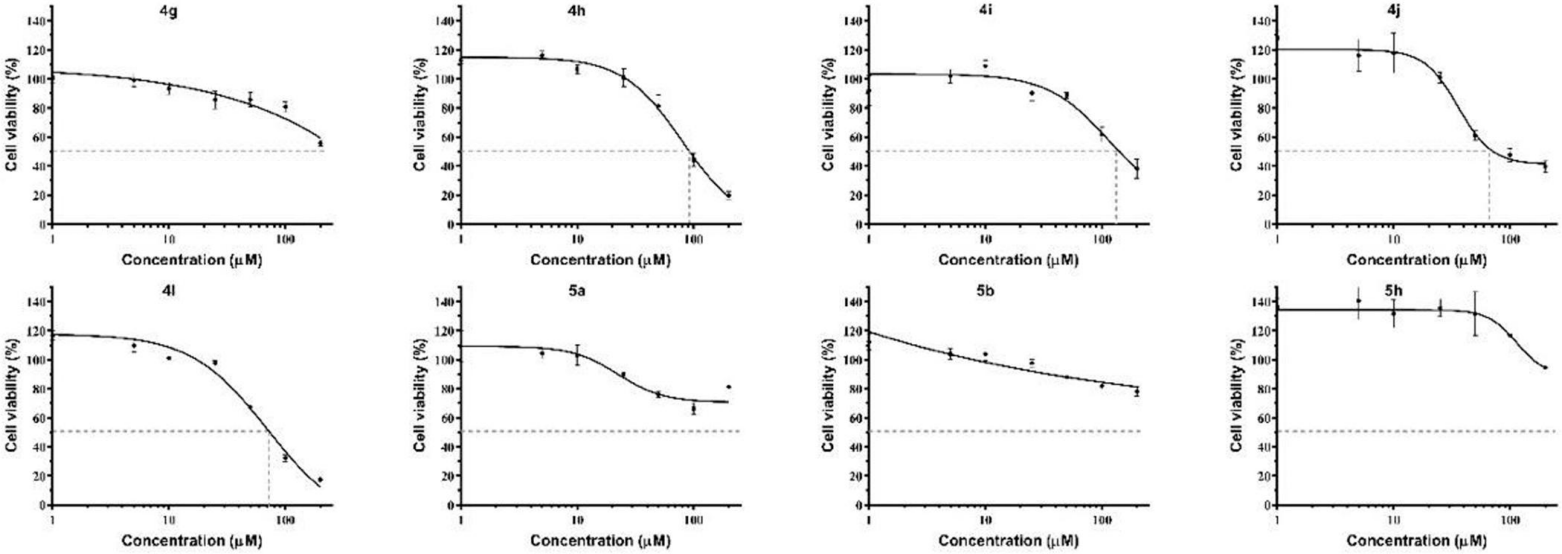
Cell viability (% of control) plots of the selected compounds in HFF‐1 cells.

**Table 5:** Cytotoxic effects of the selected compounds on HFF‐1 cells.

Compound	R_1_	R_2_	IC_50_ (µM)
**4g**	5‐Cl‐7‐CH_3_	H	> 200
**4h**	5,7‐diCl	H	92.26 ± 1.24
**4i**	H	H	141.26 ± 2.53
**4j**	5‐CH_3_	H	70.79 ± 1.13
**4l**	5‐F	H	74.13 ± 1.30
**5a**	H	CH_3_	> 200
**5b**	5‐CH_3_	CH_3_	> 200
**5h**	5‐NO_2_	CH_3_	> 200

#### 2.4.2 Effects on Intracellular ROS Generation

To evaluate the effects of selected compounds **4g**, **4h**, **4i**, **4j**, **4l**, **5a**, **5b**, and **5h** against hydrogen peroxide–induced ROS generation in HFF‐1 cells, a spectrofluorometric 2′,7′‐dichlorodihydrofluorescein diacetate (H_2_DCFDA) assay was employed. The data showing the relative fold increase or decrease compared with the control are presented in Figure 9. Compounds **4g**, **4h**, **4i**, **4l**, and **5a** showed a tendency to reduce ROS levels by a range of 0.42 to 0.30 fold compared to the control group. In contrast, compounds **4j**, **5b**, and **5h** tended to increase ROS levels within the range of 0.04–0.30 fold. While compounds **4h** (0.42) and **4l** (0.40) exhibited the highest numerical ROS inhibitory activity among the tested derivatives, none of the differences between compounds reached statistical significance (*p* > 0.05). Nevertheless, these findings point to the potential ROS‐inhibiting activity of the non‐substituted 5,7‐dichloro derivative **4h** and 5‐fluoro derivative **4l** at the 1‐position. Besides, the intracellular ROS‐lowering tendency of **4g**, **4h**, **4i**, and **4l** may be due to the lack of substitutions at the 1‐position of the indole ring, and/or chlorine/fluorine substitution, which may cause relatively high radical scavenging and anti‐lipid peroxidation activities. However, compound **4j**, which induced ROS generation, and compound **5a**, which reduced ROS generation, do not meet this criterion; this is likely due to the substitution of the CH_3_ group at different positions of the indole ring. Undoubtedly, to avoid speculation and elucidate this SAR, further investigation of the tested compounds at higher concentrations, as well as assessment of the activities of endogenous antioxidant enzymes, such as SOD, catalase, and glutathione peroxidase, which may help reduce ROS levels, and conversely, enzymes that generate ROS are also needed.

**Figure 9:**
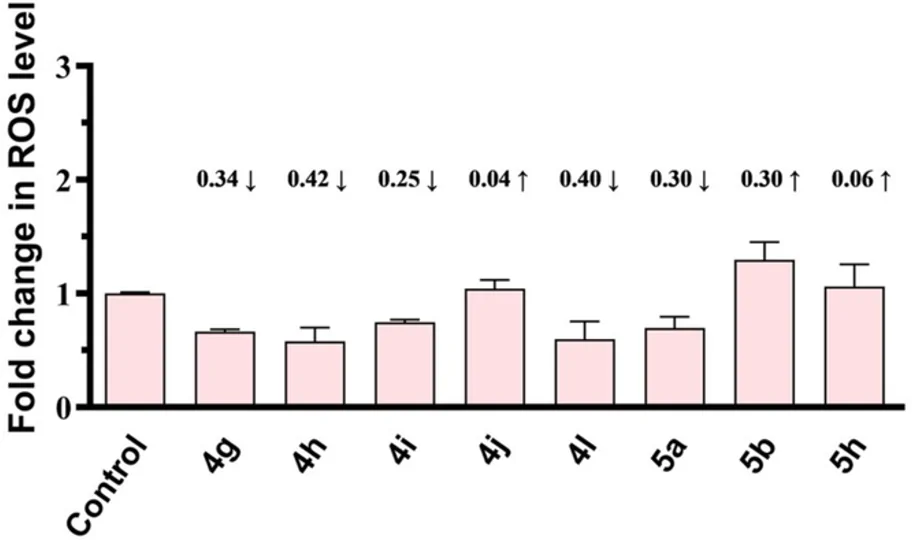
Relative effects of the test compounds on H_2_O_2_–induced ROS generation in HFF‐1 cells.

### 2.5 In Silico Physicochemical Parameters

The physicochemical properties of the compounds **4a–m** and **5a–n** and their compliance with Lipinski's rule of five were evaluated in silico using the QikProp module (Schrödinger LLC, 2015). The molecular weights of all compounds were determined to be within the range of 322.30–462.22 Da, thus remaining below the 500 Da threshold and meeting the related criterion. Compounds with a higher number of hydrogen bond donors and acceptors, which are indicative of their potential to interact with target proteins, are predicted to exhibit stronger binding affinity and consequently higher biological activity. The calculated logP values, reflecting lipophilicity and expected to be < 5, ranged between 2.52 and 4.20, confirming compliance with the rule. Since none of the compounds violated Lipinski's rules (RO5), all derivatives were considered to possess drug‐like properties.

Additional pharmacokinetic parameters, including water solubility (logS), blood–brain barrier partition coefficient (logBB), human serum albumin binding (logKhsa), and predicted human oral absorption (HOA), were also assessed in silico. All evaluated compounds satisfied the recommended thresholds. Oral absorption in humans was defined as > 80% for high oral activity and < 25% for poor oral activity. Predicted HOA was consistently > 87.74% for all derivatives except compound **5h**, which displayed an absorption rate of 75.15%, indicating moderate oral availability. The calculated logS values, indicative of aqueous solubility and expected to fall within the range of −6.5 to 0.5, were determined as −3.70 to −5.32 for all compounds. The logBB values, which estimate the ability of molecules to penetrate the blood–brain barrier and should range between −3.0 and 1.2, were found to be between −0.27 and 1.10. The logKhsa values, representing the binding affinity to human serum albumin and expected to remain within −1.5 to 1.5, ranged from 0.19 to 0.53. Taken together, these findings suggest that all tested spiroindolinone derivatives possess favorable physicochemical and pharmacokinetic properties and meet the essential drug‐likeness criteria (Table S1).

## 3 Conclusion

In this study, novel spiroindolinone 5‐(trifluoromethyl)benzothiazole hybrids (**4a–h**), along with previously reported compounds **4i–m** and **5a–n**, were comprehensively evaluated for their antioxidant potential in order to investigate the contribution of the trifluoromethyl group and determine the SARs of this series of compounds. SAR studies have shown that the presence of an N–H group at the 1‐position of the indole ring is critical for strong antioxidant activity. In contrast, the strategic introduction of halogen substituents emerges as a key structural determinant that enhances overall antioxidant efficacy. Among the radical scavenging analyses, compound **4l** with fluorine substitution at the 5‐position of the indole ring was identified as the most potent antioxidant. While other substituents in the indole ring caused an increase in activity in the order H > 5‐CH_3_ > 5,7‐diCl > 5‐Cl,7‐CH_3_, it was determined that the presence of methyl at the 1‐position and halogen and methyl substitutions at the 7‐position generally decreased the radical scavenging capacities. Based on the TEAC and FRAP results, most compounds exhibited higher activity than the reference antioxidant BHT. Indole N–H–containing compounds (series **4**) showed significantly enhanced radical scavenging and ferric‐reducing abilities compared to the *N*‐methylated series. Among the series, the 5‐chloro‐7‐methyl‐substituted compound **4g** and the 5,7‐dichloro‐substituted compound **4h** displayed the strongest activities in the TEAC and FRAP assays, respectively. However, in lipid peroxidation inhibition assays, while 1‐methyl substitution resulted in significant inhibitory effect losses, 7‐chlorine, 7‐bromine, and 5,7‐dichlorine substituted compound**s** showed higher inhibitory effects than tocopherol. It has been determined that the most potent inhibitors of ROS formation are unsubstituted compounds at the 1‐position of the indole ring, especially 5,7‐dichloro and 5‐fluoro substituted compounds. The results obtained reveal that spirooxindoles 5‐(trifluoromethyl)benzothiazoline hybrids identified to have in silico drug‐like properties are promising lead compounds with their strong antioxidant capacities, lipid peroxidation inhibitory effects, and low cytotoxicity.

It is widely acknowledged that the relationship between spirooxindole structures and their antioxidant activity is intricate, involving multiple factors rather than a single mechanism. The antioxidant properties of spirooxindoles arise from a combination of factors, including their ability to scavenge free radicals, chelate metals, and suppress lipid peroxidation. Additionally, they can modulate cellular antioxidant pathways by activating endogenous defenses via transcription factors such as Nrf2 and by inhibiting ROS‐producing enzymes. The data obtained here highlight the antioxidant activity of the tested compounds for the first three mechanisms in vitro, with some showing dual effects. Overall results indicate that spirooxindoles containing the benzothiazoline moiety show considerable potential for antioxidant activity and warrant further detailed studies, particularly in cellular systems and in vivo.

## 4 Experimental

### 4.1 Chemistry

#### 4.1.1 General Remarks

The chemicals and cell culture reagents used in this study were obtained from commercial sources as follows: L‐α‐phosphatidylcholine (PC) from soybean (Cat No. P5638), α‐tocopherol (Cat No. T3251), MDA (1,1,3,3‐Tetramethoxypropane/Malonaldehyde bis(dimethyl acetal)) (Cat No. 108383), MTT (Cat No. 475989), and 2′,7′‐dichlorodihydrofluorescein diacetate (Cat No. D6883) were purchased from Sigma. Sodium dihydrogen phosphate monohydrate (NaH_2_PO_4_·2H_2_O, Cat No. 106346), disodium hydrogen phosphate dihydrate (Na_2_HPO_4_·2H_2_O, Cat No. 106580), FeCl_2_ (Cat No. 103861), trichloroacetic acid (TCA, Cat No. 100807), HCl (Cat No. 100314), dimethyl sulfoxide (DMSO, Cat No. 102952), and H_2_O_2_ (Cat No. 107209) were procured from Merck. Thiobarbituric acid (TBA, Cat No. T5500) was obtained from Fluka. For cell culture assays, fetal bovine serum (FBS, Cat No. 10500064), non‐essential amino acids (NEAA, Cat No. 11140035), Dulbecco's modified eagle's medium (DMEM, Cat No. 41966029), antibiotic‐antimycotic solution (100X, Cat No. 15240062), sodium pyruvate (Cat No. 11360039), GlutaMAX (Cat No. 35050038), phosphate‐buffered saline (PBS, Cat No. 14190094), and Hanks' Balanced Salt Solution (HBSS, Cat No. 14175095) were supplied by Thermo Fisher Scientific. All chemicals, reagents, and solvents used in the synthesis were purchased from Sigma‐Aldrich, with the exception of 7‐methyl‐1H‐indole‐2,3‐dione (Cat. No. AB243662), which was obtained from Abcr.

IR spectra were determined on KBr discs using a Shimadzu IR Affinity^−1^ FTIR spectrophotometer. Elemental analyses were conducted on a Thermo Finnigan Flash EA 1112 elemental analyzer. ^1^H NMR, ^13^C NMR‐APT, and HMBC‐2D spectra were obtained on Bruker Avance III HD (500 or 600 MHz) and Varian Mercury (Agilent) (400 MHz). The melting points were determined on Büchi Melting Point B‐540 and Büchi Melting Point M‐560 instruments. The mass spectra were confirmed on Agilent Infinity 1260 II LC‐MS. The mass spectral analysis of compounds was carried out using the ESI technique in positive or negative mode. The samples were prepared by diluting the isolated compounds in methanol to a final concentration of 10 µg/mL. The chromatographic purity of the compounds was determined using HPLC‐UV and was always at least 95%; chromatograms are available from the Supporting Information S1.

Thin‐layer chromatography was employed to monitor the reaction progress during synthesis and assess the final compounds' purity. Samples taken from the reaction mixture and the isolated products were analyzed on aluminum plates coated with 0.25 mm thick silica gel HF_254_ as the stationary phase, using an ethyl acetate–cyclohexane (1:1 or 1:1.5) solvent system as the mobile phase. The spots were visualized under UV light at 254 nm.

#### 4.1.2 Synthesis of 5‐(Trifluoromethyl)‐3H‐Spiro[1,3‐Benzothiazole‐2,3′‐Indol]−2′(1′H)‐Ones (**4a–h**)

Compounds **4i–m** and **5a–n** were synthesized according to the cited literature [24, 25]. To a solution of 1*H*‐indole‐2,3‐diones **1a–h** (3.5 mmol) in absolute ethanol (15 mL) was added 2‐amino‐4‐(trifluoromethyl)benzenethiol **3** (3.5 mmol). The mixture was refluxed in a water bath for 3 h. The product formed was separated by filtration and purified by crystallization from an ethanol‐water mixture [22].

5′‐Bromo‐5‐(trifluoromethyl)‐3*H*‐spiro[1,3‐benzothiazole‐2,3′‐indol]‐2′(1′*H*)‐one (**4a**): Orange powder (yield 98%); molecular mass 401.20 g/mol; melting point 217°C–219°C; IR (KBr) cm^−1^: 3290 (N─H), 3088 (aromatic C─H), 1712 (C═O), 1618, 1465, 1452 (C═C); ^1^H‐NMR (DMSO‐*d*_*6*_, 500 MHz) *δ* (ppm): 6.74 (1H, d, *J* = 2.0 Hz, b.t.C_4_─H), 6.84 (1H, d, *J* = 8.4 Hz, ind. C_7_─H), 6.95 (1H, d, *J* = 8.0 Hz, b.t. C_6_─H), 7.28 (1H, d, *J* = 8.0 Hz, b.t. C_7_─H), 7.50 (1H, dd, *J* = 8.4; 2.0 Hz, ind. C_6_─H), 7.71 (1H, d, *J* = 2.0 Hz, ind. C_4_─H), 7.75 (1H, s, b.t. N─H), 10.63 (1H, s, ind. N─H); ^13^C NMR (APT) (125 MHz, DMSO‐*d6*) *δ* (ppm): 74.69 (spiro C), 103.57 (q, *J* = 3.8 Hz, b.t. C_4_), 112.38 (ind. C_7_), 114.08 (ind. C_5_), 115.47 (q, *J* = 3.9 Hz, b.t. C_6_), 121.45 (b.t. C_7_), 124.47 (q, *J* = 274.3 Hz, CF_3_), 128.41 (ind. C_4_), 126.72 (b.t. C_5_, q, *J* = 31.0 Hz), 131.67 (b.t. C_7a_), 133.56 (ind. C_6_), 129.54 (ind. C_3a_), 140.64 (ind. C_7a_), 147.58 (b.t. C_3a_), 175.41 (ind. C_2_); LC‐MS (ESI+): 415.0, 417.0 ([M + 2Li]^+^; 10,9), 400.9, 402.9 ([M + H]^+^; 100, 82); 335.1, 337.2, 339.1 (22, 59, 18), 325.1 (35), 319.2, 321.1 (37, 22), 281.2 (55); Analyses (%) cald for C_15_H_8_BrF_3_N_2_OS C, 44.91; H, 2.01; N, 6.98; S, 7.99. Found: C, 44.88; H, 2.03; N, 7.04; S, 7.89. HPLC purity: 98.2%.

5′‐Iodo‐5‐(trifluoromethyl)‐3*H*‐spiro[1,3‐benzothiazole‐2,3′‐indol]‐2′(1′*H*)‐one (**4b**): Orange powder (yield 84%); molecular mass 448.20 g/mol; melting point 221°C–222°C; IR (KBr) cm^−1^: 3290 (N─H), 3090 (aromatic C─H), 1712 (C═O), 1614, 1464, 1454 (C═C); ^1^H‐NMR (DMSO‐*d*_*6*_, 500 MHz) *δ* (ppm): 6.72 (1H, d, *J* = 8.0 Hz, ind. C_7_─H), 6.73 (1H, d, *J* = 1.6 Hz, b.t. C_4_─H), 6.95 (1H, d, *J* = 8.0 Hz, b.t. C_6_─H), 7.27 (1H, d, *J* = 8.0 Hz, b.t. C_7_─H), 7.65 (1H, dd, *J* = 8.4, 1.6 Hz, ind. C_6_─H), 7.74 (1H, s, b.t. N─H), 7.82 (1H, d, *J* = 1.6 Hz, ind. C_4_─H), 10.60 (1H, s, ind. N─H); ^13^C NMR (APT) (125 MHz, DMSO‐*d6*) *δ* (ppm): 74.69 (spiro C), 85.19 (ind. C_5_), 103.53 (q, *J* = 3.2 Hz, b.t. C_4_), 112.79 (ind. C_7_), 115.44 (q, *J* = 4.5 Hz, b.t. C_6_), 121.43 (b.t. C_7_), 124.48 (q, *J* = 271.7 Hz, CF_3_), 126.70 (q, *J* = 31.0 Hz, b.t. C_5_), 129.55 (ind. C_3a_), 131.86 (b.t. C_7a_), 133.90 (ind. C_4_) 139.30 (ind. C_6_), 141.08 (ind. C_7a_), 147.58 (b.t. C_3a_), 175.16 (ind. C_2_); LC‐MS (ESI+): 470.9 ([M + Na]^+^, 18), 462.9 ([M + 2Li]^+^, 59), 448.9 ([M + H]^+^, 100), 446.9 (75), 337.0 (35), 325.2 (28); Analyses (%) cald for C_15_H_8_F_3_IN_2_OS C, 40.20; H, 1.80; N, 6.25; S, 7.15. Found: C, 40.17; H, 1.84; N, 6.44; S, 7.11. HPLC purity: 99.6%.

7′‐Methyl‐5‐(trifluoromethyl)‐3*H*‐spiro[1,3‐benzothiazole‐2,3′‐indol]‐2′(1′*H*)‐one (**4c**): Orange powder (yield 70%); molecular mass 336.33 g/mol; melting point 246°C–247°C; IR (KBr) cm^−1^: 3288, 3361 (N─H), 3100 (aromatic C─H), 1701 (C═O), 1627, 1489, 1446 (C═C); ^1^H‐NMR (DMSO‐*d*_*6*_, 500 MHz) *δ* (ppm): 3.32 (3H, s, CH_3_), 6.73 (1H, d, *J* = 2.0 Hz, b.t. C_4_─H), 6.93 (1H, d, *J* = 8.0 Hz, b.t. C_6_─H), 6.99 (1H, d, *J* = 7.6 Hz, ind. C_5_─H); 7.14 (1H, d, *J* = 7.6 Hz, ind. C_6_─H), 7.26 (1H, d, *J* = 7.6 Hz, b.t. C_7_─H), 7.41 (1H, d, *J* = 7.2 Hz, ind. C_4_─H), 7.60 (1H, s, b.t. N─H), 10.48 (1H, s, ind. N─H); ^13^C NMR (APT) (125 MHz, DMSO‐*d6*) *δ* (ppm): 16.00 (CH_3_), 74.60 (spiro C), 103.33 (q, *J* = 3.8 Hz, b.t. C_4_), 115.24 (q, *J* = 3.3 Hz, b.t. C_6_), 119.74 (ind. C_7_), 121.37 (b.t. C_7_), 122.62 (ind. C_4_), 123.07 (ind. C_5_), 124.52 (q, *J* = 271.7 Hz, CF_3_), 126.60 (q, *J* = 30.9 Hz, b.t. C_5_), 128.64 (ind. C_3a_), 129.87 (b.t. C_7a_), 132.00 (ind. C_6_); 139.92 (ind. C_7a_), 147.96 (b.t. C_3a_), 176.28 (ind. C_2_); LC‐MS (ESI+): 359.0 ([M + Na]^+^, 22), 351.0 ([M + 2Li]^+^, 53), 337.0 ([M + H]^+^, 100), 338.0 (18), 335.0 (20), 325.1 (8); Analyses (%) cald for C_16_H_11_F_3_N_2_OS C, 57.14; H, 3.30; N, 8.33; S, 9.53. Found: C, 57.21; H, 3.28; N, 8.46; S, 9.42. HPLC purity: 100.0%.

7′‐Fluoro‐5‐(trifluoromethyl)‐3*H*‐spiro[1,3‐benzothiazole‐2,3′‐indol]‐2′(1′*H*)‐one (**4d**): Orange powder (yield 61%); molecular mass 344.80 g/mol; melting point 158°C–161°C; IR (KBr) cm^−1^: 3196, 3284 (N─H), 3034 (aromatic C─H), 1730 (C═O), 1643, 1492, 1450 (C═C); ^1^H‐NMR (DMSO‐*d*_*6*_, 500 MHz) *δ* (ppm): 6.74 (1H, d, *J* = 1.6 Hz, b.t. C_4_─H), 6.95 (1H, d, *J* = 7.6 Hz, b.t. C_6_─H), 7.08 (1H, td, *J* = 8.0, 7.6, 4.8 Hz, ind. C_5_─H), 7.23‐7.29 (1H, m, ind. C_6_─H), 7.28 (1H, d, *J* = 8.0 Hz, b.t. C_7_─H), 7.44 (1H, d, *J* = 7.60 Hz, ind. C_4_─H), 7.76 (1H, s, b.t. N─H), 11.0 (1H, s, ind. N─H); ^13^C NMR (APT) (125 MHz, DMSO‐*d6*) *δ* (ppm): 74.82 (spiro C), 103.49 (q, J = 3.8 Hz, b.t. C4), 115.44 (q, J = 4.5 Hz, b.t. C6), 117.71 (d, J = 17.0 Hz, ind. C_6_), 121.47 (b.t. C_7_), 121.75 (d, J = 3.2 Hz, ind. C_4_), 123.54 (d, J = 5.8, ind. C_5_), 124.48 (q, J = 271.6 Hz, CF_3_), 126.74 (q, J = 31.6 Hz, b.t. C_5_), 129.50 (b.t. C_7a_), 132.04 (d, J = 3.2 Hz, ind. C_3a_), 128.50 (d, J = 13.5 Hz, ind. C_7a_), 146.32 (d, J = 243.8 Hz, ind. C_7_), 147.63 (b.t. C_3a_), 175.69 (ind. C_2_); LC‐MS (ESI+): 363.0 ([M + Na]^+^, 28), 355.0 ([M + 2Li]^+^, 18), 341.0 ([M + H]^+^, 100), 339 (63), 325.1 (6), 226.1 (10); Analyses (%) cald for C_15_H_8_F_4_N_2_OS.^1^/_4_H_2_O C, 52.25; H, 2.48; N, 8.12; S, 9.30. Found: C, 52.29; H, 2.48; N, 8.38; S, 9.30. HPLC purity: 98.1%.

7′‐Chloro‐5‐(trifluoromethyl)‐3*H*‐spiro[1,3‐benzothiazole‐2,3′‐indol]‐2′(1′*H*)‐one (**4e**): Yellow powder (yield 68%); molecular mass 365.76 g/mol; melting point 201°C–204°C; IR (KBr) cm^−1^: 3300, 3369 (N─H), 3068 (aromatic C─H), 1708 (C═O), 1622, 1454, 1446 (C═C); ^1^H‐NMR (DMSO‐*d*_*6*_, 500 MHz) *δ* (ppm): 6.75 (1H, d, *J* = 1.6 Hz, b.t. C_4_─H), 6.96 (1H, dd, *J* = 7.6 Hz, b.t. C_6_─H), 7.10 (1H, dd, *J* = 8.0, 7.6 Hz, ind.C_5_─H), 7.28 (1H, d, *J* = 7.6 Hz, b.t. C_7_─H), 7.40 (1H, d, *J* = 8.0 Hz, ind. C_6_─H), 7.56 (1H, d, *J* = 7.6 Hz, ind. C_4_─H), 7.75 (1H, s, b.t. N─H), 10.91 (1H, s, ind. N─H); ^13^C NMR (APT) (125 MHz, DMSO‐*d6*) *δ* (ppm): 75.28 (spiro C), 103.89 (q, *J* = 3.8 Hz, b.t. C_4_), 114.40 (ind. C_7′_), 115.42 (q, *J* = 3.9 Hz, b.t. C_6_), 121.45 (b.t. C_7_), 124.38 (ind. C_4′_), 123.94 (ind. C_5′_), 126.73 (q, *J* = 31.7 Hz, b.t. C_5_), 129.46 (ind. C_3a’_), 130.59 (ind. C_6′_), 131.18 (b.t. C_7a_), 139.03 (ind. C_7a’_), 147.57 (b.t. C_3a_), 175.82 (ind. C_2′_), LC‐MS (ESI+): 379.0, 380,9 ([M + Na]^+^; 43, 14), 371.0, 373.0 ([M + 2Li]^+^;18, 7), 357.0, 358.9 ([M + H]^+^, 100, 28), 355 (75), 336.1 (15), 325.1 (15); Analyses (%) cald for C_15_H_8_ClF_3_N_2_OS.^1^/_2_H_2_O C, 49.26; H, 2.48; N, 7.65; S, 8.76. Found: C, 49.61; H, 2.37; N, 7.81; S, 8.85. HPLC purity: 99.7%.

7′‐Bromo‐5‐(trifluoromethyl)‐3*H*‐spiro[1,3‐benzothiazole‐2,3′‐indol]‐2′(1′*H*)‐on (**4f**): Orange powder (yield 59%); molecular mass 401.20 g/mol; melting point 206°C–207°C; IR (KBr) cm^−1^: 3286, 3354 (N─H), 2916 (aromatic C─H), 1707 (C═O), 1620, 1477, 1450 (C═C); ^1^H‐NMR (DMSO‐*d*_*6*_, 500 MHz) *δ* (ppm): 6.75 (1H, d, *J* = 1.3 Hz, b.t. C_4_─H), 6.96 (1H, d, *J* = 7.7 Hz, b.t. C_6_─H), 7.04 (1H, dd, *J* = 7.7, 7.8 Hz ind. C_5_─H), 7.29 (1H, d, *J* = 7.9 Hz, b.t. C_7_─H), 7.53 (1H, d, *J* = 8.1 Hz, ind. C_6_─H), 7.60 (1H, d, *J* = 71 Hz, ind. C_4_─H), 7.77 (1H, s, b.t. N─H), 10.80 (1H, s, ind. N─H); HMBC‐2D (125 MHz, DMSO‐*d6*) *δ* (ppm): 76.02 (spiro C), 103.06 (ind. C_7_), 103.95 (q, *J* = 3.5 Hz, b.t. C_4_), 115.91 (q, *J* = 4.0 Hz, b.t. C_6_), 121.94 (b.t. C_7_), 124.93 (q, *J* = 271.6 Hz, CF_3_), 124.84 (ind. C_4_), 125.35 (ind. C_5_), 127.21 (q, *J* = 31.4 Hz, b.t. C_5_), 129.93 (b.t. C_7a_), 131.71 (ind. C_3a_), 133.99 (ind. C_6_), 141.19 (ind. C_7a’_), 148.05 (b.t. C_3a_), 176.26 (ind. C_2_); LC‐MS (ESI+): 400.9, 403.0 ([M + H]^+^, 100, 98), 399.9, 401.9 (20, 18); Analyses (%) cald for C_15_H_8_BrF_3_N_2_OS C, 44.91; H, 2.01; N, 6.98; S, 7.99. Found: C, 45.03; H, 2.05; N, 7.25; S, 8.04. HPLC purity: 97.8%.

5′‐Chloro‐7′‐methyl‐5‐(trifluoromethyl)‐3*H*‐spiro[1,3‐benzothiazole‐2,3′‐indol]‐2′(1′*H*)‐on (**4g**): Yellow powder (yield 68%); molecular mass 370.77 g/mol; melting point 254°C–257°C; IR (KBr) cm^−1^: 3309 (N─H), 3074 (aromatic C─H), 1710 (C═O), 1625, 1463, 1415 (C═C); ^1^H‐NMR (DMSO‐*d*_*6*_, 500 MHz) *δ* (ppm):2.21 (3H, s, CH_3_), 6.74 (1H, d, *J* = 2.0 Hz, b.t. C_4_─H), 6.95 (1H, d, *J* = 7.6 Hz, b.t. C_6_─H), 7.24 (1H, d, *J* = 2.4 Hz, ind. C_6_─H), 7.27 (1H, d, *J* = 8.0 Hz, b.t. C_7_─H), 7.42 (1H, d, *J* = 2.0 Hz, ind. C_4_─H), 7.74 (1H, s, b.t. N─H), 10.67 (1H, s, ind. N─H); ^13^C NMR (APT) (125 MHz, DMSO‐*d6*) *δ* (ppm) :16.34 (CH_3_), 75.40 (spiro C), 103.99 (q, *J* = 3.8 Hz, b.t. C_4_), 115.88 (q, *J* = 3.9 Hz, b.t. C_6_), 121.88 (b.t. C_7_), 122.48 (ind. C_7_), 123.28 (ind. C_4_), 124.92 (q, *J* = 271.7 Hz, CF_3_), 126.82 (ind. C_5_), 127.15 (q, *J* = 31.0 Hz, b.t. C_5_), 130.03 (ind. C_3a_), 131.84 (ind. C_6_), 131.11 (b.t. C_7a_), 139.32 (ind. C_7a_), 148.11 (b.t. C_3a_), 176.48 (ind. C_2_); LC‐MS (ESI+): 393.0, 395.0 ([M+Na]^+^; 13, 4), 385.0, 387.1 ([M + 2Li]^+^; 15, 6), 371.0, 373.0 ([M + H]^+^, 88, 25), 369.0 (58); 336.3 (18); 325.2 (20); 310 (32), 282.3, 284.2 (100, 18); Analyses (%) cald for C_16_H_10_ClF_3_N_2_OS C, 51.83; H, 2.72; N, 7.56; S, 8.65. Found: C, 52.04; H, 2.74; N, 7.68; S, 8.52. HPLC purity: 100.0%.

5′,7′‐Dichloro‐5‐(trifluoromethyl)‐3*H*‐spiro[1,3‐benzothiazole‐2,3′‐indol]‐2′(1′*H*)‐one (**4h**): Orange powder (yield 95%); molecular mass 391.19 g/mol; melting point 191°C–194°C; IR (KBr) cm^−1^: 3331 (N─H), 3080 (aromatic C─H), 1718 (C═O), 1620, 1458 (C═C); ^1^H‐NMR (DMSO‐*d*_*6*_, 500 MHz) *δ* (ppm): 6,77 (1H, d, *J* = 1,6 Hz, b.t. C_4_─H); 6,97 (1H, d, *J* = 7,6 Hz, b.t. C_6_─H); 7,30 (1H, d, *J* = 8,0 Hz, b.t. C_7_─H); 7,59 (1H, d, *J* = 2,0 Hz, ind. C_6_─H); 7,60 (1H, d, *J* = 2,0 Hz, ind. C_4_─H); 7,75 (1H, s, b.t. N─H); 11,11 (1H, s, ind. N─H); ^13^C NMR (APT) (125 MHz, DMSO‐*d6*) *δ* (ppm): 75,25 (spiro C); 103,70 (q, *J* = 4,0 Hz b.t. C_4_); 115,20 (ind. C_7_); 115,58 (q, *J* = 3,8 Hz b.t. C_6_); 121,51 (b.t. C_7_); 124,47 (q, *J* = 271,7 Hz, CF_3_); 124,56 (ind. C_4_); 126,81 (q, *J* = 31,7 Hz b.t. C_5_); 127,09 (C_5_); 129,29 (b.t. C_7a_); 130,01 (ind. C_6_); 132,81 (ind. C_3a_); 138,27 (ind. C_7a_); 147,32 (b.t. C_3a_); 175,66 (ind. C_2_); LC‐MS (ESI−): 388,9; 390,9; 392,9 ([M‐H]^−^, 100, 78, 13), 390,0; 391,9; 393,9 ([M]^+^, 18, 13, 4); Analyses (%) cald for C_15_H_7_Cl_2_F_3_N_2_OS C, 46.06; H, 1.80; N, 7.16; S, 8.20. Found: C, 46.10; H, 1.85; N, 7.23; S, 8.20. HPLC purity: 100.0%.

### 4.2 X‐Ray Crystal Structure Determination and Refinement

Single‐crystal X‐ray diffraction data for compound **5h** were collected on a STOE IPDS II image plate diffractometer at 293 K. Graphite‐monochromated MoKα radiation (*λ* = 0.71073 Å), and the scan technique were used. The structure of **5h** was solved by direct methods using SHELXS‐97 [36] and refined by the full‐matrix least‐squares on F 2 using SHELXL‐2014 [37], as implemented in the WinGX program suite [38]. Non‐hydrogen atoms were refined with anisotropic displacement parameters. Data collection and cell refinement for compound **5h** were carried out using Stoe X‐AREA, while data reduction was performed with Stoe X‐RED [39]. The general‐purpose crystallographic program PLATON [40] was employed for structure analysis and presentation of the results. The dihedral angles were calculated using the PARST95 program [34].

### 4.3 Determination of Antioxidant Activity

#### 4.3.1 Determination of DPPH Radical Scavenging Effect

The DPPH radical scavenging activity of the compounds was determined according to the method developed by Brand‐Williams et al. [41]. To 0.1 mL of indole derivative solution in DMSO (0.625–10 mM), or BHT (0.625–10 mM) and α‐tocopherol (0.08–0.625 mM), 3.9 mL of DPPH‐solution in methanol (6 × 10^−5^ M) was added, and the mixture was kept in the dark for 30 min. Absorbances were read against methanol in a spectrophotometer at 517 nm. Positive and negative controls were run in parallel, with the positive control using standard (α‐tocopherol and BHT) in place of the compounds under study and DMSO as the negative control. The experiment was repeated three times and averaged. DPPH radical scavenging activity was calculated according to the following equation:

$$\mathrm{DPPH}\;\mathrm{radical}\;\mathrm{scavenging}\;\mathrm{activity}\;(\%)=\left(1-\frac{\mathrm{Absorbance}\;\mathrm{of}\;\mathrm{sample}\;\mathrm{at}\;517\;\mathrm{nm}}{\mathrm{Absorbance}\;\mathrm{of}\;\mathrm{control}\;\mathrm{at}\;517\;\mathrm{nm}}\right)\times 100.$$

#### 4.3.2 Determination of Superoxide Radical Scavenging Effect

The superoxide radical scavenging effect of the compounds was determined according to the method developed by Aruoma et al. [42]. The experiment is based on reading the absorbance at 560 nm of blue‐purple colored formazones formed as a result of the reduction of nitroblue tetrazolium (NBT) by superoxide radicals produced by the xanthine/xanthine oxidase system. The experiment was carried out in a 3 mL reaction medium. 100 μL indole derivative (0.625–10 mM) or BHT (0.625–10 mM) and, α‐tocopherol (0.16–2.5 mM), 100 μL 30 mM ethylene diamine tetra acetic acid (1 mM), 10 μL 30 mM xanthine dissolved in 50 mM KOH (0.1 mM), 100 μL 3 mM NBT (0.1 mM) and 2.59 mL 57.5 mM KH_2_PO_4_–KOH, pH 7.4 (50 mM) were mixed and the reaction was initiated by the addition of 100 μL 1U xanthine oxidase. After 5 min at 25°C, absorbances were read against the blind at 560 nm. The experiment was repeated three times and averaged and the superoxide radical scavenging activity was calculated according to the following equation:

$$\mathrm{Superoxide}\;\mathrm{radical}\;\mathrm{scavenging}\;(\%)=\left(1-\frac{\mathrm{Absorbance}\;\mathrm{of}\;\mathrm{indole}\;\mathrm{derivative}\;\mathrm{at}\;560\;\mathrm{nm}}{\mathrm{Absorbance}\;\mathrm{of}\;\mathrm{control}\;\mathrm{at}\;560\;\mathrm{nm}}\right)\times 100.$$

#### 4.3.3 TEAC Assay

The total radical antioxidant potential of the indole derivatives was measured using the Trolox equivalent antioxidant coefficient (TEAC) assay as described by Re et al. [43] with minor modifications. The TEAC value is based on the ability of the antioxidant molecules to quench the long‐lived ABTS^•+^, a blue‐green chromophore with characteristic absorption at 734 nm, compared with that of Trolox, a water‐soluble vitamin E analog. ABTS was dissolved in water to a 7 mM concentration. ABTS radical cation was produced by reacting ABTS^•+^ stock solution with 2.45 mM potassium persulfate (final concentration) and allowing the mixture to stand in the dark at room temperature for 12–16 h before use. At the beginning of the analysis day, an ABTS^•+^ working solution was obtained by the dilution in 96% ethanol of the stock solution to an absorbance of 0.70 (± 0.02) at 734 nm. After the addition of 990 μL of ABTS^•+^ solution to 10 μL of indole derivatives (0.625–10 mM) in DMSO, (0.625–10 mM), α‐tocopherol (0.16–2.5 mM) or trolox standards (1.5, 1, 0.5, 0.5, 0.25, and 0.125 mM), the decrease in absorbance at 734 nm was monitored exactly 6 min after the initial mixing. Appropriate DMSO or ethanol blanks were run in each assay. All determinations were carried out in triplicate. The ability to scavenge ABTS^•+^ radical was calculated by the following equation:

$$\mathrm{ABTS}\;\mathrm{radical}\;\mathrm{scavenging}\;\mathrm{activity}\;\left(\%\right)=\left(1-\frac{\mathrm{Absorbance}\;\mathrm{of}\;\mathrm{indole}\;\mathrm{derivative}\;\mathrm{at}\;734\;\mathrm{nm}}{\mathrm{Absorbance}\;\mathrm{of}\;\mathrm{control}\;\mathrm{at}\;734\;\mathrm{nm}}\right)\times 100.$$

The TEAC values were calculated by using a regression equation between the Trolox concentrations and the percentage of inhibition of absorbance at 734 nm at 6 min of incubation and were expressed as mM TEAC.

#### 4.3.4 Ferric Reducing Antioxidant Power (FRAP) Assay

The FRAP assay was carried out according to the procedure of Benzie and Strain [44]. The FRAP reagent contained 2.5 mL of 10 mM TPTZ solution in 40 mM HCl, plus 2.5 mL of 20 mM FeCl_3_.6H_2_O and 25 mL of 0.3 M acetate buffer, pH 3.6. FRAP reagent (900 μL), prepared freshly and incubated at 37°C, was mixed with 90 μL of DMSO and 30 μL of the tested sample in DMSO or DMSO (for the reagent blank). The increase in absorbance at 593 nm was measured at 4. min. The standard curve was constructed using iron sulfate heptahydrate solution (125–2000 μM), and the results were expressed as mM/l Fe^2+^. All the measurements were taken in triplicate, and the mean values were calculated.

#### 4.3.5 Determination of Lipid Peroxidation Inhibitory Activity

To determine the capacity of indole derivatives to inhibit lipid peroxidation, the TBARs method recommended by Patro et al. [45] and modified by Hamad et al. [46] was applied. An artificial lipid system consisting of liposomes prepared from soy PC was used. Liposome suspension was prepared by sonication of a mixture containing 300 mg PC in 30 mL of Na‐phosphate buffer (10 mM, pH 7.4) for 2 h on ice. Liposome suspension (250 μL), 5 mM of indole derivatives or DMSO as a control (450 μL), FeCl_2_, H_2_O_2_, and ascorbic acid, each in a final concentration of 125 μM (to a final volume of 1 mL), were mixed and incubated at 30°C for 4 h. α‐Tocopherol was used as a reference antioxidant. Following 4 h of incubation, malondialdehyde (MDA), one of the end products of lipid peroxidation, was detected by using the TBA reagent, containing 0.375% TBA, 15% TCA, and 0.25 M HCl. A 500 μL TBA reagent was added to a 250 μL assay mixture. MDA solutions at various concentrations were also reacted with the TBA reagent to generate a standard MDA curve. These mixtures were kept at 100°C for 15 min. After cooling to room temperature, pink colored TBA‐reactive species (TBARs), as adducts of MDA with 2‐TBA, were obtained by centrifugation (3000×*g*, 5 min) as a supernatant, and their absorbance at 532 nm was recorded spectrophotometrically. MDA levels in the assay mixtures were calculated using the MDA standard curve. Lipid peroxidation inhibition (%) was calculated using the following formula.

$$\mathrm{Lipid}\;\mathrm{peroxidation}\;\mathrm{inhibition}\;(\%)=\left(1-\frac{\mathrm{MDA}\;\mathrm{level}\;\mathrm{of}\;\mathrm{sample}\;}{\mathrm{MDA}\;\mathrm{level}\;\mathrm{of}\;\mathrm{control}\;}\right)\times 100.$$

The quantitative data were presented as mean ± SD based on at least three independent experiments. Statistical evaluation was performed using GraphPad Prism 8 software with one‐way analysis of variance (ANOVA) followed by Dunnett's post hoc test for multiple comparisons. The criterion for statistical significance was *p* < 0.05.

### 4.4 Cell Culture Studies

#### 4.4.1 Cytotoxicity Test

Human foreskin fibroblast cell line (HFF‐1, ATCC, SCRC‐1041) was used in the study. The cells were cultured in 25 cm^2^ culture dishes using heat inactivated 10% FBS, 1% NEAA, DMEM containing 1% antibiotic‐antimycotic solution, 1% sodium pyruvate (100 mM), 1% GlutaMAX (200 mM), and 4.5 g/L D‐glucose [47] in an incubator (37°C, 5% CO_2_). The cytotoxic effects of 8 compounds (**4g**, **4h**, **4i**, **4j**, **4l**, **5a**, **5b**, and **5h**), which were determined to have antioxidant activity in at least two in vitro tests, were detected by using the MTT method proposed by Mosmann [48] and modified by Uçar et al [49]. For this purpose, cells were seeded into 96‐well culture dishes and incubated for 24 h at 37°C and in an incubator containing 5% CO_2_. At the end of this period, the medium on the cells that had adhered to the culture dish was removed, and medium containing different concentrations of compounds (only medium for the control group) was added to the experimental group cells and kept in the oven for 48 h. At the end of this period, the medium on the cells was removed, and 30 µL of MTT stock solution (5 mg/mL, in PBS) was added to each well. Followed by 4 h of incubation, 150 µL of DMSO was added to each well, and the mixture was shaken until homogeneous. Finally, the absorbance of the colored products was measured in a microplate reader (Multiplate Reader UV/Vis Reader, BioTek), and the % viability value was calculated using the following formula:

$$\mathrm{Viability}\;(\%)=100\times \frac{\mathrm{Absorbance}\;\mathrm{in}\;\mathrm{experimental}\;\mathrm{group}\;\mathrm{at}\;540\;\mathrm{nm}}{\mathrm{Absorbance}\;\mathrm{in}\;\mathrm{control}\;\mathrm{group}\;\mathrm{at}\;540\;\mathrm{nm}}.$$

The quantitative data were presented as mean ± SD based on at least three independent experiments. For statistical analysis, GraphPad Prism 8.0 was used, and nonlinear regression was applied to the sigmoidal dose‐response curve to calculate IC_50_ values.

#### 4.4.2 Determination of Intracellular ROS

HFF‐1 cells were grown in 96‐well culture plates (black ones for fluorescence measurement) for 24 h before measurement. Oxidative stress was induced by exposing the cells to 200 µM H_2_O_2_ for 2 h. Based on the cytotoxicity results, a concentration of 15 μM was chosen, as it did not reduce cell viability below 100% for any of the tested compounds. This concentration was then used to evaluate the effects of the compounds on intracellular ROS generation. The experimental group was treated with 15 µM of each test compound for 48 h. The control group was treated with DMSO (the solvent used to dissolve the samples) instead of the samples, following H_2_O_2_ exposure. Intracellular ROS were determined spectrofluorometrically using 2′,7′‐dichlorodihydrofluorescein diacetate (H_2_DCFDA). For this purpose, the liquid media in the control and experimental groups were gently removed, and the cells were washed with PBS. Then, 10 μM H_2_DCFDA in a balanced salt solution (HBSS) was applied to the cells at a concentration of 200 μL in each well, and the culture dishes were incubated at 37°C in an oven containing 5% CO_2_ for 30 min. After removing the supernatant, 100 μL HBSS was added to each well. Kinetic measurements were taken in a spectrofluorometer (FLx800 Fluorescence Microplate Reader, BioTek) at 495 nm excitation and 525 nm emission wavelengths for 2 h at 37°C. ROS levels were calculated by normalizing fluorescence measurements to the cell number in each well. The results were evaluated as a fold change in ROS level in the experimental groups compared to the control.

The quantitative data were presented as mean ± SD based on at least three independent experiments. Statistical evaluation was performed using GraphPad Prism 8 software with one‐way ANOVA followed by Dunnett's post hoc test for multiple comparisons. The criterion for statistical significance was *p* < 0.05.

### 4.5 In Silico Determination of Physicochemical Parameters

Lipinski's rule of five (RO5), defined in 1977 by Lipinski et al. [50], is based on their study of small and lipophilic drug molecules. This study encompasses various pharmacokinetic properties of drug candidate molecules in the body, including absorption, distribution, metabolism, and excretion. It is a set of rules commonly used to assess oral bioavailability and drug‐like properties. This set of rules does not define the pharmacologic activity of drug candidate molecules. Although it has lost some of its principles and value with the new rules developed today, it remains important as the basic criterion in the design and development of new drug molecules targeted for oral use.

Consisting of four rules, RO5 allows for a maximum of one rule not to be followed. RO5 states that a candidate molecule must meet the following criteria to be orally available and have drug‐like properties:

– Hydrogen bond donor group should not be more than 5.
– Hydrogen bond acceptor group should not be more than 10.
– Molecular weight should be less than 500 daltons.
– Octanol/water distribution coefficient (logP) must be less than 5.
– Must not violate more than one rule.

Compliance with Lipinski's RO5 and some pharmacokinetic parameters of synthesized compounds **4a–m** and **5a–n** were analyzed in silico using the Qikprop module (Schrödinger LLC 2015).

## Conflicts of Interest

The authors declare no conflicts of interest.

## Supporting information


Supporting File 1

Supporting File 2

## Data Availability

The data that support the findings of this study are available from the corresponding author upon reasonable request.
