# Supplementary material for: Spirooxindole 5‐(Trifluoromethyl)benzothiazole Hybrids: Synthesis, Crystal Structure, and Antioxidant Activity
Source: Arch Pharm (Weinheim). 2026 Sep 28;359(9):e70343. doi: 10.1002/ardp.70343 (PMC13617587; doi:10.1002/ardp.70343)
Supplement: Supplementary file 1 [file ARDP-359-e70343-s002.docx]

**Spirooxindole 5-(trifluoromethyl)benzothiazole hybrids: Synthesis, crystal structure and antioxidant activity**

**Supplementary Material**

**Figures**

[Figure S- 1: IR spectrum of **4a**	10](#_Toc230597914)

[Figure S- 2: ^1^H-NMR spectrum of compound **4a** ( δ 0-14 ppm)	11](#_Toc230597915)

[Figure S- 3: ^1^H-NMR spectrum of compound **4a** ( δ 6.6-8.0 ppm)	11](#_Toc230597916)

[Figure S- 4: ^13^C-NMR APT spectrum of compound **4a** ( δ 0-200 ppm)	12](#_Toc230597917)

[Figure S- 5: LC-MS spectrum of compound **4a**	12](#_Toc230597918)

[Figure S- 6: HPLC chromatogram of compound **4a**	13](#_Toc230597919)

[Figure S- 7: IR spectrum of compound **4b**	13](#_Toc230597920)

[Figure S- 8: ^1^H-NMR spectrum of compound **4b** ( δ 0-14 ppm)	14](#_Toc230597921)

[Figure S- 9: ^1^H-NMR spectrum of compound **4b** ( δ 6.6-8.0 ppm)	14](#_Toc230597922)

[Figure S- 10: ^13^C-NMR APT spectrum of compound **4b** ( δ 0-200 ppm)	15](#_Toc230597923)

[Figure S- 11: LC-MS spectrum of compound **4b**	15](#_Toc230597924)

[Figure S- 12: HPLC chromatogram of compound **4b**	16](#_Toc230597925)

[Figure S- 13: IR spectrum of compound **4c**	16](#_Toc230597926)

[Figure S- 14: ^1^H-NMR spectrum of compound **4c** ( δ 0-14 ppm)	17](#_Toc230597927)

[Figure S- 15: ^1^H-NMR spectrum of compound **4c** ( δ 6.4-8.2 ppm)	17](#_Toc230597928)

[Figure S- 16: ^13^C-NMR APT spectrum of compound **4c** ( δ 0-200 ppm)	18](#_Toc230597929)

[Figure S- 17: LC-MS spectrum of compound **4c**	18](#_Toc230597930)

[Figure S- 18: HPLC chromatogram of compound **4c**	19](#_Toc230597931)

[Figure S- 19: IR spectrum of compound **4d**	19](#_Toc230597932)

[Figure S- 20: ^1^H-NMR spectrum of compound **4d** ( δ 0-14 ppm)	20](#_Toc230597933)

[Figure S- 21: ^1^H-NMR spectrum of compound **4d** ( δ 6.6-7.6 ppm)	20](#_Toc230597934)

[Figure S- 22: ^13^C-NMR APT spectrum of compound **4d** ( δ 0-180 ppm)	21](#_Toc230597935)

[Figure S- 23: LC-MS spectrum of compound **4d**	21](#_Toc230597936)

[Figure S- 24: HPLC chromatogram of compound **4d**	22](#_Toc230597937)

[Figure S- 25: R spectrum of compound **4e**	22](#_Toc230597938)

[Figure S- 26: ^1^H-NMR spectrum of compound **4e** ( δ 0-14 ppm)	23](#_Toc230597939)

[Figure S- 27: ^1^H-NMR spectrum of compound **4e** ( δ 6.6-8.0 ppm)	23](#_Toc230597940)

[Figure S- 28: ^13^C-NMR APT spectrum of compound **4e** ( δ 0-190 ppm)	24](#_Toc230597941)

[Figure S- 29: ^13^C-NMR APT spectrum of compound **4e** ( δ 0-190 ppm)	24](#_Toc230597942)

[Figure S- 30: HPLC chromatogram of compound **4e**	25](#_Toc230597943)

[Figure S- 31: IR spectrum of compound **4f**	25](#_Toc230597944)

[Figure S- 32: ^1^H-NMR spectrum of compound **4f** ( δ 0-12.0 ppm)	26](#_Toc230597945)

[Figure S- 33: ^1^H-NMR spectrum of compound **4f** ( δ 6.6-8.0 ppm)	26](#_Toc230597946)

[Figure S- 34: ^13^C-NMR APT spectrum of compound **4f** ( δ 126-136 ppm)	27](#_Toc230597947)

[Figure S- 35: ^13^C-NMR APT spectrum of compound **4f** ( spiro C signal)	27](#_Toc230597948)

[Figure S- 36: HMBC-2D spectrum of compound **4f** ( δ 6.0-11.0 ppm)	28](#_Toc230597949)

[Figure S- 37: HMBC-2D spectrum of compound **4f** ( δ 6.7-8.0 ppm)	28](#_Toc230597950)

[Figure S- 38: LC-MS spectrum of compound **4f**	29](#_Toc230597951)

[Figure S- 39: HPLC chromatogram of compound **4f**	29](#_Toc230597952)

[Figure S- 40: IR spectrum of compound **4g**	30](#_Toc230597953)

[Figure S- 41: ^1^H-NMR spectrum of compound **4g** ( δ 0-14 ppm)	30](#_Toc230597954)

[Figure S- 42: ^1^H-NMR spectrum of compound **4g** ( δ 6.6-8.0 ppm)	31](#_Toc230597955)

[Figure S- 43: ^13^C-NMR APT spectrum of compound **4g** ( δ 0-200 ppm)	31](#_Toc230597956)

[Figure S- 44: LC-MS spectrum of compound **4g**	32](#_Toc230597957)

[Figure S- 45: HPLC chromatogram of compound **4g**	32](#_Toc230597958)

[Figure S- 46: R spectrum of compound **4h**	33](#_Toc230597959)

[Figure S- 47: ^1^H-NMR spectrum of compound **4h** ( δ 0-14.0 ppm)	33](#_Toc230597960)

[Figure S- 48: ^1^H-NMR spectrum of compound **4h** ( δ 6.6-8.0 ppm)	34](#_Toc230597961)

[Figure S- 49: C-NMR APT spectrum of compound **4h** ( δ 0-180 ppm)	34](#_Toc230597962)

[Figure S- 50: 43.LC-MS spectrum of compound **4h**	35](#_Toc230597963)

[Figure S- 51: HPLC chromatogram of compound **4h**	35](#_Toc230597964)

[Figure S- 52: IR spectrum of compound **4i**	36](#_Toc230597965)

[Figure S- 53: ^1^H-NMR spectrum of compound **4i**	36](#_Toc230597966)

[Figure S- 54: ^13^C-NMR (decoupled) spectrum of compound **4i**	37](#_Toc230597967)

[Figure S- 55: LC-MS spectrum of compound **4i**	37](#_Toc230597968)

[Figure S- 56: HPLC chromatogram of compound **4i**	38](#_Toc230597969)

[Figure S- 57: IR spectrum of compound **4j**	38](#_Toc230597970)

[Figure S- 58: ^1^H-NMR spectrum of compound **4j** (δ 0.0-11.0 ppm)	39](#_Toc230597971)

[Figure S- 59: ^1^H-NMR spectrum of compound **4j** (δ 6.3-7.9 ppm)	39](#_Toc230597972)

[Figure S- 60: ^13^C-NMR APT spectrum of compound **4j**	40](#_Toc230597973)

[Figure S- 61: HSQC-2D spectrum of compound **4j** (δ 6.5-7.9 / 100-136 ppm)	40](#_Toc230597974)

[Figure S- 62: HSQC-2D spectrum of compound **4j** (δ 2.05-2.75 / 18-42 ppm)	41](#_Toc230597975)

[Figure S- 63: LC-MS spectrum of compound **4j**	41](#_Toc230597976)

[Figure S- 64: HPLC chromatogram of compound **4j**	42](#_Toc230597977)

[Figure S- 65: IR spectrum of compound **4k**	42](#_Toc230597978)

[Figure S- 66: ^1^H-NMR spectrum of compound **4k** (δ 0.0-11.0 ppm)	43](#_Toc230597979)

[Figure S- 67: ^1^H-NMR spectrum of compound **4k** (δ 6.7-8.1 ppm)	43](#_Toc230597980)

[Figure S- 68: ^13^C-NMR APT spectrum of compound **4k**	44](#_Toc230597981)

[Figure S- 69: HSQC-2D spectrum of compound **4k** (δ 6.4-9.3 / 85-140 ppm)	44](#_Toc230597982)

[Figure S- 70: HSQC-2D spectrum of compound **4k** (δ 6.6-7.8 / 96-133 ppm)	45](#_Toc230597983)

[Figure S- 71: LC-MS spectrum of compound **4k**	45](#_Toc230597984)

[Figure S- 72: HPLC chromatogram of compound **4k**	46](#_Toc230597985)

[Figure S- 73: IR spectrum of compound **4l**	46](#_Toc230597986)

[Figure S- 74: ^1^H-NMR spectrum of compound **4l**	47](#_Toc230597987)

[Figure S- 75: ^13^C-NMR APT spectrum of compound **4l**	47](#_Toc230597988)

[Figure S- 76: LC-MS spectrum of compound **4l**	48](#_Toc230597989)

[Figure S- 77: HPLC chromatogram of compound **4l**	48](#_Toc230597990)

[Figure S- 78: IR spectrum of compound **4m**	49](#_Toc230597991)

[Figure S- 79: ^1^H-NMR spectrum of compound **4m** (δ 0.0-11.0 ppm)	49](#_Toc230597992)

[Figure S- 80: ^1^H-NMR spectrum of compound **4m** (δ 6.7-7.8 ppm)	50](#_Toc230597993)

[Figure S- 81: ^13^C-NMR APT spectrum of compound **4m**	50](#_Toc230597994)

[Figure S- 82: LC-MS spectrum of compound **4m**	51](#_Toc230597995)

[Figure S- 83: : HPLC chromatogram of compound **4m**	51](#_Toc230597996)

[Figure S- 84: IR spectrum of compound **5a**	52](#_Toc230597997)

[Figure S- 85: ^1^H-NMR spectrum of compound **5a**	52](#_Toc230597998)

[Figure S- 86: ^13^C-NMR APT spectrum of compound **5a**	53](#_Toc230597999)

[Figure S- 87: LC-MS spectrum of compound **5a**	53](#_Toc230598000)

[Figure S- 88: HPLC chromatogram of compound **5a**	54](#_Toc230598001)

[Figure S- 89: IR spectrum of compound **5b**	54](#_Toc230598002)

[Figure S- 90: ^1^H-NMR spectrum of compound **5b**	55](#_Toc230598003)

[Figure S- 91: ^13^C-NMR APT spectrum of compound **5b**	55](#_Toc230598004)

[Figure S- 92: LC-MS spectrum of compound **5b**	56](#_Toc230598005)

[Figure S- 93: HPLC chromatogram of compound **5b**	56](#_Toc230598006)

[Figure S- 94: IR spectrum of compound **5c**	57](#_Toc230598007)

[Figure S- 95: ^1^H-NMR spectrum of compound **5c**	57](#_Toc230598008)

[Figure S- 96: ^13^C-NMR APT spectrum of compound **5c**	58](#_Toc230598009)

[Figure S- 97: HMBC-2D spectrum of compound **5c** (δ 0-10.0 / 0-200 ppm)	58](#_Toc230598010)

[Figure S- 98: HMBC-2D spectrum of compound **5c** (δ 6.40-8.0 / 70-150 ppm)	59](#_Toc230598011)

[Figure S- 99: HMBC-2D spectrum of compound **5c** (δ 6.80-7.65 / 110-150 ppm)	60](#_Toc230598012)

[Figure S- 100: LC-MS spectrum of compound **5c**	60](#_Toc230598013)

[Figure S- 101: HPLC chromatogram of compound **5c**	61](#_Toc230598014)

[Figure S- 102: IR spectrum of compound **5d**	61](#_Toc230598015)

[Figure S- 103: ^1^H-NMR spectrum of compound **5d**	62](#_Toc230598016)

[Figure S- 104: ^13^C-NMR APT spectrum of compound **5d**	63](#_Toc230598017)

[Figure S- 105: LC-MS spectrum of compound **5d**	63](#_Toc230598018)

[Figure S- 106: HPLC chromatogram of compound **5d**	64](#_Toc230598019)

[Figure S- 107: IR spectrum of compound **5e**	64](#_Toc230598020)

[Figure S- 108: ^1^H-NMR spectrum of compound **5e**	65](#_Toc230598021)

[Figure S- 109: ^13^C-NMR APT spectrum of compound **5e**	65](#_Toc230598022)

[Figure S- 110: HSQC-2D spectrum of compound **5e** (δ 2.0-8.5 / 15-150 ppm)	66](#_Toc230598023)

[Figure S- 111: LC-MS spectrum of compound **5e**	66](#_Toc230598024)

[Figure S- 112: HPLC chromatogram of compound **5e**	67](#_Toc230598025)

[Figure S- 113: IR spectrum of compound **5f**	67](#_Toc230598026)

[Figure S- 114: ^1^H-NMR spectrum of compound **5f**	68](#_Toc230598027)

[Figure S- 115: ^13^C-NMR APT spectrum of compound **5f**	68](#_Toc230598028)

[Figure S- 116: LC-MS spectrum of compound **5f**	69](#_Toc230598029)

[Figure S- 117: HPLC chromatogram of compound **5f**	69](#_Toc230598030)

[Figure S- 118: IR spectrum of compound **5g**	70](#_Toc230598031)

[Figure S- 119: ^1^H-NMR spectrum of compound **5g**	70](#_Toc230598032)

[Figure S- 120: ^13^C-NMR APT spectrum of compound **5g**	71](#_Toc230598033)

[Figure S- 121: LC-MS spectrum of compound **5g**	71](#_Toc230598034)

[Figure S- 122: HPLC chromatogram of compound **5g**	72](#_Toc230598035)

[Figure S- 123: IR spectrum of compound **5h**	72](#_Toc230598036)

[Figure S- 124: ^1^H-NMR spectrum of compound **5h**	73](#_Toc230598037)

[Figure S- 125: ^13^C-NMR APT spectrum of compound **5h**	73](#_Toc230598038)

[Figure S- 126: LC-MS spectrum of compound **5h**	74](#_Toc230598039)

[Figure S- 127: HPLC chromatogram of compound **5h**	74](#_Toc230598040)

[Figure S- 128: IR spectrum of compound **5i**	75](#_Toc230598041)

[Figure S- 129: ^1^H-NMR spectrum of compound **5i**	75](#_Toc230598042)

[Figure S- 130: ^13^C-NMR APT spectrum of compound **5i**	76](#_Toc230598043)

[Figure S- 131: LC-MS spectrum of compound **5i**	76](#_Toc230598044)

[Figure S- 132: HPLC chromatogram of compound **5i**	77](#_Toc230598045)

[Figure S- 133: ^1^H-NMR spectrum of compound **5j**	77](#_Toc230598046)

[Figure S- 134: ^13^C-NMR APT spectrum of compound **5j**	78](#_Toc230598047)

[Figure S- 135: LC-MS spectrum of compound **5j**	78](#_Toc230598048)

[Figure S- 136: HPLC chromatogram of compound **5j**	79](#_Toc230598049)

[Figure S- 137: ^1^H-NMR APT spectrum of compound **5k**	79](#_Toc230598050)

[Figure S- 138: ^13^C-NMR APT spectrum of compound **5k**	80](#_Toc230598051)

[Figure S- 139: LC-MS spectrum of compound **5k**	80](#_Toc230598052)

[Figure S- 140: HPLC chromatogram of compound **5k**	81](#_Toc230598053)

[Figure S- 141: ^1^H-NMR spectrum of compound **5l**	81](#_Toc230598054)

[Figure S- 142: ^13^C-NMR APT spectrum of compound **5l**	82](#_Toc230598055)

[Figure S- 143: LC-MS spectrum of compound **5l**	82](#_Toc230598056)

[Figure S- 144: HPLC chromatogram of compound **5l**	83](#_Toc230598057)

[Figure S- 145: IR spectrum of compound **5m**	83](#_Toc230598058)

[Figure S- 146: ^1^H-NMR spectrum of compound **5m**	84](#_Toc230598059)

[Figure S- 147: ^13^C-NMR APT spectrum of compound **5m**	84](#_Toc230598060)

[Figure S- 148: LC-MS spectrum of compound **5m**	85](#_Toc230598061)

[Figure S- 149: HPLC chromatogram of compound **5m**	85](#_Toc230598062)

[Figure S- 150: IR spectrum of compound **5n**	86](#_Toc230598063)

[Figure S- 151: ^1^H-NMR APT spectrum of compound **5n**	86](#_Toc230598064)

[Figure S- 152: ^13^C-NMR APT spectrum of compound **5n**	87](#_Toc230598065)

[Figure S- 153: HMBC-2D spectrum of compound **5n** (δ 2.2-7.8 / 35-175 ppm)	87](#_Toc230598066)

[Figure S- 154: HMBC-2D spectrum of compound **5n** (δ 6.8-7.9 / 65-150 ppm)	88](#_Toc230598067)

[Figure S- 155: LC-MS spectrum of compound **5n**	88](#_Toc230598068)

[Figure S- 156: HPLC chromatogram of compound **5n**	89](#_Toc230598069)

[Figure S- 157: Conformational superposition of the experimental X-ray crystal structure and the predicted lowest-energy conformation of the title compound (RMSD = 1.5670 Å)	91](#_Toc230598070)

**Tables**

[Tablo S 1. In silico physicochemical properties of compounds **4a-m** and **5a-n**	89](#_Toc230567844)

**Checkcif Report of compound 5h**

#### **Synthesis of 1-methyl-1H-indole-2,3-diones (2a-n)**

Compound **2a-n** was synthesized as described previously (24,25).

#### **Synthesis of 5-(trifluoromethyl)-3H-spiro[1,3-benzothiazole-2,3′-indol]-2′(1′H)-ones (4i–m and 5a-n)**

Compound **4i-m** and **5a-n** derivatives were synthesized by adding 2-amino-4-(trifluoromethyl)benzenethiol **3** (3.5 mmol) to a solution of 1*H*-indole-2,3-diones **1i-m/2a-n** (3.5 mmol) in absolute ethanol (15 mL). The mixture was refluxed in a water bath for 3 hours. The resulting product was filtered off and purified by crystallization from an ethanol-water mixture (24,25).

***5-(Trifluoromethyl)-3H-spiro[benzo[d]thiazole-2,3’-indolin]-2’one (4i)***

Grey powder (yield %93), molecular mass 322.30 g/mol; melting point 218-220 °C; **IR (KBr) cm^-1^**: 3288, 3199 (N-H), 3093, 3064, 3030 (aromatic C-H), 1722 (C=O), 1618, 1585, 1452 (C=C); **^1^H-NMR** (DMSO-*d_6_*, 500 MHz) **δ** (ppm): 6.71 (d, *J* = 1.5 Hz, 1H, b.t. C_4_-H), 6.87 (d, *J* = 7.7 Hz, 1H, ind. C_7_-H), b.t. C_6_-H), 6,95 (d, *J* = 7.9 Hz, 1H, b.t. C_6_-H), 7.09 (t, *J* = 7.5 Hz, 1H, ind. C_5_-H), 7.27 (d, *J* = 7.9 Hz, 1H, b.t. C_7_-H), 7.33 (t, *J* = 7.7 Hz, 1H, ind. C_6_-H), 7.61 (d, *J* = 7.4 Hz, 1H, ind. C_4_-H), 7.76 (s, 1H, b.t. N-H), 10.50 (s, 1H, ind. N-H); **^13^C NMR (decoupled)** (125 MHz, DMSO-*d6*) **δ** (ppm) : 75.32 (spiro C), 103.78 (q, *J* = 3.9 Hz, b.t. C_4_), 110.74 (ind. C_7_), 115.72 (q, *J* = 4.2 Hz, b.t. C_6_), 121.85 (b.t. C_7_), 123.17 (ind. C_5_), 124,97 (q, *J =* 272.1 Hz, CF_3_), 126.30 (ind. C_3a_), 127,28 (q, *J =* 31.5 Hz, b.t. C_5_), 129.49 (ind. C_4_), 130.27 (ind. C_6_), 131.36 (b.t. C_7a_), 141.86 (ind. C_7a_), 148.37 (b.t. C_3a_), 176.23 (ind. C_2_); **LC-MS (ESI+):** 323.0 ([M+H]^+^, 100); **Analyses (%) cald for C_15_H_9_F_3_N_2_OS:** C, 55.90; H, 2.81; N, 8.69; S, 9.95. Found: C, 55.84; H, 2.83; N, 8.84; S, 9.99. HPLC purity: 100.0%

***5'-Methyl-5-(trifluoromethyl)-3H-spiro[benzo[d]thiazole-2,3'-indolin]-2'-one (4j)***

Salmon pink powder (yield %98), molecular mass 336.33 g/mol; melting point 230-232 °C; **IR (KBr) cm^-1^**: 3380, 3284, 3174 (N-H), 3086, 3039 (aromatic C-H), 2985, 2879 (aliphatic C-H), 1710 (C=O), 1625, 1583, 1494 (C=C); **^1^H-NMR** (DMSO-*d_6_*, 500 MHz) **δ** (ppm): 2.27 (s, 3H, CH_3_), 6.69 (d, *J* = 1.4 Hz, 1H, b.t. C_4_-H), 6.76 (d, *J* = 7.9 Hz, 1H, ind. C_7_-H), 6,94 (d, *J* = 7.9 Hz, 1H, b.t. C_6_-H), 7.12 (d, *J* = 7.9 Hz, 1H, ind. C_6_-H), 7.26 (d, *J* = 7.9 Hz, 1H, b.t. C_7_-H), 7.42 (s, 1H, ind. C_4_-H), 7.75 (s, 1H, b.t. N-H), 10.40 (s, 1H, ind.N-H); **^13^C NMR (APT)** (125 MHz, DMSO-*d6*) **δ** (ppm) : 20.95 (ind. CH_3_), 75.42 (spiro C), 103.68 (q, *J* = 3.9 Hz, b.t. C_4_), 110.51 (ind. C_7_), 115.66 (q, *J* = 4.2 Hz, b.t. C_6_), 121.77 (b.t. C_7_), 123.90 (ind. C_3a_), 126,70 (ind. C_4_), 127,04 (q, *J =* 31.5 Hz, b.t. C_5_), 127,14 (q, *J =* 270,06 Hz, CF_3_), 129.61 (b.t. C_7a_), 130.24 (ind. C_5_), 131.58 (ind. C_6_), 139.28 (ind. C_7a_), 148.35 (b.t. C_3a_), 176.25 (ind. C_2_); **LC-MS (ESI+):** 359.0 ([M+Na]^+^; 55), 337.0 ([M+H]^+^, 100); **Analyses (%) cald for C_16_H_11_F_3_N_2_OS:** C, 57.14; H, 3.30; N, 8.33; S, 9.53. Found: C, 57.13; H, 3.28; N, 8.50; S, 9.6. HPLC purity: 98.9%

***5'-(Trifluoromethoxy)-5-(trifluoromethyl)-3H-spiro[benzo[d]thiazole-2,3'-indolin]-2'-one (4k)***

Orange powder (yield %94), molecular mass 406.30 g/mol; melting point 199-202 °C; **IR (KBr) cm^-1^**: 3288, 3186 (N-H), 3089, 3037 (aromatic C-H), 1728 (C=O), 1629, 1583, 1483 (C=C); **^1^H-NMR** (DMSO-*d_6_*, 500 MHz) **δ** (ppm): 6.75 (d, *J* = 1.4 Hz, 1H, b.t. C_4_-H), 6.97 (d, *J* = 8.5 Hz, 1H, b.t. C_6_-H, ind. C_7_-H), 7.29 (d, *J* = 7.9 Hz, 1H, b.t. C_7_-H), 7.35 (d, *J* = 8,5 Hz, 1H, ind. C_6_-H), 7.60 (d, *J* = 1,9 Hz, 1H, ind. C_4_-H), 7.79 (s, 1H, b.t. N-H), 10.69 (s, 1H, ind. N-H); **^13^C NMR (APT)** (125 MHz, DMSO-*d6*) **δ** (ppm) : 20,95 (OCH_3_), 75.22 (spiro C), 104.02 (q, *J* = 3.9 Hz, b.t. C_4_), 111.89 (ind. C_7_), 115.91 (q, *J* = 4.2 Hz, b.t. C_6_), 119.86 (b.t. C_7_), 120.50 (OCF_3_), 121.94 (ind. C_4_), 124.81 (ind. C_6_), (123.90 ind. C_3a_), 127.20 (q, *J =* 31.5 Hz, b.t. C_5_), 129.0 (q, *J =* 272.3, CF_3_), 131.49 (b.t. C_7a_), 141.10 (ind. C_7a_), 144,12 (ind. C_5_), 148.06 (b.t. C_3a_), 176.35 (ind. C_2_); **LC-MS (ESI+):** 407.0([M+H]^+^ ; 60), 320 (100); **Analyses (%) cald for C_16_H_8_F_6_N_2_OS:** C, 47.30; H, 1.98; N, 6.89; S, 7.89. Found: C, 47.50; H, 1.99; N, 7.08; S, 7.92. HPLC purity: 98.5%

***5'-Fluoro-5-(trifluoromethyl)-3H-spiro[benzo[d]thiazole-2,3'-indolin]-2'-one (4l)***

Sand powder (yield %96), molecular mass 340.29 g/mol; melting point 229-230 °C; **IR (KBr) cm^-1^**: 3284, 3184 (N-H), 3091, 3062, 3037 (aromatic C-H), 1722 (C=O), 1614, 1485 (C=C); **^1^H-NMR** (DMSO-*d_6_*, 500 MHz) **δ** (ppm): 6.74 (d, *J* = 1.4 Hz, 1H, b.t. C_4_-H), 6.88 (m, 1H, ind. C_6_-H), 6.96 (d, *J* = 7.9 Hz, 1H, b.t. C_6_-H), 7.18 (d, *J* = 8.7 Hz, 1H, ind. C_6_-H), 7.29 (d, *J* = 7.9 Hz, 1H, b.t. C_7_-H), 7.50 (d, *J* = 7,9 Hz, 1H, ind. C_4_-H), 7.79 (s, 1H, b.t. N-H), 10.52 (s, 1H, ind.N-H); **^13^C NMR (APT)** (125 MHz, DMSO-*d6*) **δ** (ppm) : 75.45 (spiro C), 103.94 (q, *J* = 3.9 Hz, b.t. C_4_), 111.81 (ind. C_7_), 113.80 (ind. C_4_), 115.85 (q, *J* = 4.4 Hz, b.t. C_6_), 117, 87 (ind. C_6_), 121.89 (b.t. C_7_), 124 (q, *J*= 272.2 Hz, CF_3_), 127.16 (q, *J* = 31.5 Hz, b.t. C_5_), 130.05 (ind. C_3a_), 131.33 (b.t. C_7a_), 137.99 (ind. C_7a_), 148.19 (b.t. C_3a_), 158.86 (ind. C_5_), 176.36 (ind. C_2_); **LC-MS (ESI+):** 363.0 ([M+Na]^+^; 100), 341.85 ([M+H]^+^, 86); **Analyses (%) cald for C_15_H_8_F_4_N_2_OS:** C, 52.94; H, 2.37; N, 8.23; S, 9.42. Found: C, 53.13; H, 2.31; N, 8.34; S, 9.38. HPLC purity: 100.0%

***5'-Chloro-5-(trifluoromethyl)-3H-spiro[benzo[d]thiazole-2,3'-indolin]-2'-one (4m)***

Orange powder (yield %90), molecular mass 356.75 g/mol; melting point 227-230 °C; **IR (KBr) cm^-1^**: 3388, 3290 (N-H), 3089, 3057, 3032 (aromatic C-H), 1712 (C=O), 1622, 1583, 1469 (C=C); **^1^H-NMR** (DMSO-*d_6_*, 500 MHz) **δ** (ppm): 6.74 (d, *J* = 1.4 Hz, 1H, b.t. C_4_-H), 6.89 (d, *J* = 8.4 Hz 1H, b.t. C_6_-H), 6.96 (d, *J* = 8,8 Hz, 1H, ind. C_7_-H), 7.29 (d, *J* = 7.9 Hz, 1H, b.t. C_7_-H), 7.38 (d, *J* = 8.3 Hz, 1H, ind. C_6_-H), 7.62 (d, *J* = 2.1 Hz, 1H, ind. C_4_-H), 7.77 (s, 1H, b.t. N-H), 10.63 (s, 1H, ind.N-H); **^13^C NMR (APT)** (151 MHz, DMSO-*d6*) **δ** (ppm) : 75.22 (spiro C), 104.05 (q, *J*= 3.6 Hz, b.t. C_4_), 112.36 (ind. C_7_), 115.94 (q, *J*= 4.2 Hz, b.t. C_6_), 121.90 (b.t. C_7_), 124.94 (q, *J*= 271.8 Hz, CF_3_), 127.03 (ind. C_5_), 127.21 (q, *J*= 31.3 Hz, b.t. C_5_), 130.01 (ind. C_3a_), 131.18 (ind. C_6_), 131.78 (b.t. C_7a_), 140.71 (ind. C_7a_), 148.08 (b.t. C_3a_), 176.01 (ind. C_2_); **LC-MS (ESI+):** 381.0, 379.0 ([M+Na]^+^; 51, 17), 357.0, 359.0 ([M+H]^+^, 100, 30); **Analyses (%) cald for C_15_H_8_ClF_3_N_2_OS:** C, 50.50; H, 2.36; N, 7.85; S, 8.99. Found: C, 50.89; H, 2.26; N, 7.85; S, 8.99. HPLC purity: 99.8%

***1'-Methyl-5-(trifluoromethyl)-3H-spiro[benzo[d]thiazole-2,3'-indolin]-2'-one (5a)***

Silvery white powder (yield 82%), molecular mass 336.33 g/mol; melting point 211-212 °C; **IR (KBr) cm^-1^**: 3319 (N-H), 3065 (aromatic C-H), 2891, 2837 (aliphatic C-H), 1705 (C=O) 1610, 1472, 1458 (C=C); **^1^H-NMR** (DMSO-*d_6_*, 400 MHz) **δ** (ppm): 3.11 (s, 3H, ind. C_1_-CH_3_), 6.71 (d, *J* = 1.8 Hz, 1H, b.t. C_4_-H), 6.94 (d, *J* = 7.9 Hz, 1H, b.t. C_6_-H), 7.05 (d, *J* = 7.8 Hz, 1H, ind. C_7_-H), 7.14 (t, *J* = 7.5 Hz, 1H, ind. C_5_-H), 7.26 (d, *J* = 7.9 Hz, 1H, b.t. C_7_-H), 7.41 (t, *J* = 7.7 Hz, 1H, ind. C_6_-H), 7.62 (d, *J* = 7.4 Hz, 1H, ind. C_4_-H), 7.68 (s, 1H, b.t. N-H); **^13^C NMR (APT)** (126 MHz, DMSO-*d6*) **δ** (ppm) : 26.76 (ind. C_1_-CH_3_), 75.02 (spiro C), 103.97 (q, *J* = 3.9 Hz, b.t. C_4_), 109.71 (ind. C_7_), 115.84 (q, *J* = 4.2 Hz, b.t. C_6_), 121.88 (b.t. C_7_), 123.77 (ind. C_5_), 124,97 (q, *J =* 272,14 Hz, CF_3_), 125.89 (ind. C_4_), 127,38 (q, *J =* 31,51 Hz, b.t. C_5_), 129.02 (ind. C_3a_), 130.23 (b.t. C_7a_), 131.44 (ind. C_6_), 143.34 (ind. C_7a_), 148.18 (b.t. C_3a_), 174.53 (ind. C_2_); **LC-MS (ESI+):** 359.0 ([M+Na]^+^, 100), 337.1 ([M+H]^+^, 70); **Analyses (%) cald for C_16_H_11_F_3_N_2_OS:** C, 57.14; H, 3.30; N, 8.33; S, 9.53. Found: C, 56.86; H, 3.22; N, 8.24; S, 9.53. HPLC purity: 100.0%

***1',5'-Dimethyl-5-(trifluoromethyl)-3H-spiro[benzo[d]thiazole-2,3'-indolin]-2'-one (5b)***

Silvery white powder (yield 73%), molecular mass 350.35 g/mol; melting point 248-251 °C; **IR (KBr) cm^-1^**: 3287 (N-H), 3057 (aromatic C-H), 2940, 2878 (aliphatic C-H), 1709 (C=O) 1622, 1603, 1499 (C=C); **^1^H-NMR** (DMSO-*d_6_*, 400 MHz) **δ** (ppm): 2.28 (s, 3H, ind. C_5_-CH_3_), 3.09 (s, 3H, ind. C_1_-CH_3_), 6.69 (d, *J* = 1.8 Hz, 1H, b.t. C_4_-H), 6.92 (dd, *J*=7.8 Hz, 1H, ind. C_7_-H), 6.94 (d, *J*=7.9 Hz, 1H, b.t. C_6_-H), 7.21 (d, *J* = 7.9 Hz, 1H, ind. C_6_-H), 7.25 (d, *J* = 7.9 Hz, 1H, b.t. C_7_-H), 7.44 (d, *J* = 1.7 Hz, 1H, ind. C_4_-H), 7.67 (s, 1H, b.t. N-H); **^13^C NMR (APT)** (126 MHz, DMSO-*d6*) **δ** (ppm) : 20.94 (ind. C_5_-CH_3_), 26.77 (ind. C_1_-CH_3_), 75.11 (spiro C), 103.86 (q, *J* = 3.9 Hz, b.t. C_4_), 109.48 (ind. C_7_), 115.77 (q, *J* = 4.2 Hz, b.t. C_6_), 121.79 (b.t. C_7_), 124,99 (q, *J =* 270,06 Hz, CF_3_), 126.43 (ind. C_4_), 127,11 (q, *J =* 31,50 Hz, b.t. C_5_), 129.10 (ind. C_3a_), 130.27 (b.t. C_7a_), 131.58 (ind. C_6_), 133.01(ind. C_5_), 140.88 (ind. C_7a_), 148.16 (b.t. C_3a_), 174.49 (ind. C_2_); **LC-MS (ESI+):** 373.1 ([M+Na]^+^, 100), 351.1 ([M+H]^+^, 71); **Analyses (%) cald for C_17_H_13_F_3_N_2_OS:** C, 58.28; H, 3.74; N, 8.00; S, 9.15. Found: C, 57.95; H, 3.63; N, 7.92; S, 9.11. HPLC purity: 95.17%

***1'-Methyl-5'-(trifluoromethoxy)-5-(trifluoromethyl)-3H-spiro[benzo[d]thiazole-2,3'-indolin]-2'-one (5c)***

Silvery white powder (yield 54%), molecular mass 420.32 g/mol; melting point 178-180 °C; **IR (KBr) cm^-1^**: 3329 (N-H), 3078, 3032 (aromatic C-H), 2949, 2887 (aliphatic C-H), 1711 (C=O) 1620, 1497, 1472 (C=C); **^1^H-NMR** (DMSO-*d_6_*, 500 MHz) **δ** (ppm): 3.16 (s, 3H, ind. C_1_-CH_3_), 6.80 (d, *J* = 1.8 Hz, 1H, b.t. C_4_-H), 6.98 (ddd, *J* = 8.0, 1.8, 0.9 Hz, 1H, b.t. C_6_-H), 7.19 (d, *J* = 8.6 Hz, 1H, ind. C_7_-H), 7.31 (d, *J* = 7.9 Hz, 1H, b.t. C_7_-H), 7.47 (ddd, *J* = 8.5, 2.6, 1.0 Hz, 1H, ind. C_6_-H), 7.64 (dd, *J* = 2.3, 1.1 Hz, 1H, ind. C_4_-H), 7.72 (s, 1H, b.t. N-H). **HMBC-2D** (126 MHz, DMSO-*d6*) **δ** (ppm): 26.98 (N-CH_3_), 74.82 (spiro C), 104.23 (q, *J* = 3.9 Hz, b.t. C_4_), 110.96 (ind. C_7_), 116.02 (q, *J* = 4.3 Hz, b.t.C_6_), 119.49 (ind. C_4_), 120,61 (q, *J*= 255.78, OCF_3_), 121.93 (b.t. C_7_), 124.75 (ind. C_6_), 124,93 (q, *J*=272.16 Hz CF_3_), 127,26 (q *J*=31.40 Hz, b.t.C_5_), 129.90 (b.t. C_7a_), 131.08 (ind. C_3a_), 142.54 (ind. C_7a_), 144.64 (q, *J* = 2.0 Hz, ind. C_5_), 147.87 (b.t. C_3a_), 174.62 (C=O); **LC-MS (ESI):** 443.0 ([M+Na]^+^, 20), 421.0 ([M+H]^+^, 20); **Analyses (%) cald for C_17_H_10_F_6_N_2_OS.^1/10^H_2_O:** C, 48.37; H, 2.44; N, 6.64; S, 7.59. Found: C, 47.98; H, 2.39; N, 6.72; S, 7.57. HPLC purity: 98.7%

***5'-Fluoro-1'-methyl-5-(trifluoromethyl)-3H-spiro[benzo[d]thiazole-2,3'-indolin]-2'-one (5d)***

Silvery white powder (yield 74%), molecular mass 354.32 g/mol; melting point 210-211 °C; **IR (KBr) cm^-1^**: 3321 (N-H), 3089 (aromatic C-H), 2942, 2887 (aliphatic C-H), 1707 (C=O) 1602, 1493, 1474 (C=C); **^1^H-NMR** (DMSO-*d_6_*, 400 MHz) **δ** (ppm): 3.11 (s, 3H, ind. C_1_-CH_3_), 6.74 (d, *J* = 1.8 Hz, 1H, b.t. C_4_-H), 6.95 (d, *J* = 7.9 Hz, 1H, b.t. C_6_-H), 7.08 (dd, *J* = 8.6, 4.1 Hz, 1H, ind. C_7_-H), 7.24-7.27 (m, 2H, b.t. C_7_-H, ind. C_6_-H), 7.52 (dd, *J* = 7.8, 2.7 Hz, 1H, ind. C_4_-H), 7.70 (s, 1H, b.t. N-H); **^13^C NMR (APT)** (126 MHz, DMSO-*d6*) **δ** (ppm): 26.93 (ind. C_1_-CH_3_), 75.04 (d, *J* = 1.7 Hz, spiro C), 104.13 (q, *J* = 3.8 Hz, b.t. C_4_), 110.89 (d, *J* = 7.9 Hz, ind. C_7_), 113.64 (d, *J* = 25.3 Hz, ind. C_4_), 115.96 (q, *J* = 4.4 Hz, b.t. C_6_), 117.77 (d, *J* = 23.5 Hz, ind. C_6_), 121.91 (b.t. C_7_), 124,94 (q, *J*= 272.2 Hz, CF_3_), 127.21 (q, *J* = 31.6 Hz, b.t. C_5_), 130, 02 (b.t. C_7a_), 130.92 (d, *J* = 7.8 Hz, ind. C_3a_), 139.54 (d, *J* = 1.8 Hz, ind. C_7a_), 148.00 (b.t. C_3a_), 159.36 (d, *J* = 239.5 Hz, ind. C_5_), 174.54 (ind. C_2_); **LC-MS (ESI+):** 377.0 ([M+Na]^+^, 100), 355.0 ([M+H]^+^, 76); **Analyses (%) cald for C_16_H_10_F_4_N_2_OS:** C, 54.24; H, 2.84; N, 7.91; S, 9.05. Found: C, 53.85; H, 2.79; N, 7.86; S, 8.96. HPLC purity: 99.2%

***5'-Chloro-1'-methyl-5-(trifluoromethyl)-3H-spiro[benzo[d]thiazole-2,3'-indolin]-2'-one (5e)***

Silvery white powder (yield 71%), molecular mass 370.77 g/mol; melting point 216-218 °C; **IR (KBr) cm^-1^**: 3291 (N-H), 3092, 3063 (aromatic C-H), 2949, 2883 (aliphatic C-H), 1707 (C=O) 1610, 1464, 1435 (C=C); **^1^H-NMR** (DMSO-*d_6_*, 400 MHz) **δ** (ppm): 3.11 (s, 3H, ind. C_1_-CH_3_), 6.74 (d, *J* = 1.8 Hz, 1H, bt. C_4_-H), 6.95 (d, 1H, *J* = 7.9 Hz, b.t. C_6_-H), 7.09 (d, *J* = 8.4 Hz, 1H, ind. C_7_-H), 7.28 (d, *J* = 7.9 Hz, 1H, b.t. C_7_-H), 7.48 (dd, *J* = 8.4, 2.2 Hz, 1H, ind. C_6_-H), 7.64 (d, *J* = 2.2 Hz, 1H, ind. C_4_-H); 7.68 (s, 1H, b.t. N-H); **^13^C NMR (APT)** (126 MHz, DMSO-*d6*) **δ** (ppm): 26.94 (ind. C_1_-CH_3_), 74.84 (spiro C), 104.23 (q, *J*= 3.8 Hz, b.t. C_4_), 111.39 (ind. C_7_), 116.02 (q, *J*= 4.2 Hz, b.t. C_6_), 121.92 (b.t. C_7_), 125.14 (q, *J*= 272.2 Hz, CF_3_), 127.23 (q, *J*= 31.5 Hz, b.t. C_5_), 127.73 (ind. C_5_), 129.99 (ind. C_3a_), 131.18 (ind. C_6_), 131.26 (b.t. C_7a_), 142.22 (ind. C_7a_), 147.89 (b.t. C_3a_), 174.31 (ind. C_2_); **LC-MS (ESI+):** 393.0, 395.0 ([M+Na]^+^; 100, 50), 371.0, 373.0 ([M+H]^+^, 80, 30); **Analyses (%) cald for C_16_H_10_ClF_3_N_2_OS:** C, 51.83; H, 2.72; N, 7.56; S, 8.65. Found: C, 51.52; H, 2.69; N, 7.48; S, 8.62. HPLC purity: 98.9%

***5'-Bromo-1'-methyl-5-(trifluoromethyl)-3H-spiro[benzo[d]thiazole-2,3'-indolin]-2'-one (5f)***

Silver powder (yield 66%), molecular mass 415.22 g/mol; melting point 236-238 °C; **IR (KBr) cm^-1^**: 3291 (N-H), 3063 (aromatic C-H), 2949, 2881 (aliphatic C-H), 1707 (C=O) 1485, 1470 (C=C); **^1^H-NMR** (DMSO-*d_6_*, 400 MHz) **δ** (ppm): 3.10 (s, 3H, ind. C_1_-CH_3_), 6.74 (d, *J* = 1.8 Hz, 1H, bt. C_4_-H), 6.95 (d, 1H, *J* = 7.9 Hz, b.t. C_6_-H), 7.04 (d, *J* = 8.4 Hz, 1H, ind. C_7_-H), 7.28 (d, *J* = 7.9 Hz, 1H, b.t. C_7_-H), 7.61 (dd, *J* = 8.4, 2.1 Hz, 1H, ind. C_6_-H), 7.67 (s, 1H, b.t. N-H), 7.73 (d, *J* = 2.0 Hz, 1H, ind. C_4_-H); **^13^C NMR (APT)** (126 MHz, DMSO-*d6*) **δ** (ppm): 26.93 (ind. C_1_-CH_3_), 74.78 (spiro C), 104.21 (q, *J*= 3.9 Hz, b.t. C_4_), 111.89 (ind. C_7_), 115.27 (ind. C_5_), 116.02 (q, *J*= 4.3 Hz, b.t. C_6_), 121.93 (b.t. C_7_), 124.94 (q, *J*= 272.3 Hz, CF_3_), 127.23 (q, *J*=31.5 Hz, b.t. C_5_), 128.50 (ind. C_4_), 129.98 (ind. C_3a_), 131.61 (b.t. C_7a_), 134.03 (ind. C_6_), 142.64 (ind. C_7a_), 147.86 (b.t. C_3a_), 174.18 (ind. C_2_); **LC-MS (ESI+):** 438.9, 440.9 ([M+Na]^+^; 17, 15), 416.9, 418.9 ([M+H]^+^, 18, 16); **Analyses (%) cald for C_16_H_10_BrF_3_N_2_OS:** C, 46.28; H, 2.43; N, 6.75; S, 7.72. Found: C, 45.98; H, 2.43; N, 6.68; S, 7.63. HPLC purity: 97.5%

***5'-İodo-1'-methyl-5-(trifluoromethyl)-3H-spiro[benzo[d]thiazole-2,3'-indolin]-2'-one (5g)***

White powder (yield 71%), molecular mass 462.22 g/mol; melting point 249-251°C; **IR (KBr) cm^-1^**: 3291 (N-H), 3184 (aromatic C-H), 2949, 2882 (aliphatic C-H), 1707 (C=O) 1605, 1470 (C=C); **^1^H-NMR** (DMSO-*d_6_*, 400 MHz) **δ** (ppm): 3.09 (s, 3H, ind. C_1_-CH_3_), 6.73 (d, *J* = 1.8 Hz, 1H, b.t. C_4_-H), 6.92 (d, 1H, *J* = 8.2 Hz, ind. C_7_-H), 6.95 (d, *J* = 7.7 Hz, 1H, b.t. C_6_-H), 7.28 (d, *J* = 7.9 Hz, 1H, b.t. C_7_-H), 7.66 (s, 1H, b.t. N-H), 7.76 (dd, *J* = 8.2, 1.8 Hz, 1H, ind. C_6_-H), 7.84 (d, *J* = 1.8 Hz, 1H, ind. C_4_-H); **^13^C NMR (APT)** (126 MHz, DMSO-*d6*) **δ** (ppm): 26.87 (ind. C_1_-CH_3_), 74.61 (spiro C), 86.46 (ind. C_5_), 104.17 (q, *J*= 3.9 Hz, b.t. C_4_), 112.29 (ind. C_7_), 115.99 (q, *J*= 4.3 Hz, b.t. C_6_), 121.91 (b.t. C_7_), 124.94 (q, *J*= 272.2 Hz, b.t. CF_3_), 127.20 (q, *J*= 31.6 Hz, b.t. C_5_), 129.99 (ind. C_3a_), 131.76 (b.t. C_7a_), 133.94 (ind. C_4_), 139.80 (ind. C_6_), 143.10 (ind. C_7a_), 147.85 (b.t. C_3a_), 173.97 (ind. C_2_); **LC-MS (ESI+):** 485.0 ([M+Na]^+^; 42), 463.0, ([M+H]^+^, 35), 318.2 (100); **Analyses (%) cald for C_16_H_10_BrF_3_N_2_OS:** C, 41.57; H, 2.18; N, 6.06; S, 6.94. Found: C, 41.33; H, 2.17; N, 6.03; S, 6.83. HPLC purity: 97.1%

***1'-Methyl-5'-nitro-5-(trifluoromethyl)-3H-spiro[benzo[d]thiazole-2,3'-indolin]-2'-one (5h)***

Yellow powder (yield %50), molecular mass 381.32 g/mol; melting point 171-174°C; **IR (KBr) cm^-1^**: 3312 (N-H), 3109, 3082 (aromatic C-H), 2947 (aliphatic C-H), 1711(C=O) 1610, 1527, 1491 (C=C); **^1^H-NMR** (DMSO-*d_6_*, 400 MHz) **δ** (ppm): 3.21 (s, 3H, ind. C_1_-CH_3_), 6.83 (d, *J* = 1.8 Hz, 1H, b.t. C_4_-H), 6.99 (d, 1H, *J* = 8.0 Hz, b.t. C_6_-H), 7.30-7.33 (m, 2H, b.t. C_7_-H, ind. C_7_-H), 7.68 (s, 1H, b.t. N-H), 8.31-8.40 (m, 2H, ind. C_6_-H, ind. C_4_-H); **^13^C NMR (APT)** (126 MHz, DMSO-*d6*) **δ** (ppm): 26.88 (ind. C_1_-CH_3_), 73.90 (spiro C), 104.12 (d, *J=* 4.2 Hz*,* b.t. C_4_), 109.83 (ind. C_7_), 115.83 (d, *J*=4.8 Hz b.t. C_6_), 120.50 (ind. C_4_), 121.60 (b.t. C_7_), 124.94 (q, *J*= 272.2 Hz, b.t. CF_3_), 126.91 (q, *J*= 39.6 Hz, b.t. C_5_), 127.71 (ind. C_6_), 129.36 (ind. C_3a_), 130.20 (b.t. C_7a_), 143.20 (ind. C_7a_)_,_ 147.13 (ind. C_5_), 148.84 (b.t. C_3a_), 174.66 (ind. C_2_); **LC-MS (ESI+):** 382.0 ([M+H]^+^, 12), 325.2 (100); **Analyses (%) cald for C_16_H_10_F_3_N_3_O_3_S:** C, 50.40; H, 2.64; N, 11.02; S, 8.41. Found: C, 50.42; H, 2.69; N, 11.16; S, 8.41. HPLC purity: 97.3%

***1',7'-Dimethyl-5-(trifluoromethyl)-3H-spiro[benzo[d]thiazole-2,3'-indolin]-2'-one (5i)***

Silvery white powder (yield %93), molecular mass 350.35 g/mol; melting point 231-232°C; **IR (KBr) cm^-1^**: 3285(N-H), 3094, 3065 (aromatic C-H), 2986 (aliphatic C-H), 1712(C=O), 1687, 1599, 1454 (C=C); **^1^H-NMR** (DMSO-*d_6_*, 500 MHz) **δ** (ppm): 2.56 (s, 3H, ind. C_7_-CH_3_), 3.40 (s, 3H, ind. C_1_-CH_3_), 6.74 (d, *J* = 1.8 Hz, 1H, b.t. C_4_-H), 6.96 (dd, *J* = 7.9, 1.8 Hz, 1H, b.t. C_6_-H ), 7.05 (t, *J* = 7.6 Hz, 1H, ind. C_5_-H), 7.18 (d, *J* = 7.7 Hz, 1H, ind. C_6_-H), 7.27 (d, 1H, *J* = 7.9 Hz, b.t. C_7_-H), 7.50 (dd, *J* = 7.5, 1.3 Hz, 1H, ind. C_4_-H), 7.67 (s, 1H, b.t. N-H); **^13^C NMR (APT)** (126 MHz, DMSO-*d6*) **δ** (ppm): 18.79 (ind. C_7_-CH_3_), 29.81 (ind. C_1_-CH_3_), 74.64 (spiro C), 103.97 (q, *J*= 3.8 Hz, b.t. C_4_), 115.82 (q, *J*= 4.2 Hz, b.t. C_6_), 121.03 (ind. C_7_), 121.81 (b.t. C_7_), 123.69 (ind. C_4_), 123.92 (ind. C_5_) 124.92 (q, *J*= 272.3 Hz, CF_3_), 127.13 (q, *J*= 31.4 Hz, b.t. C_5_), 129.74 (ind. C_3a_), 130.25 (b.t. C_7a_), 134.83 (ind. C_6_), 140.92 (ind. C_7a_), 148.22 (b.t. C_3a_), 175.20 (ind. C_2_); **LC-MS (ESI+):** 373.1 ([M+Na]^+^; 100), 351.0, ([M+H]^+^, 70); **Analyses (%) cald for C_17_H_13_F_3_N_2_OS:** C, 58.28; H, 3.74; N, 8.00; S, 9.15. Found: C, 57.79; H, 3.71; N, 8.05; S, 9.03. HPLC purity: 99.2%

***7'-Fluoro-1'-methyl-5-(trifluoromethyl)-3H-spiro[benzo[d]thiazole-2,3'-indolin]-2'-one (5j)***

White powder yield %95), molecular mass 354.32 g/mol; melting point 204-205°C; **IR (KBr) cm^-1^**: 3325 (N-H), 3099 (aromatic C-H), 2953 (aliphatic C-H), 1713 (C=O) 1645, 1624, 1489 (C=C); **^1^H-NMR** (DMSO-*d_6_*, 400 MHz) **δ** (ppm): 3.27 (d, 3H, ind. C_1_-CH_3_), 6.76 (d, *J* = 1.7 Hz, 1H, b.t. C_4_-H), 6.95 (dd, *J* = 7.9, 1.8 Hz, 1H, b.t. C_6_-H ), 7.13 (td, *J* = 8.0, 4.4 Hz, 1H, ind. C_5_-H), 7.27 (d, *J* = 8.0 Hz, 1H, b.t. C_6_-H), 7.30 (d, *J* = 8.4 Hz, 1H, ind. C_6_-H), 7.47 (d, *J* = 7.4, 1H, ind. C_4_-H), 7.68 (s, 1H, b.t. N-H); **^13^C NMR (APT)** (126 MHz, DMSO-*d6*) **δ** (ppm): 29.18 (d, *J* = 5.5 Hz, ind. C_1_-CH_3_), 74.89 (d, *J* = 2.9 Hz, spiro C), 104.11 (q, *J*= 3.8 Hz, b.t. C4), 115.96 (q, *J*= 4.13 Hz, b.t. C6), 119.04 (d, *J*= 19.0 Hz, ind. C_6_), 121.87 (b.t. C_7_), 122.14 (d, *J*= 3.1 Hz, ind. C_4_), 124.66 (d, *J*= 6.3, ind. C_5_), 124.90 (q, *J*= 271.6 Hz, CF_3_), 127.20 (q, *J*= 31.6 Hz, b.t. C_5_), 129.74 (d, *J*= 8,7 Hz, ind. C_7a_), 129.90 (b.t. C_7a_), 132.29 (d, *J*= 2.9 Hz, ind. C_3a_), 147.34 (d, *J*= 244.13 Hz, ind. C_7_), 147.81 (b.t. C_3a_), 174.42 (ind. C_2_); **LC-MS (ESI+):** 377.0 ([M+Na]^+^; 100), 355.0, ([M+H]^+^, 42); **Analyses (%) cald for C_16_H_10_F_4_N_2_OS:** C, 54.24; H, 2.84; N, 7.91; S, 9.05. Found: C, 54.29; H, 2.88; N, 7.96; S, 9.06. HPLC purity: 100.0%

***7'-Chloro-1'-methyl-5-(trifluoromethyl)-3H-spiro[benzo[d]thiazole-2,3'-indolin]-2'-one (5k)***

Silvery white powder (yield %63), molecular mass 370.77 g/mol; melting point 215-217°C; **IR (KBr) cm^-1^**: 3325 (N-H), 3175, 3071 (aromatic C-H), 2955, 2887 (aliphatic C-H), 1711 (C=O) 1605, 1585, 1456 (C=C); **^1^H-NMR** (DMSO-*d_6_*, 400 MHz) **δ** (ppm): 3.43 (s, 3H, ind. C_1_-CH_3_), 6.77 (d, *J* = 1.8 Hz, 1H, b.t. C_4_-H), 6.96 (d, *J* = 8.0 Hz, 1H, b.t. C_6_-H), 7.14 (t, *J* = 7.8 Hz, 1H, ind. C_5_-H), 7.28 (d, 1H, *J* = 7.8 H, b.t. C_7_-H), 7.41 (d, *J* = 8.1, 1H, ind. C_6_-H), 7.62 (d, 1H, *J* = 7.4 Hz, ind. C_4_-H), 7.66 (s, 1H, b.t. N-H); **^13^C NMR (APT)** (126 MHz, DMSO-*d6*) **δ** (ppm): 29.58 (ind. C_1_-CH_3_), 74.10 (spiro C), 103.72 (q, *J*= 3.8 Hz, b.t. C_4_), 114.78 (ind. C_7_), 115.57 (q, *J*= 3.8 Hz, b.t. C_6_), 121.47 (b.t. C_7_), 124.49 (q, *J*= 272.5 Hz, CF_3_), 124.61 (ind. C_4_), 124.67 (ind. C_5_), 126.80 (q, *J*= 32.4 Hz, b.t. C_5_), 129.44 (ind. C_3a_), 132.14 (b.t. C_7a_), 132.70 (ind. C_6_), 138.55 (ind. C_7a_), 147.35 (b.t. C_3a_), 174.58 (ind. C_2_); **LC-MS (ESI+):** 393.0, 395.0 ([M+Na]^+^; 100, 38), 371.0, 373.0 ([M+H]^+^, 30, 10); **Analyses (%) cald for C_16_H_10_ClF_3_N_2_OS:** C, 51.83; H, 2.72; N, 7.56; S, 8.65. Found: C, 51.63; H, 2.72; N, 7.62; S, 8.62. HPLC purity: 99.7%

***7'-Bromo-1'-methyl-5-(trifluoromethyl)-3H-spiro[1,3-benzothiazole-2,3'-indol]-2'-on (5l)***

Silvery white powder (yield %63), molecular mass 415.22 g/mol; melting point 218-221°C; **IR (KBr) cm^-1^**: 3327 (N-H), 3067, 3019 (aromatic C-H), 2953 (aliphatic C-H), 1707 (C=O) 1604, 1578, 1454 (C=C); **^1^H-NMR** (DMSO-*d_6_*, 400 MHz) **δ** (ppm): 3.45 (s, 3H, ind. C_1_-CH_3_), 6.77 (d, *J* = 1.8 Hz, 1H, b.t. C_4_-H), 6.96 (d, *J* = 8.0 Hz, 1H, b.t. C_6_-H ), 7.07 (t, *J* = 7.8 Hz, 1H, ind. C_5_-H), 7.28 (d, 1H, *J* = 7.9 H, b.t. C_7_-H), 7.56 (d, *J* = 8.1, 1H, ind. C_6_-H), 7.64 – 7.66 (m, 2H, ind. C_4_-H, b.t. N-H); **^13^C NMR (APT)** (126 MHz, DMSO-*d6*) **δ** (ppm): 29.77 (ind. C_1_-CH_3_), 74.07 (spiro C), 102.03 (ind. C_7_), 103.70 (q, *J*= 3.8 Hz, b.t. C_4_), 115.57 (q, *J*= 4.1 Hz, b.t. C_6_), 121.47 (b.t. C_7_), 124.96 (q, *J*= 272.5 Hz, CF_3_), 125.0 (ind. C_4_), 125.15 (ind. C_5_), 127.30 (q, *J*= 31.5 Hz, b.t. C_5_), 129.35 (b.t. C_7a_), 132.45 (ind. C_3a_), 136.02 (ind. C_6_), 139.97 (ind. C_7a_), 147.33 (b.t. C_3a_), 174.73 (ind. C_2_). **LC-MS (ESI+):** 437.0, 439.0 ([M+Na]^+^; 30, 32), 416.0, 418.0 ([M+H]^+^, 10, 8), 311.2 (100); **Analyses (%) cald for C_16_H_10_BrF_3_N_2_OS:** C, 46.28; H, 2.43; N, 6.75; S, 7.72. Found: C, 46.13; H, 2.47; N, 6.84; S, 7.71. HPLC purity: 97.8%

***5'-chloro-1',7'-dimethyl-5-(trifluoromethyl)-3H-spiro[1,3-benzothiazole-2,3'-indol]-2'-on (5m)***

Yellow powder (yield %77), molecular mass 384.80 g/mol; melting point 236-238°C; **IR (KBr) cm^-1^**: 3337 (N-H), 3064, 3044 (aromatic C-H), 2936, 2870 (aliphatic C-H), 1705 (C=O) 1599, 1581, 1458 (C=C); **^1^H-NMR** (DMSO-*d_6_*, 600 MHz) **δ** (ppm): 2.55 (s, 3H, ind. C_7_-CH_3_), 3.39 (s, 3H, ind. N-CH_3_), 6.77 (d, *J* = 1.8 Hz, 1H, b.t. C_4_-H), 6.98 (dd, *J* = 8.0, 1.8 Hz, 1H, b.t. C_6_-H), 7.28 (d, *J* = 8 Hz, b.t. C_7_-H ), 7.29 (d, *J* = 2.2 Hz, 1H, ind. C_6_-H), 7.50 (d, *J* = 2.2 Hz, 1H, ind. C_4_-H), 7.69 (s, 1H, b.t. N-H); **^13^C NMR (APT)** (126 MHz, DMSO-*d_6_*) **δ** (ppm): 18.44 (ind. C_7_-CH_3_), 29.81 (ind. N-CH_3_), 74.48 (spiro C), 104.21 (q, *J*= 3.6 Hz, b.t. C_4_), 116.01 (q, *J*= 4.1 Hz, b.t. C_6_), 121.88 (b.t. C_7_), 123.42 (ind. C_7_), 123.58 (ind. C_4_), 124.94 (q, *J*= 272.2 Hz, CF_3_), 127.22 (q, *J*= 31.4 Hz, b.t. C_5_), 127.33 (ind. C_5_), 129.98 (b.t. C_7a_), 131.90 (ind. C_3a_), 134.00 (ind. C_6_), 139.93 (ind. C_7a_), 147.94 (b.t. C_3a_), 175.02 (ind. C_2_); **LC-MS (ESI+):** 407.0, 409.0 ([M+Na]^+^; 100, 38), 385.0, 387.0 ([M+H]^+^, 38, 13); **Analyses (%) cald for C_17_H_12_ClF_3_N_2_OS:** C, 53.06; H, 3.14; N, 7.28; S, 8.27. Found: C, 52.83; H, 3.11; N, 7.43; S, 8.27. HPLC purity: 95.6%

***5',7'-Dichloro-1'-methyl-5-(trifluoromethyl)-3H-spiro[benzo[d]thiazole-2,3'-indolin]-2'-one (5n)***

Silvery white powder (yield %48), molecular mass 405.22 g/mol; melting point 196-197°C; **IR (KBr) cm^-1^**: 3323 (N-H), 3092 (aromatic C-H), 2953 (aliphatic C-H), 1711 (C=O) 1606, 1578, 1458 (C=C); **^1^H-NMR** (DMSO-*d_6_*, 400 MHz) **δ** (ppm): 3.44 (s, 3H, ind. C_1_-CH_3_), 6.82 (d, *J* = 1.7 Hz, 1H, b.t. C_4_-H), 6.99 (dd, *J* = 8.1, 1.8 Hz, 1H, b.t. C_6_-H), 7.31 (d, 1H, *J* = 7.9 H, b.t. C_7_-H), 7.60 (d, *J* = 2.1, 1H, ind. C_6_-H), 7.66 (s, 1H, b.t. N-H), 7.68 (d, *J =* 2.1 Hz, ind. C_4_-H); **^13^C NMR (APT)** (126 MHz, DMSO-*d_6_*) **δ** (ppm): 30.04 (ind. C_1_-CH_3_), 74.51 (spiro C), 104.38 (q, *J*= 4.1 Hz, b.t. C_4_), 116.02 (ind. C_7_), 116.15 (q, *J*= 4.2 Hz, b.t. C_6_), 121.92 (b.t. C_7_), 124.56 (q, *J*= 271.1 Hz, CF_3_), 125.14 (ind. C_4_), 127.31 (q, *J*= 31.5 Hz, b.t. C_5_), 128.08 (ind. C_5_), 129.72 (b.t. C_7a_), 132.18 (ind. C_6_), 134.34 (ind. C_3a_), 138.27 (ind. C_7a_), 147.52 (b.t. C_3a_), 174.84 (ind. C_2_); **LC-MS (ESI+):** 427.0, 429.0, 431.0 ([M+Na]^+^; 30, 28, 5), 405.0, 407.0, 409.0 ([M+H]^+^, 20, 18, 6), 249.1 (100); **Analyses (%) cald for C_16_H_9_Cl_2_F_3_N_2_OS.^2/10^H_2_O:** C, 47.01; H, 2.32; N, 6.85; S, 7.84. Found: C, 46.60; H, 2.24; N, 7.02; S, 7.80. HPLC purity: 99.3%

**Spectrums**

***5'-Bromo-5-(trifluoromethyl)-3H-spiro[1,3-benzothiazole-2,3’-indol]-2’(1'H)-one (4a)***

Figure S- 1: IR spectrum of **4a**

Figure S- 2: ^1^H-NMR spectrum of compound **4a** ( δ 0-14 ppm)

Figure S- 3: ^1^H-NMR spectrum of compound **4a** ( δ 6.6-8.0 ppm)

Figure S- 4: ^13^C-NMR APT spectrum of compound **4a** ( δ 0-200 ppm)

Figure S- 5: LC-MS spectrum of compound **4a**

Figure S- 6: HPLC chromatogram of compound **4a**

***5'-Iodo-5-(trifluoromethyl)-3H-spiro[1,3-benzothiazole-2,3’-indol]-2’(1'H)-one (4b)***

Figure S- 7: IR spectrum of compound **4b**

Figure S- 8: ^1^H-NMR spectrum of compound **4b** ( δ 0-14 ppm)

Figure S- 9: ^1^H-NMR spectrum of compound **4b** ( δ 6.6-8.0 ppm)

Figure S- 10: ^13^C-NMR APT spectrum of compound **4b** ( δ 0-200 ppm)

Figure S- 11: LC-MS spectrum of compound **4b**

Figure S- 12: HPLC chromatogram of compound **4b**

***7'-Methyl-5-(trifluoromethyl)-3H-spiro[1,3-benzothiazole-2,3’-indol]-2’(1'H)-one (4c)***

Figure S- 13: IR spectrum of compound **4c**

Figure S- 14: ^1^H-NMR spectrum of compound **4c** ( δ 0-14 ppm)

Figure S- 15: ^1^H-NMR spectrum of compound **4c** ( δ 6.4-8.2 ppm)

Figure S- 16: ^13^C-NMR APT spectrum of compound **4c** ( δ 0-200 ppm)

Figure S- 17: LC-MS spectrum of compound **4c**

Figure S- 18: HPLC chromatogram of compound **4c**

***7'-Fluoro-5-(trifluoromethyl)-3H-spiro[1,3-benzothiazole-2,3’-indol]-2’(1'H)-one (4d)***

Figure S- 19: IR spectrum of compound **4d**

Figure S- 20: ^1^H-NMR spectrum of compound **4d** ( δ 0-14 ppm)

Figure S- 21: ^1^H-NMR spectrum of compound **4d** ( δ 6.6-7.6 ppm)

Figure S- 22: ^13^C-NMR APT spectrum of compound **4d** ( δ 0-180 ppm)

Figure S- 23: LC-MS spectrum of compound **4d**

Figure S- 24: HPLC chromatogram of compound **4d**

***7'-Chloro-5-(trifluoromethyl)-3H-spiro[1,3-benzothiazole-2,3’-indol]-2’(1'H)-one (4e)***

Figure S- 25: R spectrum of compound **4e**

Figure S- 26: ^1^H-NMR spectrum of compound **4e** ( δ 0-14 ppm)

Figure S- 27: ^1^H-NMR spectrum of compound **4e** ( δ 6.6-8.0 ppm)

Figure S- 28: ^13^C-NMR APT spectrum of compound **4e** ( δ 0-190 ppm)

Figure S- 29: ^13^C-NMR APT spectrum of compound **4e** ( δ 0-190 ppm)

Figure S- 30: HPLC chromatogram of compound **4e**

***7'-Bromo-5-(trifluoromethyl)-3H-spiro[1,3-benzothiazole-2,3’-indol]-2’(1'H)-one (4f)***

Figure S- 31: IR spectrum of compound **4f**

Figure S- 32: ^1^H-NMR spectrum of compound **4f** ( δ 0-12.0 ppm)

Figure S- 33: ^1^H-NMR spectrum of compound **4f** ( δ 6.6-8.0 ppm)

Figure S- 34: ^13^C-NMR APT spectrum of compound **4f** ( δ 126-136 ppm)

Figure S- 35: ^13^C-NMR APT spectrum of compound **4f** ( spiro C signal)

Figure S- 36: HMBC-2D spectrum of compound **4f** ( δ 6.0-11.0 ppm)

Figure S- 37: HMBC-2D spectrum of compound **4f** ( δ 6.7-8.0 ppm)

Figure S- 38: LC-MS spectrum of compound **4f**

Figure S- 39: HPLC chromatogram of compound **4f**

***5'-Chloro-7’-methyl-5-(trifluoromethyl)-3H-spiro[1,3-benzothiazole-2,3’-indol]-2’(1'H)-one (4g)***

Figure S- 40: IR spectrum of compound **4g**

Figure S- 41: ^1^H-NMR spectrum of compound **4g** ( δ 0-14 ppm)

Figure S- 42: ^1^H-NMR spectrum of compound **4g** ( δ 6.6-8.0 ppm)

Figure S- 43: ^13^C-NMR APT spectrum of compound **4g** ( δ 0-200 ppm)

Figure S- 44: LC-MS spectrum of compound **4g**

Figure S- 45: HPLC chromatogram of compound **4g**

***5',7’dichloro -5-(trifluoromethyl)-3H-spiro[1,3-benzothiazole-2,3’-indol]-2’(1'H)-one (4h)***

Figure S- 46: R spectrum of compound **4h**

Figure S- 47: ^1^H-NMR spectrum of compound **4h** ( δ 0-14.0 ppm)

Figure S- 48: ^1^H-NMR spectrum of compound **4h** ( δ 6.6-8.0 ppm)

Figure S- 49: C-NMR APT spectrum of compound **4h** ( δ 0-180 ppm)

Figure S- 50: 43.LC-MS spectrum of compound **4h**

Figure S- 51: HPLC chromatogram of compound **4h**

***5-Trifluoromethyl-3H-spiro[benzo[d]thiazole-2,3’-indolin]-2’one (4i)***

Figure S- 52: IR spectrum of compound **4i**

Figure S- 53: ^1^H-NMR spectrum of compound **4i**

Figure S- 54: ^13^C-NMR (decoupled) spectrum of compound **4i**

Figure S- 55: LC-MS spectrum of compound **4i**

Figure S- 56: HPLC chromatogram of compound **4i**

***5'-Methyl-5-(trifluoromethyl)-3H-spiro[benzo[d]thiazole-2,3'-indolin]-2'-one (4j)***

Figure S- 57: IR spectrum of compound **4j**

DMSO

H_2_O

Figure S- 58: ^1^H-NMR spectrum of compound **4j** (δ 0.0-11.0 ppm)

Figure S- 59: ^1^H-NMR spectrum of compound **4j** (δ 6.3-7.9 ppm)

DMSO

Figure S- 60: ^13^C-NMR APT spectrum of compound **4j**

Figure S- 61: HSQC-2D spectrum of compound **4j** (δ 6.5-7.9 / 100-136 ppm)

Figure S- 62: HSQC-2D spectrum of compound **4j** (δ 2.05-2.75 / 18-42 ppm)

Figure S- 63: LC-MS spectrum of compound **4j**

Figure S- 64: HPLC chromatogram of compound **4j**

***5'-Trifluoromehtoxy-5-(trifluoromethyl)-3H-spiro[benzo[d]thiazole-2,3'-indolin]-2'-one (4k)***

Figure S- 65: IR spectrum of compound **4k**

H_2_O

DMSO

Figure S- 66: ^1^H-NMR spectrum of compound **4k** (δ 0.0-11.0 ppm)

Figure S- 67: ^1^H-NMR spectrum of compound **4k** (δ 6.7-8.1 ppm)

DMSO

Figure S- 68: ^13^C-NMR APT spectrum of compound **4k**

Figure S- 69: HSQC-2D spectrum of compound **4k** (δ 6.4-9.3 / 85-140 ppm)

Figure S- 70: HSQC-2D spectrum of compound **4k** (δ 6.6-7.8 / 96-133 ppm)

Figure S- 71: LC-MS spectrum of compound **4k**

Figure S- 72: HPLC chromatogram of compound **4k**

***5'-Fluoro-5-(trifluoromethyl)-3H-spiro[benzo[d]thiazole-2,3'-indolin]-2'-one (4l)***

Figure S- 73: IR spectrum of compound **4l**

Figure S- 74: ^1^H-NMR spectrum of compound **4l**

Figure S- 75: ^13^C-NMR APT spectrum of compound **4l**

Figure S- 76: LC-MS spectrum of compound **4l**

Figure S- 77: HPLC chromatogram of compound **4l**

***5'-Chloro-5-(trifluoromethyl)-3H-spiro[benzo[d]thiazole-2,3'-indolin]-2'-one (4m)***

Figure S- 78: IR spectrum of compound **4m**

DMSO

H_2_O

Figure S- 79: ^1^H-NMR spectrum of compound **4m** (δ 0.0-11.0 ppm)

Figure S- 80: ^1^H-NMR spectrum of compound **4m** (δ 6.7-7.8 ppm)

Figure S- 81: ^13^C-NMR APT spectrum of compound **4m**

Figure S- 82: LC-MS spectrum of compound **4m**

Figure S- 83: : HPLC chromatogram of compound **4m**

***1'-Methyl-5-(trifluoromethyl)-3H-******spiro[benzo[d]thiazole-2,3'-indolin]-2'-one (5a)***

Figure S- 84: IR spectrum of compound **5a**

Figure S- 85: ^1^H-NMR spectrum of compound **5a**

Figure S- 86: ^13^C-NMR APT spectrum of compound **5a**

Figure S- 87: LC-MS spectrum of compound **5a**

Figure S- 88: HPLC chromatogram of compound **5a**

***1',5'-Dimethyl-5-(trifluoromethyl)-3H-spiro[benzo[d]thiazole-2,3'-indolin]-2'-one (5b)***

Figure S- 89: IR spectrum of compound **5b**

Figure S- 90: ^1^H-NMR spectrum of compound **5b**

Figure S- 91: ^13^C-NMR APT spectrum of compound **5b**

Figure S- 92: LC-MS spectrum of compound **5b**

Figure S- 93: HPLC chromatogram of compound **5b**

***1'-Methyl-5'-(trifluoromethoxy)-5-(trifluoromethyl)-3H-spiro[benzo[d]thiazole-2,3'-indolin]-2'-one (5c)***

Figure S- 94: IR spectrum of compound **5c**

Figure S- 95: ^1^H-NMR spectrum of compound **5c**

Figure S- 96: ^13^C-NMR APT spectrum of compound **5c**

Figure S- 97: HMBC-2D spectrum of compound **5c** (δ 0-10.0 / 0-200 ppm)

Figure S- 98: HMBC-2D spectrum of compound **5c** (δ 6.40-8.0 / 70-150 ppm)

Figure S- 99: HMBC-2D spectrum of compound **5c** (δ 6.80-7.65 / 110-150 ppm)

Figure S- 100: LC-MS spectrum of compound **5c**

Figure S- 101: HPLC chromatogram of compound **5c**

***5'-Fluoro-1'-methyl-5-(trifluoromethyl)-3H-spiro[benzo[d]thiazole-2,3'-indolin]-2'-one (5d)***

Figure S- 102: IR spectrum of compound **5d**

Figure S- 103: ^1^H-NMR spectrum of compound **5d**

DMSO

Figure S- 104: ^13^C-NMR APT spectrum of compound **5d**

Figure S- 105: LC-MS spectrum of compound **5d**

Figure S- 106: HPLC chromatogram of compound **5d**

***5'-Chloro-1'-methyl-5-(trifluoromethyl)-3H-spiro[benzo[d]thiazole-2,3'-indolin]-2'-one (5e)***

Figure S- 107: IR spectrum of compound **5e**

Figure S- 108: ^1^H-NMR spectrum of compound **5e**

Figure S- 109: ^13^C-NMR APT spectrum of compound **5e**

Figure S- 110: HSQC-2D spectrum of compound **5e** (δ 2.0-8.5 / 15-150 ppm)

Figure S- 111: LC-MS spectrum of compound **5e**

Figure S- 112: HPLC chromatogram of compound **5e**

***5'-Bromo-1'-methyl-5-(trifluoromethyl)-3H-spiro[benzo[d]thiazole-2,3'-indolin]-2'-one (5f)***

Figure S- 113: IR spectrum of compound **5f**

Figure S- 114: ^1^H-NMR spectrum of compound **5f**

Figure S- 115: ^13^C-NMR APT spectrum of compound **5f**

Figure S- 116: LC-MS spectrum of compound **5f**

Figure S- 117: HPLC chromatogram of compound **5f**

***5'-Iodo-1'-methyl-5-(trifluoromethyl)-3H-spiro[benzo[d]thiazole-2,3'-indolin]-2'-one (5g)***

Figure S- 118: IR spectrum of compound **5g**

Figure S- 119: ^1^H-NMR spectrum of compound **5g**

Figure S- 120: ^13^C-NMR APT spectrum of compound **5g**

Figure S- 121: LC-MS spectrum of compound **5g**

Figure S- 122: HPLC chromatogram of compound **5g**

***1'-Methyl-5'-nitro-5-(trifluoromethyl)-3H-spiro[benzo[d]thiazole-2,3'-indolin]-2'-one (5h)***

Figure S- 123: IR spectrum of compound **5h**

Figure S- 124: ^1^H-NMR spectrum of compound **5h**

Figure S- 125: ^13^C-NMR APT spectrum of compound **5h**

Figure S- 126: LC-MS spectrum of compound **5h**

Figure S- 127: HPLC chromatogram of compound **5h**

***1',7'-Dimethyl-5-(trifluoromethyl)-3H-spiro[benzo[d]thiazole-2,3'-indolin]-2'-one (5i)***

Figure S- 128: IR spectrum of compound **5i**

Figure S- 129: ^1^H-NMR spectrum of compound **5i**

Figure S- 130: ^13^C-NMR APT spectrum of compound **5i**

Figure S- 131: LC-MS spectrum of compound **5i**

Figure S- 132: HPLC chromatogram of compound **5i**

***7'-Fluoro-1'-methyl-5-(trifluoromethyl)-3H-spiro[benzo[d]thiazole-2,3'-indolin]-2'-one (5j)***

Figure S- 133: ^1^H-NMR spectrum of compound **5j**

Figure S- 134: ^13^C-NMR APT spectrum of compound **5j**

Figure S- 135: LC-MS spectrum of compound **5j**

Figure S- 136: HPLC chromatogram of compound **5j**

***7'-Chloro-1'-methyl-5-(trifluoromethyl)-3H-spiro[benzo[d]thiazole-2,3'-indolin]-2'-one (5k)***

Figure S- 137: ^1^H-NMR APT spectrum of compound **5k**

Figure S- 138: ^13^C-NMR APT spectrum of compound **5k**

Figure S- 139: LC-MS spectrum of compound **5k**

Figure S- 140: HPLC chromatogram of compound **5k**

***7'-Bromo-1'-methyl-5-(trifluoromethyl)-3H-spiro[1,3-benzothiazole-2,3'-indol]-2'-on (5l)***

Figure S- 141: ^1^H-NMR spectrum of compound **5l**

Figure S- 142: ^13^C-NMR APT spectrum of compound **5l**

Figure S- 143: LC-MS spectrum of compound **5l**

Figure S- 144: HPLC chromatogram of compound **5l**

***5'-Chloro-1',7'-dimethyl-5-(trifluoromethyl)-3H-spiro[benzo[d]thiazole-2,3'-indolin]-2'-one (5m)***

Figure S- 145: IR spectrum of compound **5m**

Figure S- 146: ^1^H-NMR spectrum of compound **5m**

Figure S- 147: ^13^C-NMR APT spectrum of compound **5m**

Figure S- 148: LC-MS spectrum of compound **5m**

Figure S- 149: HPLC chromatogram of compound **5m**

***5',7'-Dichloro-1'-methyl-5-(trifluoromethyl)-3H-spiro[benzo[d]thiazole-2,3'-indolin]-2'-one (5n)***

Figure S- 150: IR spectrum of compound **5n**

Figure S- 151: ^1^H-NMR APT spectrum of compound **5n**

Figure S- 152: ^13^C-NMR APT spectrum of compound **5n**

Figure S- 153: HMBC-2D spectrum of compound **5n** (δ 2.2-7.8 / 35-175 ppm)

Figure S- 154: HMBC-2D spectrum of compound **5n** (δ 6.8-7.9 / 65-150 ppm)

Figure S- 155: LC-MS spectrum of compound **5n**

Figure S- 156: HPLC chromatogram of compound **5n**

**Tables**

Tablo S 1. In silico physicochemical properties of compounds **4a-m** and **5a-n**

| **Comp.** | **MW** | **HBD** | **HBA** | **QPlogP octanol / water** | **Violence of RO5** | **QPlogS** | **QPlogBB** | **QPlogKhsa** | **HOA** |
| --- | --- | --- | --- | --- | --- | --- | --- | --- | --- |
|  | **Ref.**  **<500** | **<5** | **<10** | **<5** | **≤1** | **-6.5 to 0.5** | **-3 to 1.2** | **-1.5 to 1.5** | **>80 high;**  **<25 weak** |
| **4a** | 322.304 | 2.000 | 4.000 | 2.682 | 0 | -3.705 | 0.483 | 0.194 | 87.748 |
| **4b** | 336.331 | 2.000 | 4.000 | 2.939 | 0 | -4.166 | 0.446 | 0.336 | 88.847 |
| **4c** | 406.302 | 2.000 | 4.000 | 3.632 | 0 | -4.748 | 0.663 | 0.413 | 92.967 |
| **4d** | 340.295 | 2.000 | 4.000 | 2.879 | 0 | -3.986 | 0.583 | 0.230 | 88.550 |
| **4e** | 356.749 | 2.000 | 4.000 | 3.135 | 0 | -4.360 | 0.626 | 0.300 | 90.000 |
| **4f** | 401.200 | 2.000 | 4.000 | 3.207 | 0 | -4.464 | 0.635 | 0.321 | 90.393 |
| **4g** | 448.201 | 2.000 | 4.000 | 3.293 | 0 | -4.590 | 0.645 | 0.346 | 90.909 |
| **4h** | 336.331 | 2.000 | 4.000 | 2.968 | 0 | -4.405 | 0.374 | 0.351 | 88.350 |
| **4i** | 340.295 | 2.000 | 4.000 | 2.940 | 0 | -4.226 | 0.554 | 0.241 | 88.845 |
| **4j** | 356.749 | 2.000 | 4.000 | 3.156 | 0 | -4.550 | 0.568 | 0.311 | 89.463 |
| **4k** | 401.200 | 2.000 | 4.000 | 3.227 | 0 | -4.655 | 0.576 | 0.332 | 89.871 |
| **4l** | 370.776 | 2.000 | 4.000 | 3.458 | 0 | -5.139 | 0.527 | 0.462 | 91.222 |
| **4m** | 391.194 | 2.000 | 4.000 | 3.662 | 0 | -5.322 | 0.732 | 0.419 | 92.793 |
| **5a** | 336.331 | 1.000 | 4.500 | 3.214 | 0 | -4.006 | 0.808 | 0.300 | 96.831 |
| **5b** | 350.358 | 1.000 | 4.500 | 3.513 | 0 | -4.556 | 0.792 | 0.451 | 100.000 |
| **5c** | 420.329 | 1.000 | 4.500 | 4.209 | 0 | -5.146 | 1.006 | 0.531 | 100.000 |
| **5d** | 354.321 | 1.000 | 4.500 | 3.447 | 0 | -4.364 | 0.921 | 0.339 | 100.000 |
| **5e** | 370.776 | 1.000 | 4.500 | 3.707 | 0 | -4.747 | 0.970 | 0.413 | 100.000 |
| **5f** | 415.227 | 1.000 | 4.500 | 3.780 | 0 | -4.854 | 0.980 | 0.436 | 100.000 |
| **5g** | 462.228 | 1.000 | 4.500 | 3.867 | 0 | -4.983 | 0.992 | 0.462 | 100.000 |
| **5h** | 381.329 | 1.000 | 5.500 | 2.527 | 0 | -4.275 | -0.277 | 0.277 | 75.157 |
| **5i** | 350.358 | 1.000 | 4.500 | 3.403 | 0 | -4.202 | 0.814 | 0.399 | 100.000 |
| **5j** | 354.321 | 1.000 | 4.500 | 3.392 | 0 | -4.271 | 0.893 | 0.327 | 100.000 |
| **5k** | 370.776 | 1.000 | 4.500 | 3.566 | 0 | -4.460 | 0.928 | 0.380 | 100.000 |
| **5l** | 415.227 | 1.000 | 4.500 | 3.624 | 0 | -4.529 | 0.941 | 0.396 | 100.000 |
| **5m** | 384.803 | 1.000 | 4.500 | 3.913 | 0 | -5.030 | 0.968 | 0.517 | 100.000 |
| **5n** | 405.221 | 1.000 | 4.500 | 4.077 | 0 | -5.244 | 1.104 | 0.493 | 100.000 |

**Abbreviation: QPlogP:** Partition coefficient**, QPlogS:** Solubility in water**, QPlogBB:** Blood-brain barrier partition coefficient**, QPlogKhsa:** Human serum albumin binding ratio**, HOA:** Oral absorption percentage in human.

CCDC-2504583 contains the supplementary crystallographic data for **5h** reported in this paper. These data can be obtained free of charge at [www.ccdc.cam.ac.uk/conts/](http://www.ccdc.cam.ac.uk/conts/) retrieving.html [or from the Cambridge Crystallographic Data Centre (CCDC), 12 Union Road, Cambridge CB2 1EZ, UK; fax: +44(0)1223 336033; e-mail: [deposit@ccdc.cam.ac.uk](mailto:deposit@ccdc.cam.ac.uk)]. Supplementary data associated with this article can be found in the online version at <http://dx.doi.org/10.1016/j.saa.2013.08.054>.

**Checkcif Reports of compound 5h**

Figure S- 157: Conformational superposition of the experimental X-ray crystal structure and the predicted lowest-energy conformation of the title compound (RMSD = 1.5670 Å)
