# Supplementary material for: Spirooxindole 5‐(Trifluoromethyl)benzothiazole Hybrids: Synthesis, Crystal Structure, and Antioxidant Activity
Source: Arch Pharm (Weinheim). 2026 Sep 28;359(9):e70343. doi: 10.1002/ardp.70343 (PMC13617587; doi:10.1002/ardp.70343)
Supplement: Supplementary file 2 [file ARDP-359-e70343-s001.doc]

**Supplemental Material: Novel Compounds and Biological Screening Results**

Spirooxindole-benzothiazole hybrids bearing a trifluoromethyl moiety: Synthesis, crystal structure and antioxidant activity.

Nida Karabağ^1,2^, Asu Busra Temizer Keser^2,3*^, Murat Pekmez^4^, Elif Mertoğlu-Kamalı^4^, Filiz Betül Kaynak^5^, [Nurten Özsoy](javascript:__doPostBack('ctl00$CPHContent$projeKart$TabContainer1$tp1$rptArastirmaci$ctl01$imgElaman',''))^6^, Nazlı Arda^4^, Nilgün Karalı^1^

^1^Department of Pharmaceutical Chemistry, Faculty of Pharmacy, Istanbul University, Istanbul, Turkey

^2^Department of Pharmaceutical Chemistry, Institute of Health Sciences, Istanbul University, Istanbul, Turkey

^3^Department of Pharmaceutical Chemistry, Faculty of Pharmacy, Istanbul University-Cerrahpaşa, Istanbul, Turkey

^4^Department of Molecular Biology and Genetics, Faculty of Science, Istanbul University, Istanbul, Turkey

^5^Department of Physical Engineering, Faculty of Engineering, Hacettepe University, Ankara, Turkey

^6^Department of Biochemistry, Faculty of Pharmacy, Istanbul University, Istanbul, Turkey

*Correspondence:

Asu Busra Temizer Keser

Email: [asu.temizer@iuc.edu.tr](mailto:asu.temizer@iuc.edu.tr)

Adress: İstanbul Üniversitesi-Cerrahpaşa, Faculty of Pharmacy, Department of Pharmaceutical Chemistry, Buyukcekmece Campus. Alkent 2000 34500 Buyukcekmece/İstanbul

|  |  | ***EC_50_ (µM/mL)** | | | ****(µM/L Troloks)** | *****(µM/L Fe^2+^)** | ******(activity, %)** | **IC_50_ (µM)** |
| --- | --- | --- | --- | --- | --- | --- | --- | --- |
| **Compound**  **No.** | **InChI** | **DPPH**^1^ | **ABTS**^2^ | **Superoxide**^3^ | **TEAC**^4^ | **FRAP**^5^ | **TBARS(Lipid peroxidation inhibiton, %)**^6^ | **Cytotoxic effects**^7^ |
| 1-4a | InChI=1S/C15H8BrF3N2OS/c16-8-2-3-10-9(6-8)14(13(22)20-10)21-11-5-7(15(17,18)19)1-4-12(11)23-14/h1-6,21H,(H,20,22) | 2,07 ± 0,06^b^ | 2,04 ± 0,07^b^ | 1,91 ± 0,02^b^ | 1.73 ± 0.02^a^ | 3.06 ± 0.06^b^ | 37.13±11.85 |  |
| 2-4b | InChI=1S/C15H8F3IN2OS/c16-15(17,18)7-1-4-12-11(5-7)21-14(23-12)9-6-8(19)2-3-10(9)20-13(14)22/h1-6,21H,(H,20,22) | 1,98 ± 0,04^b^ | 1,93 ± 0,25^b^ | 1,78 ± 0,03^b^ | 1.80 ± 0.02^a^ | 3.08 ± 0.07^b^ | 37.88±5.04 |  |
| 3-4c | InChI=1S/C16H11F3N2OS/c1-8-3-2-4-10-13(8)20-14(22)15(10)21-11-7-9(16(17,18)19)5-6-12(11)23-15/h2-7,21H,1H3,(H,20,22) | 2,29 ± 0,04^a^ | 2,37 ± 0,11^a^ | 2,17 ± 0,02^a^ | 1.68 ± 0.03^a^ | 2.49 ± 0.06^a^ | 36.59±7.85 |  |
| 4-4d | InChI=1S/C15H8F4N2OS/c16-9-3-1-2-8-12(9)20-13(22)14(8)21-10-6-7(15(17,18)19)4-5-11(10)23-14/h1-6,21H,(H,20,22) | 2,38 ± 0,01^a^ | 2,34 ± 0,09^a^ | 2,17 ± 0,03^a^ | 1.62 ± 0.01^a^ | 2.40 ± 0.09^a^ | 35.07±2.15 |  |
| 5-4e | InChI=1S/C15H8ClF3N2OS/c16-9-3-1-2-8-12(9)20-13(22)14(8)21-10-6-7(15(17,18)19)4-5-11(10)23-14/h1-6,21H,(H,20,22) | 2,28 ± 0,02^a^ | 2,23 ± 0,19^a^ | 2,18 ± 0,07^a^ | 1.63 ± 0.01^a^ | 2.53 ± 0.09^a^ | **53.09±2.97** |  |
| 6-4f | InChI=1S/C15H8BrF3N2OS/c16-9-3-1-2-8-12(9)20-13(22)14(8)21-10-6-7(15(17,18)19)4-5-11(10)23-14/h1-6,21H,(H,20,22) | 2,26 ± 0,09^a^ | 2,35 ± 0,07^a^ | 2,21 ± 0,01^a^ | 1.67 ± 0.02^a^ | 2.52 ± 0.04^a^ | **51.49±8.28** |  |
| 7-4g | InChI=1S/C16H10ClF3N2OS/c1-7-4-9(17)6-10-13(7)21-14(23)15(10)22-11-5-8(16(18,19)20)2-3-12(11)24-15/h2-6,22H,1H3,(H,21,23) | **1,16 ± 0,06^c^** | **1,04 ± 0,10^c^** | **1,09 ± 0,02^c^** | 1.90 ± 0.04^b^ | 3.03 ± 0.02^b^ | **45.81±4.33** | >200 |
| 8-4h | InChI=1S/C15H7Cl2F3N2OS/c16-7-4-8-12(9(17)5-7)21-13(23)14(8)22-10-3-6(15(18,19)20)1-2-11(10)24-14/h1-5,22H,(H,21,23) | **1,14 ± 0,02^c^** | **1,01 ± 0,08^c^** | **1,15 ± 0,03^c^** | 1.94 ± 0.01^b^ | 3.02 ± 0.09^b^ | **48.21±10.67** | 92.26±1.24 |
| 9-4i | InChI=1S/C15H9F3N2OS/c16-15(17,18)8-5-6-12-11(7-8)20-14(22-12)9-3-1-2-4-10(9)19-13(14)21/h1-7,20H,(H,19,21) | **1,01** **± 0,08^a,b^** | **0,97 ± 0,03^a,e^** | 1,19 ± 0,07^d^ | **1.23 ± 0.03^a,^**^e^ | 1.80 ± 0.09^a^ | **40.59±12.68** | 141.26±2.53 |
| 10-4j | InChI=1S/C16H11F3N2OS/c1-8-2-4-11-10(6-8)15(14(22)20-11)21-12-7-9(16(17,18)19)3-5-13(12)23-15/h2-7,21H,1H3,(H,20,22) | **1,05 ± 0,05^b^** | **1,17 ± 0,01^b,e^** | 1,35 ± 0,08^a^ | **1.13 ± 0.04^b^** | 1.76 ± 0.01^a^ | 38.83±12.14 | 70.79±1.13 |
| 11-4k | InChI=1S/C16H8F6N2O2S/c17-15(18,19)7-1-4-12-11(5-7)24-14(27-12)9-6-8(26-16(20,21)22)2-3-10(9)23-13(14)25/h1-6,24H,(H,23,25) | **1,21 ± 0,10^b,e^** | **1,15 ± 0,03^b,e,g^** | 1,33 ± 0,11^a^ | 1.26 ± 0.01^a,e,g^ | 1.24 ± 0.06^c^ | 39.36±11.92 |  |
| 12-4l | InChI=1S/C15H8F4N2OS/c16-8-2-3-10-9(6-8)14(13(22)20-10)21-11-5-7(15(17,18)19)1-4-12(11)23-14/h1-6,21H,(H,20,22) | **0,43 ± 0,05^c^** | **0,73 ± 0,04^c^** | **0,89 ± 0,07^b^** | 1.70 ± 0.02^c^ | 3.00 ± 0.09^b^ | **44.16±3.69** | 74.13±1.30 |
| 13-4m | InChI=1S/C15H8ClF3N2OS/c16-8-2-3-10-9(6-8)14(13(22)20-10)21-11-5-7(15(17,18)19)1-4-12(11)23-14/h1-6,21H,(H,20,22) | 1,58 ± 0,06^d^ | 2,13 ± 0,20^d^ | 1,76 ± 0,10^c^ | **0.85 ± 0.09^d^** | 1.16 ± 0.06^c^ | 34.41±7.38 |  |
| 14-5a | InChI=1S/C16H11F3N2OS/c1-21-12-5-3-2-4-10(12)15(14(21)22)20-11-8-9(16(17,18)19)6-7-13(11)23-15/h2-8,20H,1H3 | **0,92 ± 0,08^a,b^** | **1,10 ± 0,11^a,b,e^** | **1,06 ± 0,16^d^** | 1.33 ± 0.06^a,g^ | 1.32 ± 0.13^c^ | 29.03±6.49 | >200 |
| 15-5b | InChI=1S/C17H13F3N2OS/c1-9-3-5-13-11(7-9)16(15(23)22(13)2)21-12-8-10(17(18,19)20)4-6-14(12)24-16/h3-8,21H,1-2H3 | **1,15 ± 0,11^b,e^** | **1,22 ± 0,22^b,e,g^** | 1,40 ± 0,08^a^ | **1.27 ± 0.02^a,e,g^** | **0.91 ± 0.05^d^** | 24.50±5.78 | >200 |
| 16-5c | InChI=1S/C17H10F6N2O2S/c1-25-12-4-3-9(27-17(21,22)23)7-10(12)15(14(25)26)24-11-6-8(16(18,19)20)2-5-13(11)28-15/h2-7,24H,1H3 | 1,59 ± 0,03^d^ | 1,71 ± 0,06^d,f^ | 1,79 ± 0,06^c^ | **1.17 ± 0.02^e^** | **0.44 ± 0.02^h^** | 19.79±6.38 |  |
| 17-5d | InChI=1S/C16H10F4N2OS/c1-22-12-4-3-9(17)7-10(12)15(14(22)23)21-11-6-8(16(18,19)20)2-5-13(11)24-15/h2-7,21H,1H3 | 1,68 ± 0,09^d^ | 1,61 ± 0,13^f^ | 1,53 ± 0,05^e^ | **1.04 ± 0.10^e^** | **0.87 ± 0.22^d,e^** | 25.36±3.92 |  |
| 18-5e | InChI=1S/C16H10ClF3N2OS/c1-22-12-4-3-9(17)7-10(12)15(14(22)23)21-11-6-8(16(18,19)20)2-5-13(11)24-15/h2-7,21H,1H3 | 1,30 ± 0,10^e^ | 1,37 ± 0,18^e,f,g^ | 1,37 ± 0,07^a^ | **1.08 ± 0.08^e^** | **0.68 ± 0.04^e^** | 24.63±9.51 |  |
| 19-5f | InChI=1S/C16H10BrF3N2OS/c1-22-12-4-3-9(17)7-10(12)15(14(22)23)21-11-6-8(16(18,19)20)2-5-13(11)24-15/h2-7,21H,1H3 | 1,34 ± 0,10^e^ | 1,30 ± 0,16^e,f,g^ | 1,38 ± 0,05^a^ | **1.06 ± 0.09^e^** | **0.54 ± 0.04^f^** | 20.77±8.43 |  |
| 20-5g | InChI=1S/C16H10F3IN2OS/c1-22-12-4-3-9(20)7-10(12)15(14(22)23)21-11-6-8(16(17,18)19)2-5-13(11)24-15/h2-7,21H,1H3 | **1,23 ± 0,11^b,e^** | 1,49 ± 0,07^f^ | 1,42 ± 0,09^a^ | **1.06 ± 0.07^e^** | **0.46 ± 0.01^h^** | 19.97±9.46 |  |
| 21-5h | InChI=1S/C16H10F3N3O3S/c1-21-12-4-3-9(22(24)25)7-10(12)15(14(21)23)20-11-6-8(16(17,18)19)2-5-13(11)26-15/h2-7,20H,1H3 | **1,15 ± 0,06^b,e^** | 1,60 ± 0,11^f^ | 1,22 ± 0,05^d^ | **1.23 ± 0.03^a^** | **0.55 ± 0.01^f^** | 18.64±3.34 | >200 |
| 22-5i | InChI=1S/C17H13F3N2OS/c1-9-4-3-5-11-14(9)22(2)15(23)16(11)21-12-8-10(17(18,19)20)6-7-13(12)24-16/h3-8,21H,1-2H3 | 1,69 ± 0,04^d^ | 1,88 ± 0,08^d,f^ | 1,74 ± 0,05^c^ | **1.10 ± 0.03^e^** | **0.64 ± 0.02^e^** | 16.06±5.49 |  |
| 23-5j | InChI=1S/C16H10F4N2OS/c1-22-13-9(3-2-4-10(13)17)15(14(22)23)21-11-7-8(16(18,19)20)5-6-12(11)24-15/h2-7,21H,1H3 | 1,66 ± 0,13^d^ | 1,88 ± 0,17^d,f^ | 1,76 ± 0,08^c^ | **1.10 ± 0.06^e^** | **0.68 ± 0.01^e^** | 22.81±8.66 |  |
| 24-5k | InChI=1S/C16H10ClF3N2OS/c1-22-13-9(3-2-4-10(13)17)15(14(22)23)21-11-7-8(16(18,19)20)5-6-12(11)24-15/h2-7,21H,1H3 | 1,57 ± 0,03^d^ | 1,83 ± 0,05^d,f^ | 1,67 ± 0,06^c^ | **1.09 ± 0.05^e^** | **0.48 ± 0.04^f^** | 24.68±7.82 |  |
| 25-5l | InChI=1S/C16H10BrF3N2OS/c1-22-13-9(3-2-4-10(13)17)15(14(22)23)21-11-7-8(16(18,19)20)5-6-12(11)24-15/h2-7,21H,1H3 | 1,56 ± 0,08^d^ | 1,83 ± 0,02^d,f^ | 1,79 ± 0,04^c^ | **1.09 ± 0.01^e^** | **0.42 ± 0.01^h^** | 22.50±1.28 |  |
| 26-5m | InChI=1S/C17H12ClF3N2OS/c1-8-5-10(18)7-11-14(8)23(2)15(24)16(11)22-12-6-9(17(19,20)21)3-4-13(12)25-16/h3-7,22H,1-2H3 | 1,69 ± 0,03^d^ | 1,85 ± 0,05^d,f^ | 1,72 ± 0,06^c^ | **1.09 ± 0.03^e^** | **0.37 ± 0.01^j^** | 21.67±7.75 |  |
| 27-5n | InChI=1S/C16H9Cl2F3N2OS/c1-23-13-9(5-8(17)6-10(13)18)15(14(23)24)22-11-4-7(16(19,20)21)2-3-12(11)25-15/h2-6,22H,1H3 | 1,58 ± 0,04^d^ | 1,87 ± 0,16^d,f^ | 1,72 ± 0,05^c^ | **1.09 ± 0.06^e^** | **0.30** **± 0.02^k^** | **18.90±4.72** |  |
| Butylated hydroxytoluene (BHT) | InChI=1S/C15H24O/c1-10-8-11(14(2,3)4)13(16)12(9-10)15(5,6)7/h8-9,16H,1-7H3 | 1,20 ± 0,02^b,e^ | 1,28 ± 0,03^g^ | 1,18 ± 0,03^d^ | 1.30 ± 0.02^g^ | 0.98 ± 0.01^d^ | − |  |
| **α -Tocopherol** | InChI=1S/C29H50O2/c1-20(2)12-9-13-21(3)14-10-15-22(4)16-11-18-29(8)19-17-26-25(7)27(30)23(5)24(6)28(26)31-29/h20-22,30H,9-19H2,1-8H3 | 0,17 ± 0,01^f^ | 0,61 ± 0,01^h^ | 0,49 ± 0,04^f^ | 2.021 ± 0.01^f^ | 3.75 ± 0.05^g^ | 46.93±3.43 |  |

*EC50 values were expressed as the antioxidant amount required to scavenge 50% of DPPH, superoxide anion, and ABTS radical cation.
**TEAC values were expressed as μM/L Trolox equivalents.
***FRAP values were expressed as μM/L Fe (II) ion equivalents.
The TEAC and FRAP values of the compounds, tocopherol, and BHT were determined at a concentration of 2.5 mM.
**** TBARs, as adducts of MDA with 2-thiobarbituric acid, represented the % inhibition of lipid peroxidation.

Data were presented as mean ± standard deviation. A significance level of p < 0.05 was considered. Different letters within the same column indicate statistically significant differences among the data.

#### ^1^ **Determination of DPPH Radical Scavenging Effect**

The DPPH radical scavenging activity of the compounds was determined according to the method developed by Brand-Williams et al. in 1995 [1]. To 0.1 mL of indole derivative solution in dimethylsulfoxide (DMSO) (1.6 - 50 mM), ascorbic acid (0.1 - 0.8 mM) or α-tocopherol (0.1 - 0.8 mM), 3.9 mL of DPPH- solution in methanol (6x10^-5^ M) was added and the mixture was kept in the dark for 30 minutes. Absorbances were read against methanol in a spectrophotometer at 517 nm. Positive and negative controls were run in parallel, with the positive control using standard (α-tocopherol and ascorbic acid) in place of the indole derivative and DMSO as the negative control. The experiment was repeated three times and averaged. DPPH radical scavenging activity was calculated according to the following equation**:**

**1.** W. Brand-Williams, M. E. Cuvelier, C. L. W. T. Berset, *LWT - Food Sci. Technol.* **1995**, *28*, 25.

#### **^2^ Determination of Superoxide Radical Scavenging Effect**

The superoxide radical scavenging effect of the compounds was determined according to the method developed by Aruoma et al. in 1989 [2]. The experiment is based on reading the absorbance at 560 nm of blue - purple colored formazones formed as a result of the reduction of nitroblue tetrazolium (NBT) by superoxide radicals produced by xanthine/xanthine oxidase system. The experiment was carried out in 3 ml reaction medium. 100 L indole derivative (6.25 - 50 mM) or ascorbic acid (0.1 - 1.6 mM), 100 l 30 mM ethylene diamine tetra acetic acid (EDTA) (1 mM), 10 l 30 mM xanthine dissolved in 50 mM KOH (0.1 mM), 100 l 3 mM NBT (0.1 mM) and 2.59 ml 57.5 mM KH_2_PO_4_ - KOH, pH 7.4 (50 mM) were mixed and the reaction was initiated by the addition of 100 l 1U xanthine oxidase. After 5 minutes at 25^o^C, absorbances were read against the blind at 560 nm. The experiment was repeated three times and averaged and the superoxide radical scavenging activity was calculated according to the following equation:

**2.** O. I. Aruoma, B. Halliwell, B. M. Hoey, J. Butler, *Free Radical Biol. Med.* **1989**, *6*, 593.

#### **^3^ Determination of Trolox Equivalent Antioxidant Capacity**

This method, in which the total antioxidant effect of the compounds is investigated, is based on the spectrophotometric measurement of the decrease in color as a result of the removal of ABTS-+ [2,2'-azinobis(3-ethylbenzothiazoline-6-sulfonic acid)] radical, a blue-green colored resistant compound. The total antioxidant activity of the compounds was determined according to the Trolox-exchanged antioxidant capacity (TEAC) method proposed by Re et al. in 1999 [3]. ABTS radical scavenging activity was calculated according to the following equation:

**3.** R. Re, N. Pellegrini, A. Proteggente, A. Pannala, M. Yang, C. Rice-Evans, *Free Radical Biol. Med.* **1999**, *26*, 1231.

**^4^Derivation of the Trolox Standard Curve Equation**

In order to obtain the standard curve equation of trolox, a 10 mM solution of trolox was first prepared and by diluting this solution with 75 mM phosphate buffer (pH 7.4), solutions with concentrations of 1.5 mM, 1 mM, 0.5 mM, 0.5 mM, 0.25 mM and 0.125 mM were obtained. After the TEAC assay was applied to the solutions, the decrease in color was measured spectrophotometrically at 734 nm at 6 minutes and the percentages of ABTS radical cation scavenging activity of trolox were calculated and the experiment was repeated five times.

#### **^5^ Determination of Ferri Ion Reducing Capacity**

Ferri ion reducing power was performed according to the method developed in the study published in 2005 by Chung et al. 0.1 ml of indole derivative (0.4-12.5 mM) or ascorbic acid (0.4-3.1 mM) and α-tocopherol (0.4-6.25 mM) used as standards were mixed with 0.25 ml phosphate buffer (pH= 6.6) and 0.25 ml potassium ferricyanide and incubated at 50^o^C for 20 min [4]. At the end of this time, the reaction was stopped with 0.25 ml TCA, the mixture was centrifuged at 3000 rpm for 5 minutes, 0.25 ml distilled water and 0.5 ml 0.1% FeCl_3_ were added to 0.25 ml supernatant taken in separate tubes and absorbances were read at 700 nm.

**4.** Y. C. Chung, S. J. Chen, C. K. Hsu, C. T. Chang, S. T. Chou, *Food Chem.* **2005**, *91*, 419.

#### **^6^ Determination of Lipid Peroxidation Inhibitory Activity**

To determine the capacity of indole derivatives to inhibit lipid peroxidation, the TBARs method recommended by Patro et al. (2002) and modified by Hamad et al. (2010) was applied [5,6]. An artificial lipid system consisting of liposomes prepared from soy phosphatidylcholine (PC) was used. Liposome suspension wase prepared by sonication of a mixture containing 300 mg PC in 30 mL of Na-phosphate buffer (10 mM, pH 7.4) for 2 hours on ice. To a total volume (1 mL) of a mixture, containing 10 mM potassium phosphate buffer (pH 7.4), 250 μL of the liposome suspension, and 450 μL of 5 mM of indole derivatives or DMSO (control), was added FeCl_2_, H_2_O_2_ and ascorbic acid, each in a final concentration of 125 μM, and incubated at 30 °C for 4 h. α-Tocopherol was used as a reference antioxidant. Following 4 h of incubation, malondialdehyde (MDA), one of the end products of lipid peroxidation, was detected by using the thiobarbituric acid (TBA) reagent, containing 0.375% TBA, 15% trichloroacetic acid (TCA), and 0.25 M HCl. A 500 μL TBA reagent was added to a 250 μL assay mixture. MDA solutions at various concentrations were also reacted with the TBA reagent to generate a standard MDA curve. These mixtures were kept at 100 °C for 15 min. After cooling to room temperature, pink colored TBA-reactive species (TBARs), as adducts of MDA with 2-thiobarbituric acid, were obtained by centrifugation (3000 x*g*, 5 min) as a supernatant, and their absorbance at 532 nm was recorded spectrophotometrically. MDA levels of the assay mixtures were calculated using the standard curve formula of MDA. Lipid peroxidation inhibition (as %) was calculated according to the following formula.

**5.** B. S. Patro, S. Rele, G. J. Chintalwar, S. Chattopadhyay, S. Adhikari, T. Mukherjee, *ChemBioChem.* **2002**, 3, 364.

**6.** İ. Hamad, Ö. Erol-Dayi, M. Pekmez, E. Önay-Uçar, N. Arda, *Plant Foods Hum. Nutr.* **2010**, 65, 44.

#### **^7^ Cytotoxicity Test**

HFF ("Human foreskin fibroblasts") cell line was used in the study. The cells were cultured in 25 cm^2^ culture dishes using heat inactivated 10% FBS ("Fetal Bovine Serum", fetal bovine serum), 1% NEAA ("Non-Essential Amino Acids"), DMEM ("Dulbecco's Modified Eagle's Medium") medium containing 1% antibiotic-antimycotic solution, 1% sodium pyruvate (100 mM), 1% GlutaMAX (200 mM) and 4.5 g/L D-glucose [7] in an incubator (37°C, 5% CO_2_). The cytotoxic effects of 8 compounds (**4g, 4h, 4i, 4j, 4l, 5a, 5b** and **5h**), which were determined to have antioxidant activity at least two *in vitro* tests, were detected by using the MTT method proposed by Mosmann and modified by Önay Uçar et al [8,9]. For this purpose, cells were seeded into 96-well culture dishes and incubated for 24 hours at 37°C and in an oven containing 5% CO_2_. At the end of this period, the medium on the cells that had adhered to the culture dish was removed, and medium containing different concentrations of compounds (only medium for the control group) was added to the experimental group cells and kept in the oven for 48 hours. At the end of this period, the medium on the cells was removed, and 30 µL of MTT stock solution (5 mg/mL, in PBS) was added to each well. Followed by 4 hours of incubation, 150 µL of DMSO was added to each well, and the mixture was shaken until homogeneous. Finally, the absorbance of the colored products was measured in a microplate reader (Multiplate Reader UV/Vis Reader-BioTek), and the % viability value was calculated using the following formula:

7. Freshney, *Culture of Animal Cells: A Manual of Basic Technique*, 4th ed., Wiley-Liss Press, New York **2000**.

8. T. Mosmann, *J. Immunol. Methods* **1983**, 65, 55.

9. E. O. Ucar, N. Arda, A. Aitken, *Genet. Mol. Res.* **2012**, 11, 2801.
